# Genomic Hypomethylation in the Human Germline Associates with Selective Structural Mutability in the Human Genome

**DOI:** 10.1371/journal.pgen.1002692

**Published:** 2012-05-17

**Authors:** Jian Li, R. Alan Harris, Sau Wai Cheung, Cristian Coarfa, Mira Jeong, Margaret A. Goodell, Lisa D. White, Ankita Patel, Sung-Hae Kang, Chad Shaw, A. Craig Chinault, Tomasz Gambin, Anna Gambin, James R. Lupski, Aleksandar Milosavljevic

**Affiliations:** 1 Bioinformatics Research Laboratory, Epigenome Center, Baylor College of Medicine, Houston, Texas, United States of America; 2 Department of Molecular and Human Genetics, Baylor College of Medicine, Houston, Texas, United States of America; 3 Program in Structural and Computational Biology and Molecular Biophysics, Baylor College of Medicine, Houston, Texas, United States of America; 4 Institute of Computer Science, Warsaw University of Technology, Warsaw, Poland; 5 Institute of Informatics, Warsaw University, Warsaw, Poland; 6 Department of Pediatrics, Baylor College of Medicine, Houston, Texas, United States of America; 7 Texas Children's Hospital, Houston, Texas, United States of America; The Hospital for Sick Children and University of Toronto, Canada

## Abstract

The hotspots of structural polymorphisms and structural mutability in the human genome remain to be explained mechanistically. We examine associations of structural mutability with germline DNA methylation and with non-allelic homologous recombination (NAHR) mediated by low-copy repeats (LCRs). Combined evidence from four human sperm methylome maps, human genome evolution, structural polymorphisms in the human population, and previous genomic and disease studies consistently points to a strong association of germline hypomethylation and genomic instability. Specifically, methylation deserts, the ∼1% fraction of the human genome with the lowest methylation in the germline, show a tenfold enrichment for structural rearrangements that occurred in the human genome since the branching of chimpanzee and are highly enriched for fast-evolving loci that regulate tissue-specific gene expression. Analysis of copy number variants (CNVs) from 400 human samples identified using a custom-designed array comparative genomic hybridization (aCGH) chip, combined with publicly available structural variation data, indicates that association of structural mutability with germline hypomethylation is comparable in magnitude to the association of structural mutability with LCR–mediated NAHR. Moreover, rare CNVs occurring in the genomes of individuals diagnosed with schizophrenia, bipolar disorder, and developmental delay and *de novo* CNVs occurring in those diagnosed with autism are significantly more concentrated within hypomethylated regions. These findings suggest a new connection between the epigenome, selective mutability, evolution, and human disease.

## Author Summary

The human genome contains many loci with high incidence of structural mutations, including insertions and deletions of chromosomal segments. This excessive mutability has accelerated evolution and contributed to human disease but has yet to be explained. Segments of DNA repeated in low-copy numbers (LCRs) have been previously implicated in promoting structural mutability in specific disease-associated loci. Lack of methylation (hypomethylation) of genomic DNA has been previously associated with high structural mutability in gibbons and in human cancer cells, but the association with structural mutability in the human germline has not been explored prior to this study. Our analyses confirm the role of LCRs in promoting structural mutability on the genome scale but also reveal a surprisingly strong association of genomic instability with hypomethylation. Specifically, evolutionary analyses reveal that methylation deserts, the ∼1% fraction of the human genome with the lowest methylation in human sperm, harbor a tenfold higher number of structural mutations than genome-wide average. Moreover, the structural mutations in individuals diagnosed with schizophrenia, bipolar disorder, developmental delay, and autism are significantly more concentrated within hypomethylated regions. Our findings suggest a new connection between methylation of genomic DNA, selective structural mutability, evolution, and human disease.

## Introduction

Array comparative genomic hybridization (aCGH) studies [1] and massively parallel sequencing [2] revealed that approximately 10% of the human genome is structurally polymorphic at the submicroscopic scale (<4 Mb), a much larger fraction than affected by single nucleotide polymorphisms (SNPs). Structural mutations that occur in a number of well studied structurally unstable loci cause disease [3]. The discovery of these structurally mutable disease-associated loci gave rise to the concept of genomic disorders [3], [4]. Their detailed analysis revealed the role of non-allelic homologous recombination (NAHR) and low copy repeats (LCR) in mediating recurrent deletions, duplications and inversions [5]. Genome-wide analyses of regions between paralogous LCRs in direct orientation have since led to the successful prediction of novel LCR-mediated genomic disorders [6], reinforcing the role of NAHR and LCRs. A potential role for LCR in inverted orientation has been elucidated recently for a specific type of complex duplication with an embedded triplicated segment in inverse orientation, DUP-TRP/INV-DUP [7].

The process of chromothripsis [8] has been proposed as a model to explain instability in 1–3% of all cancers resulting in a highly complex pattern of genomic rearrangements with multiple CNVs. The patterns of genomic instability observed in cancer have also been observed in complex genomic rearrangements (CGR) in human germline, pointing to similar mechanistic underpinnings [9].

The distribution of structural mutations in the human genome is highly selective, characterized by many hotspots of structural mutability. Evolutionary analyses of recent structural mutations in the human genome reveal that structural mutation hotspots frequently give rise to new LCRs [10], [11], indicating that a significant fraction of the observed association of LCRs and mutability may be explained by the increased production of LCRs at hypermutable loci. The recent discovery of a genome-wide association of LCRs with somatic mutability in cancer [12], and structural breakpoints in the mouse genome independent of LCR homology [13] further support the hypothesis that LCRs may not always cause instability but may preferentially arise at the loci that are inherently mutable both in cancer and in germline.

Recent high-resolution genome analyses of genomic disorder loci revealed complex patterns of rearrangements not consistent with the NAHR mechanism [14], [15], [16], [17]. The mechanisms causing mutability in such structurally mutable hotspots remain elusive. Microhomologies and other sequence-level features point to the role of Fork Stalling and Template Switching (FoSTeS) and Microhomology-Mediated Break-Induced Replication (MMBIR) mechanisms [16] in the processing and repair of one-ended, double-stranded DNA [18]. However, these are repair mechanisms, are not causing mutations, and have not explained the highly selective distribution of structural mutability nor predicted genomically unstable loci.

Multiple independent lines of evidence point to a possible role of the epigenome in structural mutability. Chromatin modifications are known to play a significant role in chromosome maintenance [19], including DNA repair [20], [21], and recombination [22], [23]. Chromatin and the epigenome regulate mutability at smaller scales, including increased mutability of 5-methyl cytosine [24], retroposon silencing [25], [26], [27], and preferential retrotransposition into specific chromatin states [28]. Genome-wide hypomethylation has been repeatedly observed in structurally unstable cancer genomes [29], [30]. Mutations in the methyltransferase *DNMT3B* have been shown to cause hypomethylation and genomic instability in juxtacentromeric regions in humans [31]. Mutations in the mouse homolog of methyltransferase *DNMT1* have been shown to cause genomic instability [32]. Analyses of the structurally hypermutable genomes of gibbon species revealed association of hypomethylation with structurally mutable loci [33]. Finally, the recent discovery of the role of the DNA-break inducing base-excision repair pathway in genomic demethylation of primordial germ cells (PGCs) during fetal development in mouse [34] provides a possible mechanistic link between genomic hypomethylation and genomic instability in the mammalian germline.

Genomic hypomethylation and LCR-mediated NAHR are therefore the two genome architectural features shown to be associated with structural changes. We here systematically examine and quantitate these associations. To assess the degree of association of germline methylation levels with structural instability, we examine four sperm methylome maps, including two high read coverage (15× combined coverage) from a recent study [35] and two maps we obtained by performing whole-genome bisulfite sequencing of sperm samples from two anonymous donors at low coverage (2.5× combined coverage). To improve detection of structural mutations associated with LCRs and NAHR, we perform a comprehensive detection of human LCRs in the human genome and design an aCGH array for diagnostic use in the BCM Medical Genetics Laboratories (BCM-MGL) targeting NAHR susceptible regions between directly oriented paralogous LCRs (DP-LCRs) with size larger than 10 Kbp, separated by a distance less than 10 Mb of unique genomic sequence. We combine evidence of structural mutations from the following three sources: 1) human-specific genomic rearrangements; 2) structural polymorphisms in the human population, including copy-number variation (CNV) data from BCM-MGL and publicly available CNV data sets [36], [37], [38]; and 3) recent disease studies of schizophrenia [39], bipolar disorder [40], developmental delay [41], and autism [42]. Our analyses reveal a pattern of association of structural mutability with germline hypomethylation comparable in magnitude to the association between structural mutability and LCR-mediated NAHR.

## Results

### Construction and Comparative Analysis of Sperm Methylomes by Whole-Genome Bisulfite Sequencing

To examine a potential association between germline methylation and structural mutability in humans, we first derived two sperm methylome maps by sequencing at combined 2.5× genome coverage (one at 1.2× and the other at 1.3×) bisulfite-treated genomic DNA samples extracted from the sperm of two anonymous donors. Methylation levels were calculated for each of the 28,705 non-overlapping 100 Kbp windows covering the hg18 human genome assembly as the ratio between the number of methylated CpGs and the total number of CpGs sampled in reads mapping within the window. Windows with less than 20 CpG sampling events were removed from the subsequent analysis to avoid bias due to low sequence mappability. Both samples had more than 95% of windows with reads covering more than 40% of the CpGs within the window (Figure S7B). Due to the low 2.5× combined coverage, the methylation levels of individual CpGs could not be determined with accuracy, but the average methylation levels at 100 Kbp level of resolution could be determined with high accuracy. Specifically, the methylation level of >98% windows was determined with <10% error with >95% probability (Table S10). The two methylomes were highly concordant at 100 Kbp level of resolution (linear correlation coefficient = 0.96). For the purpose of our analyses, an average sperm methylome at 2.5× coverage was constructed as an average of the two concordant methylomes. Methylation deserts were operationally defined as the 100 Kbp windows with the lowest 1% methylation level in the average sperm methylome. A 5% threshold was also used for some analyses, as noted below.

We repeated our analyses using an independently obtained pair of sperm methylomes generated by Molaro *et al.* [35] from bisulfite sequencing data at a combined 15× genome coverage. To ensure deep sampling of CpGs in each window, only windows with more than 100 mapped reads and more than 100 CpG sampling events at 15× coverage were included in the subsequent analyses. To facilitate comparison, both combined methylomes (at 2.5× coverage and at 15× coverage) were represented as methylation averages across the same set of 100 Kbp windows tiling the human genome.

The 15× methylome showed high correlation with the 2.5× methylome at the 100 Kbp resolution (r = 0.82, p-value<2.2e-16). Methylation deserts discovered at 2.5× coverage using methylation percentile rank thresholds of 1% and 5% significantly overlapped those discovered at 15× coverage (Figure S21), indicating relatively stable genomic localization of methylation deserts across individuals.

### Comprehensive Identification of Potentially NAHR–Associated LCRs in the Human Genome

It has been suggested that directly-oriented paralogous LCRs (DP-LCRs) with high similarity, large size, and in close proximity would be most likely to mediate NAHR, resulting in deletions or duplications identifiable by aCGH [1], [3], [5], [6], [43]. We designed, implemented, and validated a new computational method for comprehensively detecting LCRs and DP-LCRs (see Materials and Methods: Computational Pipeline for LCR Identification). The method achieves higher sensitivity than previously applied methods [44] by using *k*-mer frequency sequence information to detect and cluster LCRs without remmatoving (repeat-masking) high copy-number repetitive elements (Materials and Methods: Whole-Genome Self-Comparison and Text S1 section 1.1). In total, 268 regions between DP-LCRs were identified (Figure S3), a greater than two-fold increase over previously reported estimates (Text S1 section 1.2 and Figure S4).

### Human-Specific Evolutionary Structural Rearrangements Associate More Strongly with Methylation Deserts Than with DP–LCR Regions

We next examined the association of evolutionarily recent structural rearrangements in the human genome with both DP-LCR loci and germline hypomethylation. Assuming nearly neutral evolution [45], the distribution of structural variants that have accumulated in the human lineage since the branching of chimpanzee can be used as an indicator of structural mutability. By applying the Genomic Triangulation method [46] to genomic data from four non-human primate species (chimpanzee, rhesus macaque, orangutan and marmoset) and the human reference genome we detected 522 human-specific structural rearrangements (Materials and Methods: Identification of Human-Specific Rearrangements).

The human-specific structural rearrangements were found to be highly associated with LCRs (six-fold enrichment, permutation test, p≈10^−3^), much higher than with other examined genomic features such as repetitive elements (*Alu*: 0.89-fold; LINEs: 1.1-fold; Microsatellites: 1.2-fold). One-third of the rearranged regions were actually human LCRs, indicating a significant fraction of the association may be explained by segmental duplication events that produce LCRs. The rearrangements were found to associate specifically with DP-LCR loci to a lesser degree (three-fold enrichment, permutation test, p≈10^−3^).

**Figure 1:**
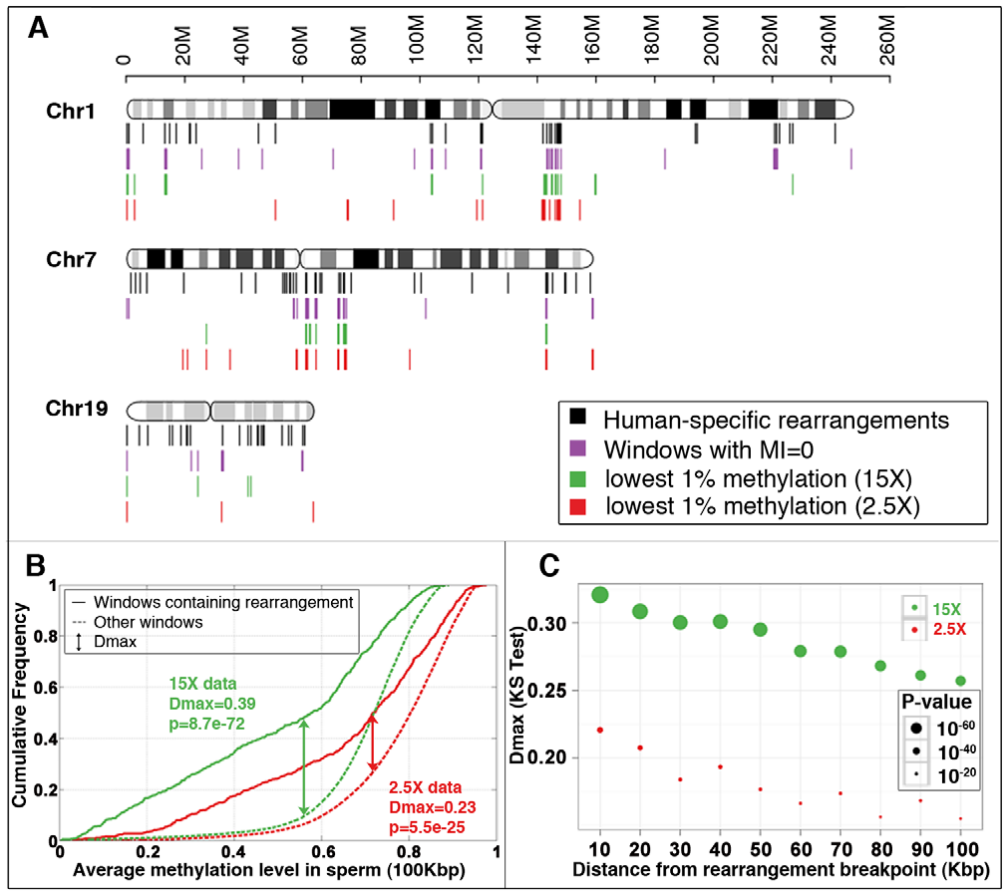
Association between methylation deserts and human-specific structural rearrangements. (A) Locations of human-specific structural rearrangements (black), 100 Kbp windows with methylation index value 0 (violet), 100 Kbp windows with lowest 1% sperm methylation at 15× coverage (green) and 2.5× coverage (red) for three representative chromosomes. (See Figure S18 for a whole genome view). (B) Cumulative sperm methylation distribution and the Kolmogorov-Smirnov statistics for 100 Kbp windows containing rearrangements (solid line) and the rest of the windows (dashed line) at 15× coverage (red) and at 2.5× coverage (red). (C) Simulation test of extent of hypomethylation in the regions flanking human-specific structural rearrangements. Distribution of methylation levels for 10 Kbp regions sampled at increasing distances (from 10 Kbp to 100 Kbp) from the 522 human specific structural rearrangements is compared to the distribution of methylation levels of randomly picked segments with matching sizes within the same chromosome (100 random samplings for each rearrangement). The same analysis is performed for methylomes at 15× coverage (green) and 2.5× coverage (red). *D_max_* and significance *p*-value were determined using the Kolmogorov-Smirnov test.

A striking association was detected between human-specific structural variants and hypomethylation. First, the methylation deserts comprising a total of 1% of the human genome contain ∼10% of the human-specific structural rearrangements, a tenfold enrichment (Figure 1A). Second, genome-wide comparison indicates a highly significant inverse association of human-specific rearrangements with methylation levels (Kolmogorov-Smirnov test, D_max_ = 0.23, p≈10^−24^) (Figure 1B). Additional permutation-testing experiments that are not based on fixed window size indicate that approximately 23% (D_max_ = 0.23) of human-specific rearrangements associate with hypomethylation (Figure S5A). The significance of this association gradually decreases with increasing distance from rearrangements (Figure 1C), suggesting that hypomethylation and structural mutability co-localize within relatively small chromosomal segments. The association could not be accounted for by considering a number of other potentially confounding factors including CpG islands, chromosomal bands, telomeric/centromeric locations and sex chromosome bias (Text S1 section 3; Tables S8, S9).

**Figure 2:**
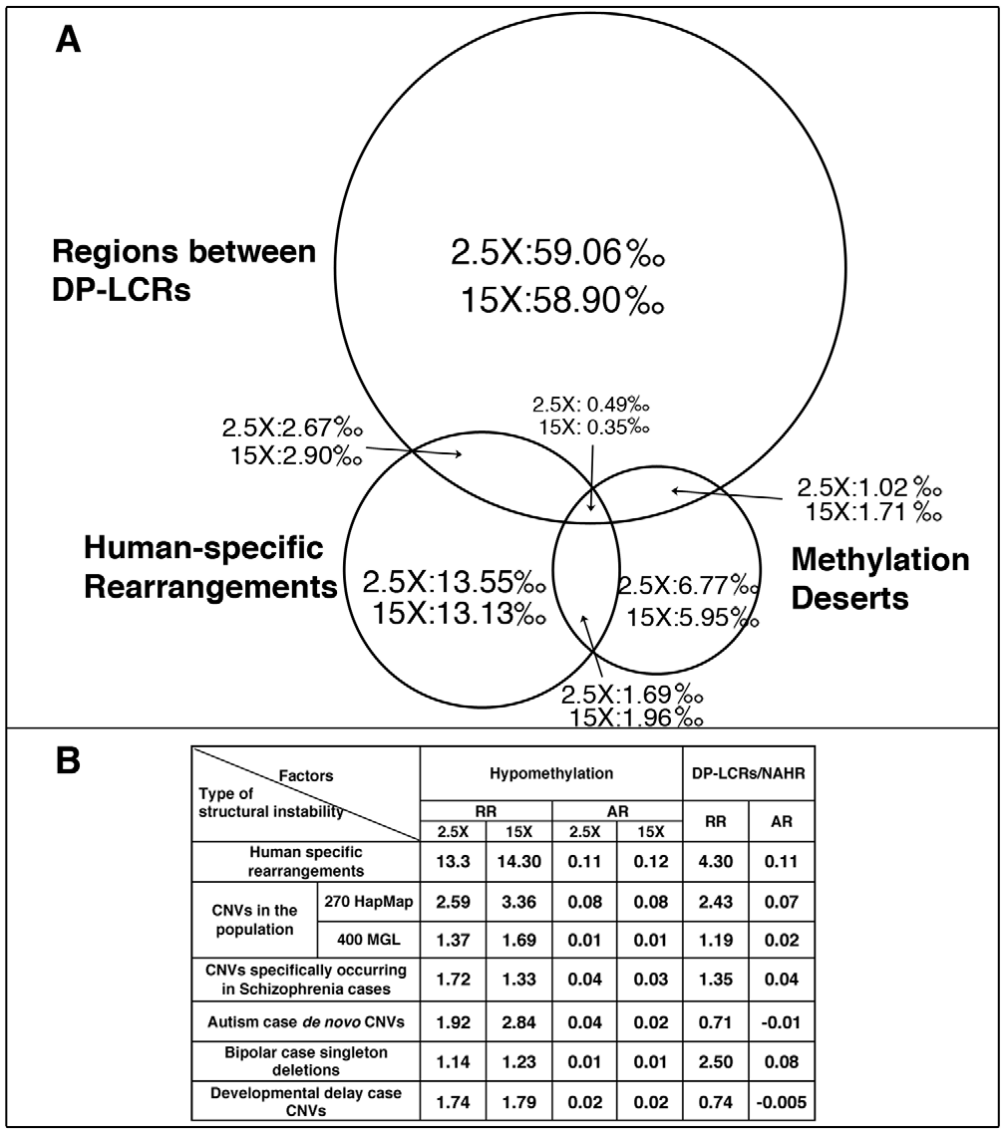
Statistical risk analysis of structural mutability due to hypomethylation and DP–LCRs. (A) Venn diagram of 100 Kbp windows classified into one or more of the following three categories: (i) windows containing human-specific structural rearrangements; (ii) windows within methylation deserts (windows with lowest 1% methylation at 2.5× or 15× coverage); and (iii) windows containing regions between DP-LCRs. Numbers within the circle areas indicate fraction (per mil) of the genome occupied by the specific groups of windows. (B) Statistical relative risk (RR) and statistical attributable risk (AR) of structural instability for hypomethylation and DP-LCRs (the first row corresponds to A).

We next directly compared the relative strengths of association of hypomethylation and DP-LCRs with human-specific rearrangements. The 100 Kbp windows covering the genome were each assigned to one or more of the following groups: (a) windows containing human-specific rearrangements; (b) windows that are methylation deserts; and (c) windows containing regions between DP-LCRs. The Venn diagram in Figure 2A illustrates proportions of windows across the three groups, based on which we calculated the statistical relative and attributable risks of rearrangements due to hypomethylation and DP-LCRs in Figure 2B (first row). Note that both genomic features confer significantly increased statistical risk, but the statistical relative risk due to hypomethylation is markedly higher than the risk due to DP-LCRs.

### Estimation of Germline Methylation Levels Using a Methylation Index Calculation

Methylation levels in sperm are only a partial indicator of methylation levels in the whole human germline. To further examine the association between germline methylation and structural mutability in humans directly, one would ideally be able to measure DNA methylation in the entire male and female germline lineages, which are highly dimorphic [47]. To practically address this issue, we pursued an indirect approach by estimating methylation levels in the human germline (an average of male and female germlines), using the methylation index (MI) model [48] (Materials and Methods: Methylation Index Calculation at 100 Kbp Level of Resolution).

Approximately 20% of the methylation deserts (defined as the lowest 1% methylation levels in sperm) occur within the 1.5% fraction of windows with the lowest MI score (MI = 0), an indication that methylation deserts detected in sperm overlap substantially with hypomethylation in the germline as a whole (Figure S6A). The windows with MI = 0 contain ∼15% of the human-specific structural rearrangements, a similar tenfold enrichment as we observed for methylation deserts defined based on the sperm methylomes (Figure 1A).

The sperm methylation scores of windows with MI = 0 show a bimodal distribution (Figure S6B), the lower mode including 35% with low methylation levels (<5%) in sperm and the higher mode is comprised of the remaining 65% that appear to have normal methylation levels in sperm. Because the higher mode could not be explained by obvious ascertainment biases (Materials and Methods: Examination of MI Ascertainment Biases), we hypothesize that this mode may either indicate hypomethylation specific to the female germline, given that male and female germline methylation patterns are highly dimorphic [47], or may be due to other germline hypomethylation detected by MI that is absent from sperm. Similar bimodal distribution was observed at 15× coverage (Figure S9B).

As additional controls, five publicly available methylomes obtained by whole-genome bisulfite sequencing [49], [50] of human stem cells and fibroblasts were also compared over the same set of 100 Kbp windows. Methylation levels in sperm showed much higher correlations with the methylation levels in embryonic stem cells than with fibroblasts (Table S2), consistent with the more differentiated state of fibroblasts. Importantly, the methylation levels in sperm samples have higher correlations with the germline MI scores than either stem cells or fibroblasts (Table S2). Moreover, the bimodal distribution of hypomethylated regions is unique to sperm (Figure S9), consistent with sperm being the closest representative of the human germline.

### Copy Number Variants (CNVs) Associate More Strongly with Hypomethylation than with DP–LCRs

To examine structural mutability during more recent evolutionary time, we turned to the analysis of Copy Number Variants (CNVs) in the human population. De-identified aCGH data were collected from 400 human DNA samples analyzed by the BCM Medical Genetics Laboratories (BCM-MGL; http://www.bcm.edu/geneticlabs/). These data were originally produced at BCM-MGL using a custom designed, whole-genome oligo-aCGH chip with a genomic distribution of probes more densely spaced between DP-LCRs as well as with lower but even distribution for the remaining regions of the genome (Materials and Methods: aCGH Probe Set Design and Analysis of CNVs in 400 MGL Samples). Approximately 12,000 non-unique CNVs seen in more than one individual larger than 500 bp were identified. More than 60% of the CNVs were not in public structural variation databases (Figure S10).

**Figure 3:**
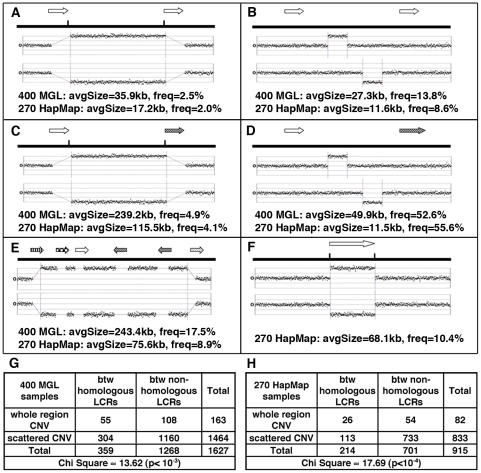
Major patterns of CNVs in relation to LCRs (arrows with same texture indicates paralogous LCRs). (A) CNVs involving whole regions between DP-LCRs. (B) Scattered CNVs (CNVs covering <40% of the distance between LCRs) between DP-LCRs. (C) CNVs involving whole regions between non-paralogous LCRs. (D) Scattered CNVs between non-paralogous LCRs. (E) Complex patterns of CNVs extending over various LCR groups and intervening regions. (F) CNVs overlapping LCRs. (G–H) Contingency tables summarizing the counts of CNVs observed between LCRs, corresponding to A, B, C and D. The CNVs between paralogous LCRs tend to involve the whole region (as illustrated in A, corresponding to counts in top left cells in G and H), a signature of NAHR involving paralogous LCRs.

A significant enrichment of LCRs (permutation test, three-fold enrichment, p≈0.01) was found around the CNVs. When CNVs occurred between DP-LCRs, they were more likely to span the intervening region, a signature of NAHR, than those between non-paralogous LCRs (2-fold enrichment, p≈0.001 by chi-square test, Figure 3G). However, such CNVs represent a small fraction (∼2.5%, Figure 3A) of all CNVs.

**Figure 4:**
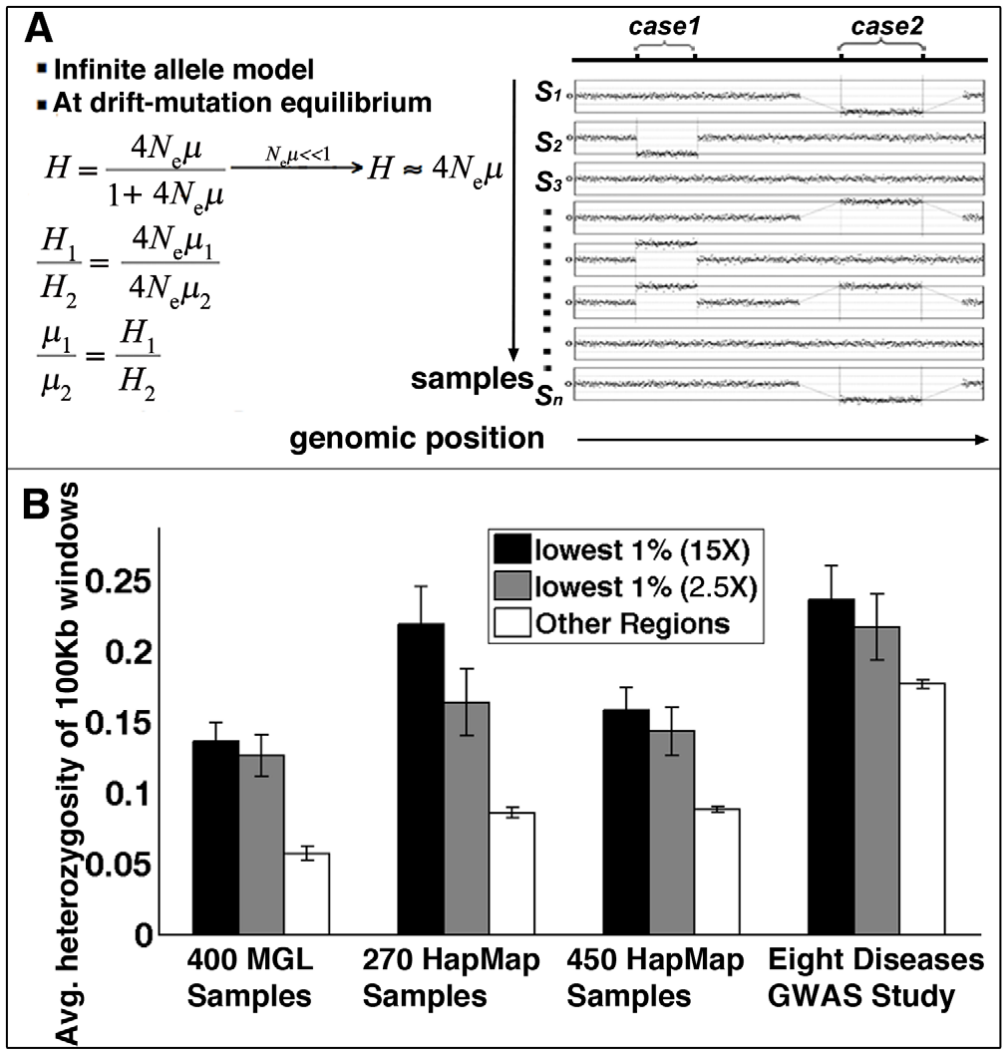
Structural mutability assessed by structural heterozygosity. (A) Under the infinite allele model, assuming structural mutations are neutral and at drift-mutation equilibrium, mutation rates are proportional to heterozygosity rates. (B) Comparison of average CNV heterozygosity rates (data from four studies) within (black for methylomes at 15× coverage, gray for methylomes at 2.5× coverage) and outside (white) methylation deserts. Error bars represent standard deviation of CNV heterozygosity rates in corresponding regions.

We next examined any potential association between LCRs and structural mutability using structural heterozygosity as a proxy. Assuming structural mutations are neutral, under the infinite allele model [51], the rate of structural heterozygosity is proportional to the mutation rate. Structural mutability can therefore be assessed using the rate of structural heterozygosity as a proxy (Figure 4A). Our results indicate that genome-wide structural mutability is directly correlated with LCR density and particularly with the LCRs that contain high copy-number repetitive elements (Figure S11).

Following a similar approach as in Materials and Methods: Human-Specific Evolutionary Structural Rearrangements Associate More Strongly with Methylation Deserts Than with DP–LCR Regions, we next compared the distributions of sperm methylation levels for 100 Kbp windows containing CNVs and for those not containing any CNVs. The Kolmogorov-Smirnov test results indicate that the windows containing CNVs have significantly lower methylation in sperm (Figure S13). Permutation testing indicates that an excess of 9% of the CNVs is explainable by hypomethylation (Kolmogorov-Smirnov Dmax = 0.09, Figure S5B). Association analysis also indicates a higher statistical relative risk due to hypomethylation than due to DP-LCRs (Figure 2B).

We next compared structural mutability in methylation deserts with mutability in other genomic loci using structural heterozygosity rate as a proxy. The comparison indicated that the methylation desert loci have higher average structural heterozygosity rates (Figure 4B). The Kolmogorov-Smirnov test also indicates significant excess heterozygosity of CNVs in hypomethylated regions (Figure S14A).

### Publicly Available CNV Data Validate Association between Hypomethylation and Structural Mutability

As an independent test for any potential association between hypomethylation and structural mutability, we performed analyses analogous to those discussed in the previous section using the following three publicly available CNV datasets: (i) aCGH data obtained from 270 HapMap samples using high-resolution Affymetrix SNP 6.0 arrays [36]; (ii) aCGH data obtained from 450 HapMap samples using tiling oligonucleotide microarrays [37]; and (iii) CNV data generated on 19,000 samples [38] in a study of the role of common CNVs in eight common human diseases. The dataset (i) complements the 400-sample BCM-MGL data because it detects CNVs that overlap LCRs, and it provides high probe resolution in regions that are not associated with LCRs. Despite the bias away from known polymorphisms in the design of the custom array used to generate the 400-sample BCM-MGL dataset (Materials and Methods: aCGH Probe Set Design and Analysis of CNVs in 400 MGL Samples, Text S1 section 5 and Figure S12), analyses of the data set (i) confirmed the relative strengths of association of structural mutability with NAHR and with hypomethylation identified using the BCM-MGL data, as indicated in Figures 2B, 3, 4, Figure S14, and Table S7. All three (i–iii) datasets confirmed significantly higher average heterozygosity rates of CNVs in methylation deserts (Figure 4). However, dataset (iii), which was biased against rare structural alleles [38], showed no significant difference in overall heterozygosity rate distributions between CNVs in the methylation deserts and the rest of the CNVs (Figure S14D), suggesting that rare variants may account for a significant fraction of association.

In summary, despite the differences in array technologies, array design biases, and sample sets applied to the arrays, our analyses repeatedly point to a significant association of hypomethylation and structural mutability.

### Analysis of Methylomes in Germline and Embryonic Stem Cells Indicates Association of Structural Mutability with Germline-Specific Hypomethylation

We next asked if the association between structural mutability and hypomethylation is specific to germline, using the embryonic stem cell line H1 methylome [50] as a control. Germline methylation was assessed using the sperm methylomes both independently and in combination with the methylation index, as summarized in the five columns in Table 1.

**Table 1:** Enrichment of structural mutability in hypomethylated regions determined by the germline methylation index (MI = 0) and whole-genome bisulfite sequencing of human sperm DNA (at 2.5× and 15×).

	Enrichment fold (p-value)	Windows with MI = 0	sperm lowest 5%		MI>0 & sperm<5%		MI = 0 & sperm>5%		MI = 0 & sperm<5%	
			2.5×	15×	2.5×	15×	2.5×	15×	2.5×	15×
**Human evolution**	**Human-specific rearrangements**	10.2 (3.9e-106)	5.1 (2.0e-77)	7.1 (3.7e-226)	3.6 (2.6e-35)	5.2 (1.3e-78)	6.0 (1.3e-28)	2.6 (7.0e-3)	12.5 (5.1e-76)	12.1 (4.0e-134)
**Structural polymorphisms**	**270HapMap CNVs**	2.7 (4.2e-13)	1.8 (5.7e-8)	2.3 (1.6e-26)	1.7 (6.5e-5)	2.4 (2.0e-22)	2.5 (2.8e-7)	2.1 (3.2e-3)	3.4 (2.1e-9)	3.2 (1.2e-13)
	**450HapMap CNVs**	2.0 (1.6e-12)	1.3 (6.0e-4)	1.5 (1.9e-16)	1.3 (6.3e-4)	1.4 (8.6e-8)	1.8 (5.9e-8)	1.3 (1.9e-2)	2.0 (3.1e-6)	2.3 (1.5e-17)
	**WTCCC CNVs**	2.6 (2.3e-24)	1.6 (1.1e-9)	2.2 (3.0e-56)	1.5 (3.1e-7)	2.2 (1.5e-34)	2.5 (1.8e-17)	1.8 (1.1e-3)	2.5 (6.3e-9)	3.2 (1.3e-31)
	**400MGL CNVs**	3.1 (4.2e-10)	2.6 (4.1e-6)	2.1 (1.1e-10)	2.5 (4.4e-6)	1.6 (2.3e-12)	3.9 (1.4e-5)	1.7 (6.8e-3)	2.8 (3.8e-3)	2.9 (4.2e-10)
**Disease studies**	**Schizophrenia case-specific rare CNVs**	4.0 (2.5e-5)	2.7 (1.2e-2)	2.0 (2.7e-2)	2.6 (4.7e-2)	1.8 (7.3e-2)	3.8 (5.0e-3)	5.3 (3.5e-3)	5.7 (4.5e-3)	3.5 (2.0e-3)
	**Autism ** ***de novo*** ** CNVs in cases**	3.9 (1.1e-3)	4.1 (1.3e-4)	3.8 (5.0e-3)	4.5 (1.8e-5)	2.1 (7.3e-5)	3.5 (1.8e-2)	2.7 (1.4e-3)	8.2 (1.7e-5)	8.4 (5.5e-3)
	**Bipolar case-specific singleton deletions**	1.7 (8.3e-2)	2.3 (1.2e-2)	2.0 (9.9e-2)	1.8 (1.8e-2)	1.1 (9.9e-1)	0.6 (7.3e-1)	0.5 (4.7e-1)	1.4 (1.1e-1)	2.7 (9.3e-3)
	**Developmental delay rarecase-specific CNVs**	2.3 (2.4e-83)	2.9 (4.5e-302)	3.3 (9.9e-80)	2.5 (1.1e-298)	1.9 (1.6e-190)	3.4(5.8e-231)	4.8 (1.4e-276)	1.6 (1.4e-3)	2.1 (3.2e-3)

P-values are calculated using Chi-square test. Significance of enrichment for hypomethylation in rows marked “Human evolution” and “Structural polymorphisms” was calculated relative to randomly selected windows throughout the genome. For rows marked “Disease studies”, significance of enrichment for hypomethylation was calculated using the following controls: for the schizophrenia study, using control-specific rare CNVs; for the autism study, using inherited rare CNVs found in cases; for the bipolar study, using control-specific singleton deletions. The developmental delay study, significance of enrichment for hypomethylation in windows containing rare (<1% population frequency) CNVs found in cases was established using the CNVs found in control group as controls.

Recall that for windows with MI = 0, the sperm methylation scores showed a bimodal distribution (Figure S6B). As indicated in Table 1, significant enrichment of structural mutability could be observed for windows with MI = 0, and for both lower and higher modes of these windows. The enrichment observed in the higher mode (Table 1, column “MI = 0 & sperm>5%”) suggests the role of hypomethylation that is possibly present in the female germline and captured using the MI measurement but not present in sperm.

The windows containing rearrangement/variation showed much lower methylation levels in the sperm methylome (Figure S15A–S15C). In contrast, an association with methylation levels in H1 could not be detected for the CNVs, except that windows containing human-specific evolutionary rearrangements did show association (Figure S15D–S15F). We found significant negative correlation between the methylation scores in sperm and the heterozygosity rates (CNVs from 400 MGL samples: r≈−0.15, p≈10^−9^; CNVs from 270 HapMap samples: r≈−0.20, p≈10^−10^). In contrast, no significant correlation between the H1 methylation scores and the CNV heterozygosity rates was detected.

We next examined the difference in methylation levels between sperm and H1. As illustrated in Figure S16, the difference shows even stronger association with structural mutability than the absolute methylation levels in sperm. This result rules out possible ascertainment biases due to low mappability of sequencing reads in potentially unstable and repetitive hypomethylated regions. It also suggests that structural mutability is associated with germline-specific hypomethylation.

### Structural Variants Identified Specifically in Schizophrenia Patients Concentrate within Hypomethylated Regions

We next examined the distribution of rare CNVs detected in the recent large-scale study by the International Schizophrenia Consortium [39]. CNVs in 3,391 individuals diagnosed with schizophrenia and 3,181 controls were identified and analyzed using Affymetrix SNP arrays. The study found that the individuals in the affected group have 15% more rare variants. We asked if the excess of variants in the affected group tends to occur in regions with low germline methylation levels.

We first compared the distribution of the methylation levels for 100 Kbp windows containing the CNVs in the affected group with the distribution of methylation levels for windows not containing any CNVs. The same procedure was performed for the CNVs in the control group. Both the affected and control CNVs showed lower methylation. A significant enrichment of low MI values (Kolmogorov-Smirnov test, p≈10^−5^) was found for the affected group (Table S3), while no significant enrichment was found for the control group.

We next identified those CNVs found only in the affected group and those found only in the control group. The two subsets were then further classified as being within or outside of regions showing lowest 5% methylation levels in sperm. The chi-square test indicates a 3-fold enrichment (p≈10^−3^) within low methylation regions of variants identified only in the affected group compared to those found only in the control group (Table 1). Similar enrichment was found in regions with MI = 0 (Table 1).

### Large Deletions Identified Specifically in Bipolar Disorder Patients Concentrate within Hypomethylated Regions

We next examined distribution of CNVs identified in a recent bipolar disease study [40]. The study identified CNVs in 1001 bipolar disease cases and 1034 controls. An excess of large singleton deletions was found in cases relative to controls. We examined methylation of singleton deletions found only in bipolar cases to the methylation of the deletions found only in controls. As indicated in Table 1, compared to control-specific deletions the case-specific singleton deletions were enriched over 2-fold (p<1e-3 by Chi-square test) within the 100 Kbp windows having lowest 5% methylation levels in sperm.

### *De Novo* Structural Variants in Autism Cases Are Concentrated within Hypomethylated Regions

A recent autism spectrum disorders (ASDs) study [42] found a higher burden of rare CNVs in ASD patients. Trio analyses established that some of the CNVs were not present in parental genomes and were classified as *de novo*. We asked if the rare and *de novo* CNVs detected in the autism cases and controls associated with low methylation levels.

The regions containing rare CNVs in both the cases and controls showed significant enrichment for both low methylation levels in sperm and for low MI values, when compared with regions without any rare CNVs (Table S3). The CNV variants identified only in the cases showed an approximately two-fold enrichment in hypomethylated regions compared to those found only in controls, but the enrichment did not reach statistical significance threshold due to a small number of variants detected (data not shown).

Analysis of *de novo* and inherited CNVs found in cases revealed highly significant enrichment within hypomethylated regions of *de novo* relative to inherited CNVs. The enrichment was observed within hypomethylated regions in sperm (<5%), within windows of MI = 0, and especially in regions that met both criteria (Table 1).

### Structural Variants Identified in Children with Developmental Delay Concentrate within Hypomethylated Regions

A recent study by Cooper *et al.* [41] identified CNVs in 15,767 children with intellectual disability and various congenital defects (cases) and in 8,329 unaffected adults (controls). We examined the enrichment of rare (<1% population frequency) case-associated CNVs within the windows with lowest 5% methylation in sperm relative to CNVs found in controls. Using Chi-square test, we observed a significant 2.9-fold enrichment of the case-specific rare CNVs (p = 2.78e-124) compared to the control CNVs. Out of the 59 pathogenic CNVs identified in this study, 12% are located in the methylation deserts, a 4.7-fold (p = 3.3e-5) enrichment compared with the control CNVs. Specific sub-classifications of phenotypic information was reported for almost half of the cases, including 575 cases with cardiovascular defects, 1,776 with the epilepsy/seizure disorder, 1,379 with the autism spectrum disorder and 3,898 with craniofacial defects [41]. We therefore repeated the same chi-square test for each sub-class, and observed enrichment of CNVs associated with each sub-phenotype vs. all control CNVs (11).

### Methylation Deserts Are Enriched for Fast-Evolving Developmental Regulatory Loci

Analysis of genomic features in the methylation deserts showed no enrichment for SINEs, LINEs or microsatellites (Figure S1C). Higher GC content was found for methylation deserts than elsewhere (Figure S1A), which may be due in part to the somewhat higher number of CpG islands in these regions than expected by chance (Figure S1C). Methylation deserts also showed higher average sequence conservation than the rest of the genome (Figure S1B). However, conserved coding sequences were slightly under-represented (0.9 fold), and pseudogenes were over-represented (2 fold, Figure S1C). Overall, genes were under-represented (0.7 fold) except for homeobox, cadherin, and histone families, all of which were highly enriched in methylation deserts (Table S1). Using the sperm gene expression data from previous studies by Pacheco *et al.* [52], we detected enrichment within methylation deserts of those genes that are highly expressed in sperm (Text S1 section 2).

We next examined enrichment of promoters categorized by their CpG content into high-, intermediate- and low-CpG content promoters by Weber *et al.* [53]. We first observed a significant negative correlation between the methylation level and average CpG content across all 100 Kbp windows (r = −0.35, p = 2.5e-270). However, methylation deserts were not enriched for promoters with high CpG content (Table S6). Those with low CpG content showed slight under-representation in the methylation deserts (0.65 fold). Interestingly, those with intermediate CpG content, which were also referred to as “weak CpG islands” and known to be more prone to de novo methylation during differentiation [53], [54] showed 3-fold enrichment in the methylation deserts (Table S6).

According to Mohn *et al.*, almost all bivalent promoters (marked by both H3K27me3 and H3K4me2 during cellular differentiation) contain CpG islands, and a significant proportion of weak CpG promoters are bivalent and more likely to be methylated *de novo* [54]. We therefore examined the bivalent promoters as identified by Ku *et al.* [55] and found their 2.6-fold enrichment in the methylation deserts (Table S6). The promoters that were both bivalent and had intermediate CpG content showed four-fold enrichment (Table 2).

**Table 2:** Enrichment of various regulatory features in methylation deserts detected using permutation test or chi-square test. Enrichments for an expanded set of regulatory features are included in Table S6.

Regulatory features	Fold-enrichment in methylation deserts	p-value
**Two fast-evolving transcription factor clusters ** [**59**]	15	<1e-3
**GRB target genes ** [**58**] ** vs. random segments**	12	<1e-10
**GRB target genes vs. ‘bystander’ genes ** [**58**]	9.2	1.42e-43
**GRB target genes vs. other CpG island-overlapping genes outside GRBs ** [**58**]	33	1.41e-146
**Hyperconserved CpG domains with low COCAD scores ** [**56**]	37.6	<1e-4
**Bivalent promoters with intermediate CpG content ** [**55**]	4	<1e-3

Because the Polycomb repressive complex 2 (PRC2) is known to regulate bivalent promoters, we next examined the distribution of PRC2 binding regions within methylation deserts, focusing specifically on the hyperconserved CpG domains (HCGDs) identified by Tanay *et al.* [56]. Tanay *et al.* used the COCAD (context-based CpG analysis of divergence) score to compare the actual rate of human–chimpanzee CpG divergence to the predicted rate. The HCGDs with low COCAD scores showed extensive overlap with regions bound by Polycomb repressive complex 2 (PRC2). Of the 194 non-overlapping genomic regions corresponding to HCGDs with COCAD scores below −5 (P<1E−6), a total of 60 (31%) are located in the methylation deserts (2.5× coverage), showing a 37.6-fold enrichment compared to the genomic background as determined by permutation testing (Table 2).

Because tissue-specific regulation may involve changes in CpG methylation levels, we next investigated whether the methylation deserts are enriched for regions that are methylated in a tissue-specific manner. Toward this goal, we first examined the methylation data gathered at 1,413 CpG loci across 217 samples from 11 different human tissue types by Christensen *et al.* [57]. The CpG loci were divided into a group within germline methylation deserts and a group that did not fall within methylation deserts. Each CpG locus was assigned a score measuring the variation of methylation level across 11 tissues [57]. Kolmogorov-Smirnov test showed that CpG loci within the methylation deserts are significantly enriched for inter-tissue variability (Figure S22). To rule out the possibility that the excess variation is due to causes other than developmental regulation, the distributions of CpGs that exhibit aging-related variation and of those that exhibit environment-related variation were examined. None of the two groups of CpGs exhibited any preferential distribution within methylation deserts, indicating the methylation difference among cell lineages is more likely to be related to developmental regulation.

We next examined whether the methylation deserts are enriched for regions involved in regulation of tissue-specific gene expression using the set of 269 putative genomic regulatory blocks (GRBs) and their target genes identified in the human genome by Akalin *et al.* [58]. The GRB target genes are most often transcription factors involved in embryonic development and differentiation. We examined the enrichment of GRB target genes or GRBs themselves in the methylation deserts (lowest 1% sperm methylation at 2.5× coverage) using randomly selected genomic segments as controls. The GRB target genes showed 12-fold enrichment in the methylation deserts (p<1e-10). The GRBs on the other hand, showed around 2.8 fold enrichment in methylation deserts, of which those that are multiple target GRBs showed a 4.4 fold enrichment (both p<1e-3). Comparing distribution of other CpG island-overlapping genes outside GRBs to GRB target genes, by chi-square test we observed an extremely high 33-fold enrichment of GRB target genes within the methylation deserts (p∼1.41e-146, Table 2). As an additional control, we examined ‘bystander’ genes defined by Akalin *et al.* as those intertwined with highly conserved non-coding elements but whose expression and function are unrelated to those of the GRB target genes. GRB target genes were enriched in the methylation deserts 9.2-fold relative to the ‘bystanders’ (p∼1.42e-43, by chi-square test, Table 2).

Because methylation deserts are hotspots of evolution, we examined enrichment within methylation deserts of transcription factors (TFs) reported by Vaquerizas *et al.* [59] to be fast evolving in primates. We first applied permutation test to the coding sequences of all the ∼1300 manually curated sequence-specific TFs and observed a 3.75 fold enrichment for their coding sequences in the methylation deserts (p<1e-3). We then examined the TFs within two clusters reported by Vaquerizas *et al.* [59] to be fast evolving in primates and detected an even higher 15-fold (p<1e-3) enrichment (Table 2).

## Discussion

Combined evidence from evolutionary, population-genetic and disease studies supports strong association between germline hypomethylation and selective structural mutability. Genome-wide, both relative and attributable risks of structural mutations due to methylation deserts are at least comparable to the corresponding statistical risks due to LCR-mediated NAHR. Our results show that 23% of human-specific evolutionary rearrangements are associated with hypomethylation. Methylation deserts comprise a total of 1% of the genomic sequence and contain about 10% of the 522 submicroscopic human-specific structural rearrangements identified by primate genome comparisons.

The evolutionary findings are generally consistent with the results of analyses of CNVs in the human population. Our analysis reveals a two-fold genome-wide enrichment for deletions and duplications between DP-LCRs, the signature pattern of LCR-mediated NAHR. While the enrichment is statistically significant, the fraction of structural variation statistically attributable to NAHR is small, approximately 2.5%. We show that methylation deserts exhibit higher association with CNVs (∼9%) and contain a disproportionately high fraction of CNVs that have high structural heterozygosity. The population-based analyses reveal less striking enrichment patterns than the evolutionary analyses. This may be explained by the fact that population based studies were generally of lower resolution (array-based, unlike sequence-based evolutionary analyses), were limited to copy-number changes, and were biased against rare variants.

By demonstrating a higher association of structural mutability with hypomethylation than with NAHR, our results underscore the potential relative contribution of the role of microhomology-mediated break-induced repair in structural genomic instability [37] which is consistent with replication based mechanisms such as FoSTeS [14], MMBIR [18], and serial replication slippage (SRS) [16] rather than NAHR.

Our results are consistent with the concept of a structural selective “mutability profile”, an epigenomic phenotype marked by the variation in germline methylation levels along the genome. Three questions regarding this mutability are of particular interest: heritability, mechanism, and evolution.

First, does inter-individual variation in methylation-associated selective mutability profiles exist and if it does, is it heritable? As a first step toward answering these questions, we have generated preliminary results tentatively suggesting that inter-individual variation in selective structural mutability may be associated with methylation deserts (Text S1 section 6 and Figure S17).

The second open question is the mechanism behind the selective mutability profile. One conceivable mechanism is genetic variation in DNA-break inducing base-excision repair enzymes involved in germline-specific demethylation [34]. Another possibility may involve unrepaired DNA breaks associated with active transcription because methylation deserts are highly transcribed in germline. Yet another possibility may be that transcription factors mediate structural rearrangements by bending chromatin, creating looping structures and DNA breaks, analogously to the role played by estrogen and androgen receptors in mediating structural instability in hormonally regulated tumors [60], [61], [62]. One specific possibility opened by this model is that selective structural mutability may be affected by the cellular and organismal environment and may be controlled experimentally or even therapeutically.

Finally, assuming selective mutability profile variation is heritable, the question of its evolution arises (for a recent survey of the topic of “evolution of evolvability” see [63]). Specifically, does selective mutability evolve mostly neutrally by random drift? If not, what may be the nature of selection pressure acting on it? Assuming that selection indeed plays a role, it is useful to consider the payoff (higher probability of developing a favorable mutation that ultimately becomes fixed in the population) and risk (of mutation causing disease). A selective mutability profile with excess mutability concentrated in the loci with low payoff/risk ratios would then be less likely to produce mutations that ultimately become fixed than a mutability profile with mutability concentrated in the loci with high payoff/risk ratios. The latter would therefore be favored by selection.

One testable corollary of this payoff/risk model is that *de novo* mutations will tend to cause diseases related to the phenotypes that are under positive selection in the human population. Assuming that brain function is under selection in the human population, this corollary predicts high incidence of brain-related diseases such as schizophrenia, bipolar disorder, autism, epilepsy, developmental delay and cranial features due to rare and *de novo* mutations. Our findings that the rare and *de novo* CNV variants in the individuals suffering from these diseases indeed concentrate within methylation deserts is consistent with this corollary. These findings suggest a novel type of connection between evolution and human disease [64].

The payoff/risk model is also consistent with highly mutable loci being responsible for tissue-specific phenotypes. This is because a mutation in a locus regulating a tissue-specific phenotype may not confer much risk to other tissues. The enrichment within methylation deserts that we observed for genes with tissue-specific patterns of expression and for transcription factors involved in cellular differentiation is therefore consistent with this payoff/risk model.

## Materials and Methods

Methylation and structural variation data used in this study can be accessed and visualized via the Genboree Project page and Genboree Genome Browser (http://genboree.org/java-bin/project.jsp?projectName=Germline%20Methylation&isPublic=Yes).

### Sequencing and Methylome Construction of Bisulfite-Treated Human Sperm DNA Samples

Two anonymous human sperm samples were collected from a local fertility clinic. Genomic DNA was isolated from the samples using the PureLink Genomic DNA kit (Invitrogen, CA, USA). A total 5 ug of DNA was sonicated with 30×30 s, 30 s interval, using Bioruptor (Diagnode, NJ, USA). Sonicated DNA was end repaired using the End-It Kit (Epicentre, WI, USA) and A-tailed in a 50 µl reaction containing 1 mM dATP mix, 10 U of 3′ to 5′ exo- Klenow DNA polymerase (NEB, MA, USA). Adaptor ligation was performed in 50 µl reaction containing 300 mM pre-methylated adapters and 1000 Unit T4 DNA polymerase and incubated at 16°C overnight. Adaptor-ligated DNA was subjected to a size selection on a 3% NuSieve 3∶1 agarose gel. DNA marker lanes were excised from the gel and stained with SYBR Green (Invitrogen, CA, USA). 250–350 bp slices were excised from the unstained gel and purified using MinElute spin column (Qiagen, CA, USA). Size-selected fragments were bisulfite-treated using the EpiTect Bisulfite Kit (Qiagen, CA, USA) with minor modifications by adding 5 more cycles (5 min 95°C followed by 90 min at 60°C). After bisulfite conversion, DNA was eluted in 40 µl EB buffer and 0.8 µl DNA was used for analytical PCR reactions to determine the minimum number of PCR cycles required to get enough material for sequencing. Final PCR products were purified on MinElute columns (Qiagen, CA, USA) and assessed on 4–20% polyacrylamide Criterion TBE Gel (Bio-Rad, CA, USA) and quantified using Qubit fluorometer (Invitrogen, CA, USA). The libraries were sequenced on the Illumina Genome Analyzer II (one lane for each sample) following the manufacturer's instructions.

The Pash 3.0 software [65] was used to map the resulting reads to the reference human genome (NCBI 36.1/UCSC hg18). Pash 3.0 maps bisulfite reads natively. Reads were hashed considering the space of all possible kmers (e.g. for ATCT, the kmers ATCT, ATCC, ATCCC, ATCCT will be hashed). The forward and the reverse strands of the reference genome were streamed against the kmer reads hash, and regular mapping was applied. T's in the reads can map to both C's and T's in the reference. Pash 3.0 performs gapped mapping, being sensitive to both indels and base pair substitutions. Only reads that map uniquely and with at least 90% identity were used for subsequent analysis. Duplicate reads were removed across the same library. In total, 82.39% of the reads for sample1 and 83.02% for sample2 passed quality filters, achieving genome coverage at 1.3× and 1.2× respectively.

Each chromosome of the reference human genome (NCBI 36.1/UCSC hg18) was divided into 100 Kbp windows, excluding assembly gaps. The methylation levels in each sample were estimated by examining every CpG dinucleotide within each read mapping into each of the 28,705 windows. The methylation level of a window was estimated by dividing the number of methylated CpGs by the total number of CpGs found in reads mapping within the window. Windows with less than 20 CpG sampling events were excluded from consideration. The average of the two methylation maps was used as a representation of the sperm methylome to compare with the inferred germline methylation index.

For control purposes, five other methylomes of human embryonic stem cells and fibroblasts were constructed from publicly available whole-genome bisulfite sequencing data [49], [50], using the same pipeline.

### Computational Pipeline for LCR Identification

#### Whole-Genome Self-Comparison

The human genome sequence (NCBI build 36.1/UCSC build hg18) was compared against itself to identify similar sequence fragments using the Pash (Positional Hashing) comparison method [65], [66], [67]. Pash implements Positional Hashing, a parallelizable method for sequence comparison based on *k*-mer representation of sequences (Figure S2A) instead of the usual single-base representation (*k* = 13 in this study). To improve the sensitivity in the presence of base mismatches, the actual sampling pattern was 21 bp long, sampling 13-mers and including 8 unsampled positions. To avoid hitting highly repetitive sequences (LINEs, SINEs, etc.), *k*-mers overrepresented in these high copy-number repetitive elements (HCRs) were ignored. The frequency distribution of the 13-mers with a frequency >10 in the HCRs (data from UCSC RepeatMasker track http://genome.ucsc.edu/cgi-bin/hgTrackUi?g=rmsk) was compared with their frequency distribution in the whole genome sequence. The *k*-mers that were significantly enriched in the HCR sequences (chi-square test, multiple comparisons corrected with FDR<0.1) were excluded. For the self-comparison of the genome, the fragment length was set at 500 bp.

#### Reciprocal Matching and Merging of Fragments into Pairwise LCRs

The matches between fragments identified in the previous step were post-processed by applying a “reciprocal best match” filter. For a match between two fragments to pass the filter, the two fragments were required to appear on each other's list of top 50 matches (50 is the maximum number of members in one paralogous group in the UCSC segmental duplication track http://genome.ucsc.edu/cgi-bin/hgTrackUi?g=genomicSuperDups) with either list not containing more than 1000 matches.

The filtered list of matching fragments then went through a merging step where multiple segments close to each other in genomic location were merged into one LCR block if their matching partners were also located within a certain range (span<1 Kbp, radius<250 bp), and if the PASH similarity score density ( = score/chunk length) exceeded a certain threshold (>0.05). The merging was performed in both direct and reverse orientations, producing a list of pairwise LCRs (Figure S2B).

#### Clustering of Pairwise LCRs

All the identified pairwise LCRs were clustered using their *k*-mer features and overlaps (Figure S2C). The clustering was based on two criteria: first, a *k*-mer content similarity, measured by {1−[*No. of kmerDiff+log(1+sizeDiff)]/[(No. of kmerInBothSets)*]} (*kmerDiff*−number of *k*-mers that occur in one pair but not the other; *sizeDiff* - size difference between the two pairs; *kmerInBothSets* – number of *k*-mers that occur in both pairs); and second, any positional overlap between members from different pairs. Clustering according to the two criteria was applied recursively to all paired up segments until all of them have been compared and clustered. Finally, the following previously suggested similarity threshold filter was applied [44] to select qualified clusters: containing LCRs with length ≥1 Kbp, and sharing identity ≥90% (calculated using BLAT [68]).

#### Identification and Validation of Direct Paralogous LCRs (DP–LCRs)

The full set of LCRs was further filtered to identify a subset, which we refer to as DP-LCRs that are directly-oriented intrachromosomal paralogous LCRs ≥10 Kbp in size, sharing ≥95% similarity and located within 10 Mbp distance of each other.

To validate DP-LCR prediction output by the PASH pipeline, DP-LCRs were independently predicted using a pipeline designed by a subgroup of our team (TG and AG) and implemented using the MUMmer [69] software. The pipeline includes dividing genome sequence into overlapping contigs, aligning each contig using MUMmer, filtering identified segments according to criteria of DP-LCRs, and merging results from all contigs. MUMmer was utilized with parameters settings: exact match length ≥25 bp, length between two adjacent matches in a cluster ≤1 Kbp, cluster length ≥3 Kbp, and distance of alignment extension = 2 Kbp. Options “-*nooptimaize*”, “-*maxmatch*” and “-*nosimplify*” were selected. MUMmer's prediction of direct paralogous LCRs sharing identity at 80%, 90%, and ≥92% were combined to compare with the PASH pipeline output. The DP-LCRs identified by both methods were used in subsequent analyses.

### Methylation Index Calculation at 100 Kbp Level of Resolution

The MI model is based on the fact that in mammals DNA methylation predominantly occurs in CpG dinucleotides, increasing the probability of transitions to TpG or CpA dinucleotides. The MI calculation by Sigurdsson *et al.* [48] implicitly uses mutability of CpGs in the human genome as an indicator of methylation in the germline. We apply this method of by integrating four million non-redundant SNPs from the HapMap project. Methylation index values were calculated for the same set of 100 Kbp windows used for sperm methylome construction to facilitate comparison.

#### Methylation Index Calculation

Each of the 100 Kbp windows across the genome assembly was assigned a methylation index as an indicator of methylation levels in the germline, which was computed as defined by Sigurdsson *et al.* [48]. Briefly, a SNP was defined to be methylation-associated (mSNP) if a C/T or G/A SNP was located within a CpG dinucleotide (in either orientation), with ancestral allele being C or G respectively. The ancestral allele was determined as the orthologous base in the chimpanzee or macaque genomes. The mSNPs were identified using the HapMap SNPs track (based on International HapMap Project release 27, available from the UCSC genome browser http://genome.ucsc.edu/cgi-bin/hgTrackUi?db=hg18&g=hapmapSnps). Methylation index (MI) was calculated by the following formula: 
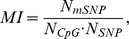
where *N_mSNP_* denotes the number of observed mSNPs within a window, *N_CpG_* - the number of CpGs, *N_SNP_* - the number of SNPs, and (*N_CpG_⋅N_SNP_*) is a number directly proportional to the expected number of mSNPs within the window assuming uniform methylation levels across the genome. Windows without any SNP, therefore without a valid MI value, were excluded from all analysis.

#### Examination of MI Ascertainment Biases

The sperm methylation scores of windows with MI = 0 show a bimodal distribution (Figure S6B), the lower mode including 35% with low methylation levels (<5%) in sperm and the higher mode is comprised of the remaining 65% that appear to have normal methylation levels in sperm. One could expect that if the windows with MI = 0 were due to low probing density, the windows within the higher mode would have fewer SNPs or CpGs. However, we examined potential biases in MI estimation due to variations in the number of SNPs, CpGs, read coverage (Figure S6CD), or sampling events (Figure S7BD) and found no significant difference between the two modes, ruling out the possibility that the two modes may be explained by variation in mappability or shallow sampling. In addition, a simulation experiment showed that the statistical variance of methylation estimates due to CpG sampling of windows with MI = 0 was a relatively small fraction of biological variance in methylation observed between the two sperm methylomes (Figure S8). We therefore hypothesize that the higher mode may either indicate hypomethylation specific to the female germline, given that male and female germline methylation patterns are highly dimorphic [47], or may be due to other germline hypomethylation detected by MI that is absent from sperm.

In addition to comparing the two modes, windows with MI = 0 were analyzed for the enrichment of potential confounding genomic features, evolutionary conservation signatures, and specific gene families. The results of these analyses are discussed in detail in Text S1 section 4 and summarized in Figure S19, and Tables S4 and S5.

### Identification of Human-Specific Rearrangements

The sites of likely human-specific structural rearrangements were identified using the Genomic Triangulation method [46]. Non-human primate fosmid end sequences (FESs) from chimpanzee (CHORI-1251 library), rhesus macaque (Washington University Genome Sequencing Center (WUGSC) MQAD library), orangutan (WUGSC PPAD library) and marmoset (WUGSC CXAG library) were downloaded from the NCBI Trace Archives (http://www.ncbi.nlm.nih.gov/Traces/). The FESs were mapped to the human genome (NCBI 36.1/UCSC hg18) using BLAT [68] with the parameters: *tileSize* = 11, *minMatch* = 2, *minScore* = 100, *minIdentity* = 0, *maxIntron* = 50. Alignment scores were calculated for BLAT mappings using the parameters: *match* = +2, *mismatch* = −1, *gap opening* = −2, *gap extension* = −1. BLAT mappings with an alignment score less than 200 were removed from consideration. BLAT results were also filtered to remove ambiguous reads anchoring to more than 12 locations with an alignment score within 5% of the top alignment score. FESs that mapped at a distance consistent with fosmid clone insert size (25–50 Kbp) and in correct orientation were used to infer orthologous blocks. FESs were allowed to consistently map to multiple locations so that shared segments could be covered. Overlapping orthologous blocks were merged, based on genomic coordinates, into “matepair chains”. Matepair chain gaps due to human assembly gaps were removed. The remaining 522 matepair chain gaps indicated sites of likely human-specific structural genomic rearrangements.

### aCGH Probe Set Design and Analysis of CNVs in 400 MGL Samples

A 105 K Agilent oligo CGH array was designed for the purpose of routine diagnostic CNV testing at MGL. Probe sequences were chosen from the Agilent Technologies HD CGH database. Oligos were searched for multiple homologies to the human genome (NCBI 36.1/UCSC hg18) to avoid cross-hybridization. Only unique oligos were selected for the array design.

The whole genome sequence was divided into three types of regions covered with probes at different densities. The genes between DP-LCRs associated with genomic disorders were probed at the highest probe density (1 probe/10 Kbp, or at least 10 probes/gene for small genes). The second-highest probe density (1 probe/15 Kbp, or at least 10 probes/region) was assigned to the identified regions between DP-LCRs. These regions were required to be gene-containing, with a length from 1 Kbp to 10 Mbp, and flanked by direct paralogous LCRs that are ≥10 Kbp in length, and sharing ≥94% similarity. Probes with the same density were also assigned to the regions within the genome sequence coordinates of BAC/P1 artificial chromosome clones that had already been validated for clone arrays used in clinical practice (Baylor College of Medicine (BCM) BAC Chromosomal Microarray V6, including 1472 BAC and PAC clones for over 270 known genetic syndromes, 41 unique subtelomeric regions, 43 unique pericentromeric regions, and the mitochondrial genome). The third probe density (1 probe/31 Kbp) was assigned to all the other regions in the genome, so-called “backbone” regions. All the probes were selected to avoid the highly repetitive elements, the LCRs, and the known CNVs in major public databases: TCAG Database [70] of Genomic Variants hg18.v1 (http://projects.tcag.ca/variation/), and UCSC Structural Variation database (http://genome.ucsc.edu/cgi-bin/hgTrackUi?db=hg18&g=cnp).

De-identified array intensity data obtained from 400 human DNA samples were made available to us by MGL. The data were analyzed using the Circular Binary Segmentation (CBS) method [71], which splits array intensity data along the genome sequence into segments with equal copy number that are significantly different from the neighboring regions.

### Simulation Tests of Association between Hypomethylation and Genomic Rearrangements or Structural Variations

To determine the extent of association of hypomethylation with human-specific rearrangements/CNVs and to avoid possible artifacts due to the fixed 100 Kbp window size for sampling, the distribution of the methylation levels for the structural rearrangements/CNVs was compared to the distribution of the methylation levels of randomly picked segments (100 random samplings for each of the rearrangements/CNVs) of matched sizes on the same chromosomes (Figure S5AB, Table S7 rightmost two columns). To examine the extent of hypomethylation in the regions flanking rearrangements, the average methylation level for 10 Kbp regions sampled at increasing distances (from 10 Kbp to 100 Kbp) from rearrangement breakpoints were compared with 10 Kbp regions at corresponding distances from the randomly selected segments across the same chromosome (Figure 1C, Figure S20B, Figure S23).

### Statistical Risk Analysis of Structural Changes Potentially Attributable to Hypomethylation and DP–LCRs

To estimate the potential contribution of hypomethylation and DP-LCRs regions to the occurrence of structural rearrangements/CNVs, the 100 Kbp windows covering the genome were each assigned to one or more of the following groups: (a) windows containing structural rearrangements/CNVs; (b) windows that are methylation deserts; and (c) windows containing regions between DP-LCRs. Statistical relative and attributable risks were calculated using intersections among these groups or their complements, with the universal set defined as all windows. Using corresponding letters to represent frequencies of these groups and their complements, the statistical relative risk of rearrangements/CNVs of hypomethylation was calculated as 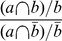, and the statistical attributable risk was calculated as 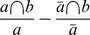. Similarly, the statistical relative and attributable risks of rearrangements/CNVs as effect of DP-LCRs can be estimated by substituting *b* with *c* in the above formulas.

### CNV Heterozygosity as An Indicator of Structural Mutability

Assuming that mutations are neutral, under an infinite allele model for populations at drift-mutation equilibrium, for any two loci in the genome, the ratio of heterozygosity rates *H_1_* and *H_2_* is equal to the ratio of mutation rates *μ_1_* and *μ_2_* [72] (Figure 4A). Therefore, the relative mutation rates at different loci can be estimated by observed relative heterozygosity rates. Structural heterozygosity rates were defined as follows. The normal copy number signal was interpreted as a homozygous major structural allele and any signal other than normal, either gain or loss, was interpreted as indicating presence of minor structural allele. The structural heterozygosity rate at one locus was calculated as *2pq* (*p* = frequency of normal copy number state; *q* = frequency of abnormal copy number state). Since subsets of the 400 MGL samples and the HapMap samples contained trios or father/mother-offspring pairs, the following correction was applied to related samples: if aberration from normal at the same locus was found for related samples (parent and child), its occurrence was counted only once for each related sample trio/pair when calculating allele frequency.

### Functional Annotation Clustering of Genes and Enrichment Analyses

Only genes with valid RefSeq IDs that were detected within CNV heterozygous segments were considered for functional classification. The Database for Annotation, Visualization, and Integrated Discovery (DAVID [73], http://david.abcc.ncifcrf.gov) was used to perform functional annotation enrichment analysis. The enrichment analysis was performed by interrogating the gene lists against the Gene Ontology Biological Process (GOBP), Gene Ontology Cellular Compartment (GOCC), Gene Ontology Molecular Function (GOMF), cell signaling pathways (KEGG Pathway) and the Swiss-Prot/Protein Informatics Resource (SP-PIR) databases. Using all human RefSeq genes as background, the gene categories with significant EASE score (<0.01) and Benjamini correction value (<0.1) in any of these databases were reported as enriched.

To compare gene enrichment within specific structural mutability levels, genes with different CNV heterozygosity rates as detected by the oligo array data were binned into lists, each list corresponding to CNV heterozygosity rates in the range [x, x+0.1) where x took values from 0 to 0.4 in increments of 0.02. Each gene list was analyzed using DAVID as described above.

The tool GFINDer [74] was used for the genetic diseases and clinical phenotypes enrichment analysis. GFINDer exploits textual information within the Online Mendelian Inheritance in Man (OMIM) database. All human Entrez genes were used as background, and resulting categories with p-value less than 0.05 were reported. Tests both without any p-value correction and with FDR correction were applied.

### Ethics Statement

This research did not involve Human Subjects. All data and materials obtained from humans were either anonymized or de-identified prior to use in this research project.

## Supporting Information

Figure S1 Comparison of genomic features in methylation deserts (MD, red) at 2.5× coverage and other regions with MI>0 (nonMD, blue) in the genome. Density plots of (A) GC content; and (B) sequence conservation. (C) Enrichment of various features in methylation deserts, and correlations between the features frequencies and sperm methylation levels across the 100 Kbp windows.
(PNG)

Figure S2 PASH pipeline for LCRs prediction. (A) PASH [67] divides the problem of whole genome comparison into groups of comparison diagonals (*L*-fragment length, set to 500 bp), which can be processed in parallel. For each group, each position along each diagonal is compared between the sequences sequentially using *k*-mers (*k* set to 13). (B) Reciprocal filtering select matching pairs of fragments identified in step A if they appear on each other's list of top 50 matches, then proximal fragments and their matching partners are merged into segments. (C) Identified pairwise LCRs from B were clustered into groups according *k*-mer content similarity and positional overlaps.
(PNG)

Figure S3 Comparison of regions between direct paralogous LCRs (DP-LCRs, length ≥10 Kbp, identity ≥95%, <10 Mbp apart) identified by our method and by Sharp *et al.* [6] (A) Locations of regions between DP-LCRs identified by our method (right-side of each chromosome ideogram), and those identified in the previous study (left-side). The heights of the bars indicate sizes of these regions. (B) Number and length coverage of the regions between paralogous LCRs identified by our method (black), compared with previous study (gray). (Four categories: (i) all regions between paralogous LCRs; (ii) regions between DP-LCRs; (iii) regions between paralogous LCRs and overlapping with genes; and (iv) regions between DP-LCRs and overlapping with genes). (C) Size distributions of regions between DP-LCRs identified by our method (solid color) compared with results from previous study (hatched color), in terms of number (left) and length (right). Small-(1 Kbp, 1 Mbp], white; Medium-(1 Mbp, 5 Mbp], gray; Large-(5 Mbp, 10 Mbp], black.
(PNG)

Figure S4 Factors contributing for increased detection of regions between DP-LCRs compared with previous study. 39% of the regions that we detect but are absent from the previous study occur between the newly identified LCRs that are enriched for HCRs. 35% of the novel regions occur between the newly clustered paralogous LCRs. 11% of the novel regions occur because of the different ways of calculating identity. 15% of the novel regions occur because of other factors, such as difference between genome builds on which the two studies were carried out.
(PNG)

Figure S5 Permutation tests examining association between germline hypomethylation (at 2.5× coverage) and (A) human-specific structural rearrangements (B) CNVs detected in the 400 MGL samples. Kolmogorov-Smirnov (KS) tests comparing the distribution of the sperm methylation levels for the 522 human specific structural rearrangements in (A) and CNVs in (B) (solid lines) and the distribution obtained by randomly picking segments with matching sizes within the same chromosome (based on 100 random samplings for each evolutionary rearrangement or CNV) (dashed lines). The KS test statistic Dmax shows the greatest discrepancy between the two distributions.
(PNG)

Figure S6 Sperm methylation levels (obtained by whole-genome methylation sequencing at 2.5× coverage) of 100 Kbp windows with methylation index MI = 0. (A) Cumulative distributions of sperm methylation levels for windows with MI = 0 (red) and the other windows (blue). The Kolmogorov-Smirnov (KS) statistic indicates significant difference between the two distributions. (B) Density plots of sperm methylation level for windows with MI = 0 (red) and the other windows (blue). The black arrow marks methylation level threshold separating the lower mode including ∼35% of the windows with MI = 0 (orange) and the higher mode including ∼65% of the windows with MI = 0 (green). (C–D) The two modes (indicated marked by orange and green lines matching respective orange and green areas under the two modes in (B)) have similar distribution of SNPs (C) and CpGs (D).
(PNG)

Figure S7 Density plots of the number of CpG dinucleotides sampled by bisulfite sequencing of sperm (at 2.5× coverage) in windows with MI = 0. (A) Histogram and density plots of CpG sampled in all windows with MI = 0. On average there are 787 CpG sampling events per window, with 95% of the MI = 0 windows having at least 20 CpG sampling events. (B) Density plots of number of CpG sampled per MI = 0 window. The two curves correspond to the two modes identified in Figure S6B are colored orange and green correspondingly. (C) Histogram plots for percentage of CpGs in each 100 Kbp window with at least 20 reads mapped from the two sperm samples being sequenced. (D) Density plots of the number of reads mapped in each 100 Kbp window with MI = 0. The two curves, colored orange and green, correspond to the two modes in Figure S6B and the two curves in (B).
(PNG)

Figure S8 Scatter plots comparing true and simulated methylation scores of the two sperm samples (jointly covered at 2.5× read coverage) in 100 Kbp windows with MI = 0. (A) Linear regression of the actual scores from the two samples, with goodness of fit r^2^ = 0.76. (B) Results of a simulation experiment examining differences in methylation scores due to statistical variability assuming binomial sampling of CpGs, the statistical variation being a function of the number of CpG sampling events per window *n* and methylation levels *p*. The scatter plot indicates the results of 1000 iterations simulating the sampling process in windows with MI = 0 using binomial model *B(n,p)*, where *n* is the number of CpG sampling events in each window and *p* is the probability of CpG being methylated in the same window. The averaged r^2^ for all simulations is 0.93, with a standard deviation 0.01. The combined evidence from (A) and (B) indicates that of the total variability between the two sperm samples (1−r^2^ = 1−0.76 = 0.24), less than one third (1−r^2^ = 1−0.93 = 0.07) is due to statistical variation. Inter-individual variation may accounts for a fraction of the residual variation (0.17).
(PNG)

Figure S9 Comparison of methylation status in windows with MI = 0 (red) and other regions with MI>0 (blue) in sperm (A at 2.5× coverage and B at 15× coverage), embryonic stem cells (C), and fibroblasts (D). The left lower mode of the MI = 0 set is uniquely present in sperm, which is most closely related to human germline.
(PNG)

Figure S10 Venn diagram intersecting CNV loci identified from the 400 MGL samples using our custom Agilent array (dark gray), CNVs identified from the 270 HapMap samples using the Affymetrix SNP 6.0 array [36] (light gray), and CNVs from the TCAG database [70] (A) hg18.v1, the version that was available when the array was designed. (B) The same as (A) but with TCAG database version hg18.v8 and UCSC Structural Variation track (white). The numbers indicate total lengths of loci in basepairs.
(PNG)

Figure S11 Correlation coefficients between structural heterozygosity rates and various properties of regions between paralogous LCRs: the size of the flanking paralogous LCRs, the sequence identity of the flanking paralogous LCRs, the distance between paralogous LCRs, a factor combining the previous three properties (Identity×Size/Distance), the density of surrounding LCRs, and the HCRs content of surrounding LCRs.
(PNG)

Figure S12 Distribution of structural heterozygosity rates and enrichment of functional gene annotations for CNVs detected in two datasets. (A–B) Distribution of structural heterozygosity rates for CNVs between DP-LCRs (solid line) and elsewhere (dashed line) in (A) 400 MGL samples and (B) 270 HapMap samples. (C–D) Functional gene annotation categories with highest enrichment scores at different CNV heterozygosity rates in (C) 400 MGL sample set and (D) 270 HapMap samples.
(PNG)

Figure S13 Association between germline hypomethylation (2.5× coverage) and structural polymorphism in the human population. Kolmogorov-Smirnov tests comparing sperm methylation levels distribution of 100 Kbp windows containing CNVs detected in the 400 MGL samples (solid line) and the rest of the windows (dashed line).
(PNG)

Figure S14 Kolmogorov-Smirnov tests comparing CNV heterozygosity rates in methylation deserts (2.5× coverage, 100 Kbp windows) and elsewhere in the genome for (A) 400 MGL samples; (B) 270 HapMap samples [36]; (C) 450 HapMap samples [37]; (D) 19,00**0** samples from eight common diseases GWAS study [38].
(PNG)

Figure S15 Association between structural variation and methylation in sperm (2.5× coverage) and H1 embryonic stem cells [50]. (A-sperm, D-H1): Kolmogorov-Smirnov tests comparing methylation score distribution of 100 Kbp windows containing human-specific structural rearrangements (solid line) and the rest of the windows (dashed line). (B-sperm, E-H1): Kolmogorov-Smirnov tests comparing methylation score distribution of 100 Kbp windows containing CNVs detected in the 400 MGL samples (solid line) and the rest of the windows (dashed line). (C-sperm, F-H1): Kolmogorov-Smirnov tests comparing methylation score distribution of 100 Kbp windows containing CNVs detected in the 270 HapMap samples (solid line) and the rest of the windows (dashed line).
(PNG)

Figure S16 Kolmogorov-Smirnov (K-S) statistics obtained by comparing 100 Kbp windows containing structural variants and the rest of the windows. The 100 Kbp windows were assigned three different methylation scores: (1) methylation difference between sperm and H1 (dark green); (2) absolute methylation score in sperm at 2.5× coverage (light green); and (3) methylation difference between sperm (2.5×) and IMR90 (yellow). For all three type of scores, using K-S statistics we compared (i) the distribution of methylation level of 100 Kbp windows containing structural variants and (ii) the distribution of methylation scores of other windows. The bars with positive values indicate lower methylation scores in sperm. Specifically, windows containing structural variants show more negative methylation difference between sperm and H1 or between sperm and IMR90 (i.e. more hypomethylated in sperm), or smaller absolute sperm methylation scores (green bars).
(PNG)

Figure S17 Increased concentration of CNVs from highly mutable samples in hypomethylated regions (2.5× coverage). (A) aCGH data are ranked by the total number of CNVs detected in each sample, as an indicator of mutability. (B) KS test comparing mutation number per sample in methylation deserts with lowest 1% sperm methylation level at 2.5× coverage (purple) vs. other regions (gray). (C) KS test comparing mutation number per sample in windows with MI = 0 (purple) vs. other regions (gray).
(PNG)

Figure S18 A whole genome visualization of the location of, human-specific structural rearrangements (black), windows with MI = 0 (violet), windows showing lowest 1% methylation in 15× data (green) and methylation deserts (windows showing lowest 1% methylation in our 2.5× data, (red).
(PNG)

Figure S19 Comparison of genomic features in windows with MI = 0 (red) and other regions with MI>0 (blue) in the genome. Density plots of (A) CpG dinucleotide; (B) GC content; (C) SNP density; and (D) sequence conservation. (E) Enrichment of various features in windows with MI = 0, and correlations between the features frequencies and methylation index across the 100 Kbp windows.
(PNG)

Figure S20 Permutation tests examining germline hypomethylation (measured by MI) within and around human-specific structural rearrangements. (A) Permutation testing of association between germline hypomethylation and human-specific structural rearrangements. Kolmogorov-Smirnov (KS) test comparing the distribution of the methylation index for (i) the 522 human specific structural rearrangements (solid line); and, (ii) randomly picked segments with matching sizes within the same chromosome (100 random samplings for each rearrangement) (dashed line). The KS test statistic Dmax shows the greatest discrepancy between the two distributions occurs at MI = 0. (B) Simulation test of extent of hypomethylation in the regions flanking human specific structural rearrangements. Dmax and significance values from KS tests show difference between the distribution of the methylation index for 10 Kbp regions sampled at increasing distances (from 10 Kbp to 100 Kbp) from (i) the 522 human specific structural rearrangements; and, (ii) randomly picked segments with matching sizes within the same chromosome (100 random samplings for each rearrangement).
(PNG)

Figure S21 (A) Comparison of methylation levels of 100 Kbp windows obtained from sperm bisulfite sequencing data at 2.5× coverage and at 15× coverage (generated by Molaro et al. [35]). (B) Venn diagram of 100 Kbp windows with lowest 5% and 1% methylation levels at 15× (green circle) and 2.5× data (blue circle). The percentages represent proportions in the whole genome. The areas in elliptical-shadowed areas correspond to windows with lowest 1% methylation levels at 15× (green) and 2.5× (blue). The numbers in parenthesis (0.04% for lowest 5% and 0.43% for lowest 1%) are windows with valid methylation scores at 15× (>100CpG sampling events per 100 Kbp window) but invalid methylation scores at 2.5× coverage (<20CpG sampling events per 100 Kbp window).
(PNG)

Figure S22 CpG loci in methylation deserts have higher methylation variability across human tissues. (A) Heat map comparing actual methylation level of CpG loci in methylation deserts and randomly selected CpG loci from elsewhere across 11 tissues. (B) Kolmogorov-Smirnov tests comparing distribution of methylation level variation at assayed CpG loci across 11 types of human tissues (data from [57]): violet - CpG loci in methylation deserts; gray (dashed line) – CpG loci from elsewhere.
(PNG)

Figure S23 Extent of hypomethylation in the regions flanking CNVs from 270 HapMap samples determined using simulation test and sperm methylomes at 15× coverage. Dmax and significance values from KS tests show difference between the distribution of the methylation levels for 10 Kbp regions sampled at increasing distances (from 10 Kbp to 100 Kbp) from the CNVs and segments with matching sizes randomly picked from the same chromosomes (100 random samplings for each CNV).
(PNG)

Table S1 Genes located in the methylation deserts clustered by functional annotation using DAVID system [73]. The four clusters with highest enrichment scores and the three clusters with lowest enrichment scores are listed.
(PDF)

Table S2 Pairwise correlation coefficients among 7 methylomes determined using whole-genome bisulfite sequencing, and their correlation with the inferred MI values (the five somatic samples data are from previous publications [49], [50] and the two sperm methylomes determined at 2.5× joint coverage. The highest coefficients clustered the methylomes into different cell lineages, as highlighted with colors (light red - stem cell; light green - fibroblast; light blue - sperm).
(DOC)

Table S3 Methylation scores of windows containing CNVs detected in the two disease studies are significantly lower compared to the methylation scores in windows not containing the CNVs. The p-values are generated using Kolmogorov-Smirnov tests using the following two methylation scores: inferred germline methylation index values and sperm methylation scores determined using bisulphite sequencing at 2.5× coverage.
(DOC)

Table S4 Functional category clusters of genes within windows with MI = 0 determined by the DAVID system [73]. The three clusters with highest enrichment scores and the two clusters with lowest enrichment scores are listed.
(PDF)

Table S5 Clustering of genes located in the windows with MI = 0 by genetic disorder output by GFINDer [74] web server, sorted by p-values (without correction for multiple testing).
(PDF)

Table S6 Enrichment of various regulatory features in methylation deserts detected using permutation test or chi-square test. The highlighted rows also appear in **Table 2**.
(DOC)

Table S7 Comparing methylation levels at 15× coverage in evolutionary rearrangements or CNV segments vs. other genomic regions with two resolutions: 100 Kbp windows (2^nd^–3^rd^ columns); rearrangement/CNV segments vs. random segments of same size within the same chromosome (4^th^–5^th^ column).
(DOC)

Table S8 Chi-square test statistics for enrichment of various structural instabilities in the methylation deserts vs. the random windows with distances to the centromere/telomere selected from the normal distribution with the same parameters.
(DOC)

Table S9 Enrichment of structural mutability in methylation deserts of autosomal chromosomes and chromosome X (2.5× coverage). P-values are determined using Chi-square test.
(DOC)

Table S10 Accuracy of methylation level estimation. Based on the CpG coverage in each window, we calculated the binomial confidence interval for each window given the number of methylated CpG sampling events and the total number of CpG sampling events per window. Then we evaluated the relative error of the estimation of the methylation level for each window using the 95% confidence interval. This table shows the percentage of windows that do not exceed specific percentage error bounds. Joint read coverage of the two samples was 2.5×.
(DOC)

Table S11 Enrichment in hypomethylated regions (lowest 5% sperm methylation as determined by 2.5× coverage) of rare CNVs found in developmental delay patients classified by sub-phenotype (data from [41]). P-values are calculated using chi-square test, comparing case CNVs in each sub-class with all CNVs found in controls.
(DOC)

Text S1 Supplementary material for the main text.
(PDF)
