# Supplementary figures and images for: Genomic Hypomethylation in the Human Germline Associates with Selective Structural Mutability in the Human Genome
Source: PLoS Genet. 2012 May 17;8(5):e1002692. doi: 10.1371/journal.pgen.1002692 (PMC3355074; doi:10.1371/journal.pgen.1002692)

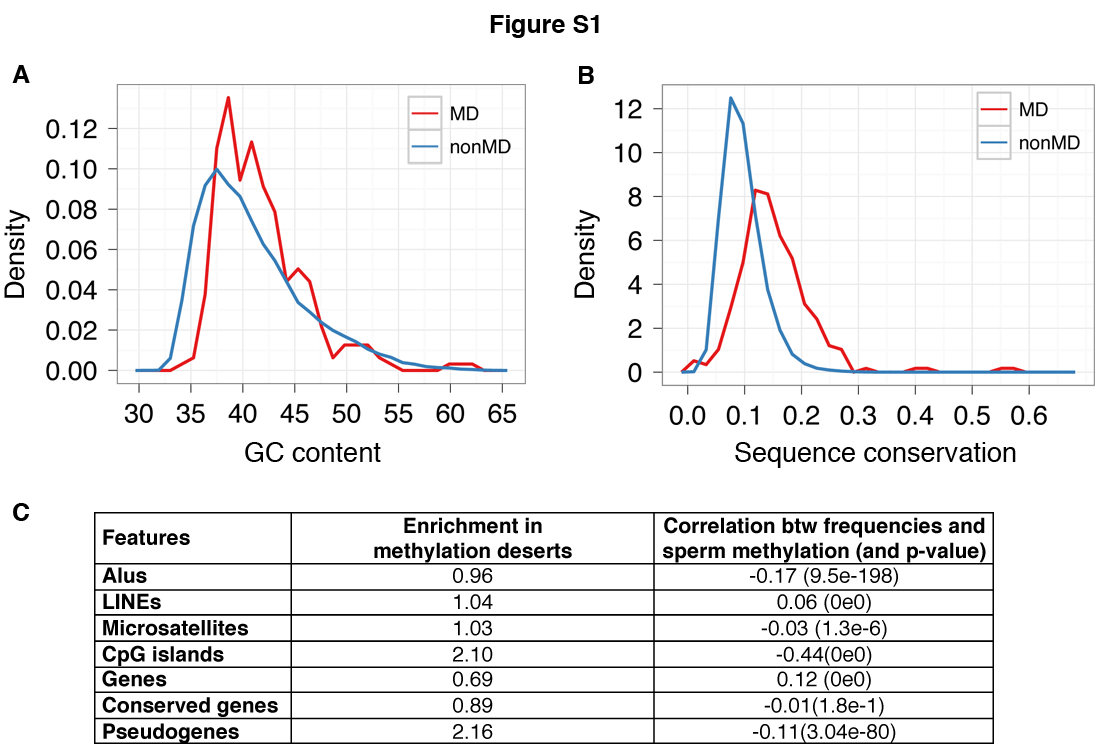

Supplement: Figure S1 — Comparison of genomic features in methylation deserts (MD, red) at 2.5× coverage and other regions with MI>0 (nonMD, blue) in the genome. Density plots of (A) GC content; and (B) sequence conservation. (C) Enrichment of various features in methylation deserts, and correlations between the features frequencies and sperm methylation levels across the 100 Kbp windows. (PNG) [file pgen.1002692.s001.png]

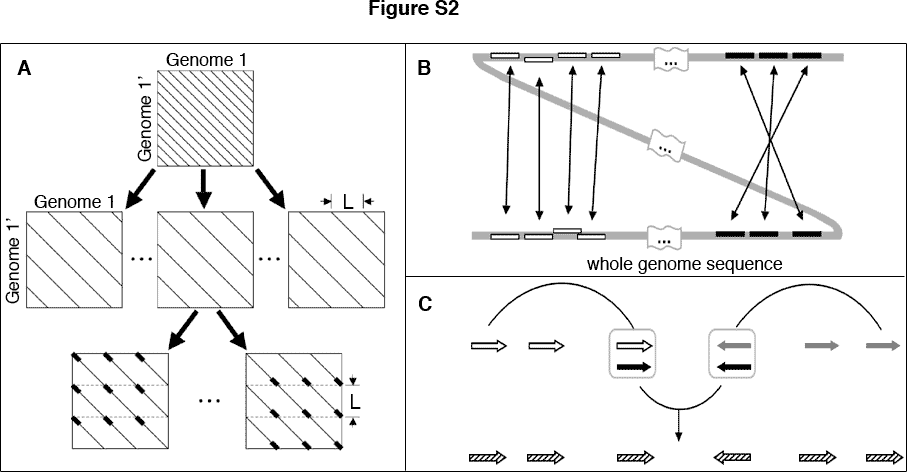

Supplement: Figure S2 — PASH pipeline for LCRs prediction. (A) PASH [67] divides the problem of whole genome comparison into groups of comparison diagonals (L-fragment length, set to 500 bp), which can be processed in parallel. For each group, each position along each diagonal is compared between the sequences sequentially using k-mers (k set to 13). (B) Reciprocal filtering select matching pairs of fragments identified in step A if they appear on each other's list of top 50 matches, then proximal fragments and their matching partners are merged into segments. (C) Identified pairwise LCRs from B were clustered into groups according k-mer content similarity and positional overlaps. (PNG) [file pgen.1002692.s002.png]

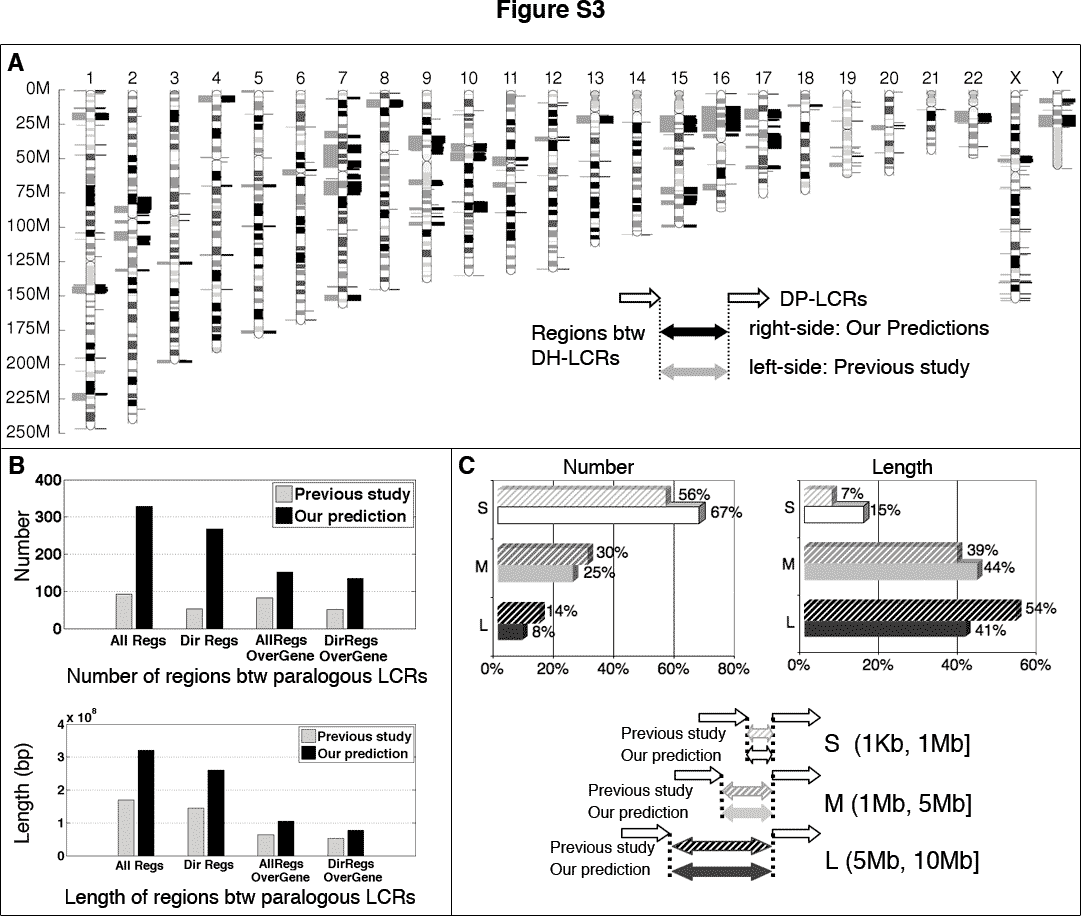

Supplement: Figure S3 — Comparison of regions between direct paralogous LCRs (DP-LCRs, length ≥10 Kbp, identity ≥95%, <10 Mbp apart) identified by our method and by Sharp et al. [6] (A) Locations of regions between DP-LCRs identified by our method (right-side of each chromosome ideogram), and those identified in the previous study (left-side). The heights of the bars indicate sizes of these regions. (B) Number and length coverage of the regions between paralogous LCRs identified by our method (black), compared with previous study (gray). (Four categories: (i) all regions between paralogous LCRs; (ii) regions between DP-LCRs; (iii) regions between paralogous LCRs and overlapping with genes; and (iv) regions between DP-LCRs and overlapping with genes). (C) Size distributions of regions between DP-LCRs identified by our method (solid color) compared with results from previous study (hatched color), in terms of number (left) and length (right). Small-(1 Kbp, 1 Mbp], white; Medium-(1 Mbp, 5 Mbp], gray; Large-(5 Mbp, 10 Mbp], black. (PNG) [file pgen.1002692.s003.png]

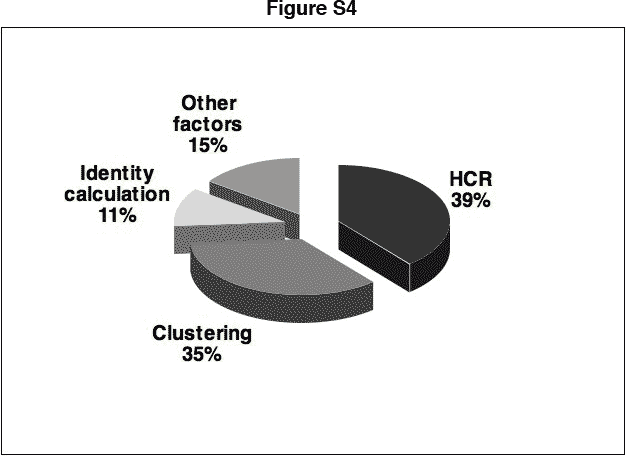

Supplement: Figure S4 — Factors contributing for increased detection of regions between DP-LCRs compared with previous study. 39% of the regions that we detect but are absent from the previous study occur between the newly identified LCRs that are enriched for HCRs. 35% of the novel regions occur between the newly clustered paralogous LCRs. 11% of the novel regions occur because of the different ways of calculating identity. 15% of the novel regions occur because of other factors, such as difference between genome builds on which the two studies were carried out. (PNG) [file pgen.1002692.s004.png]

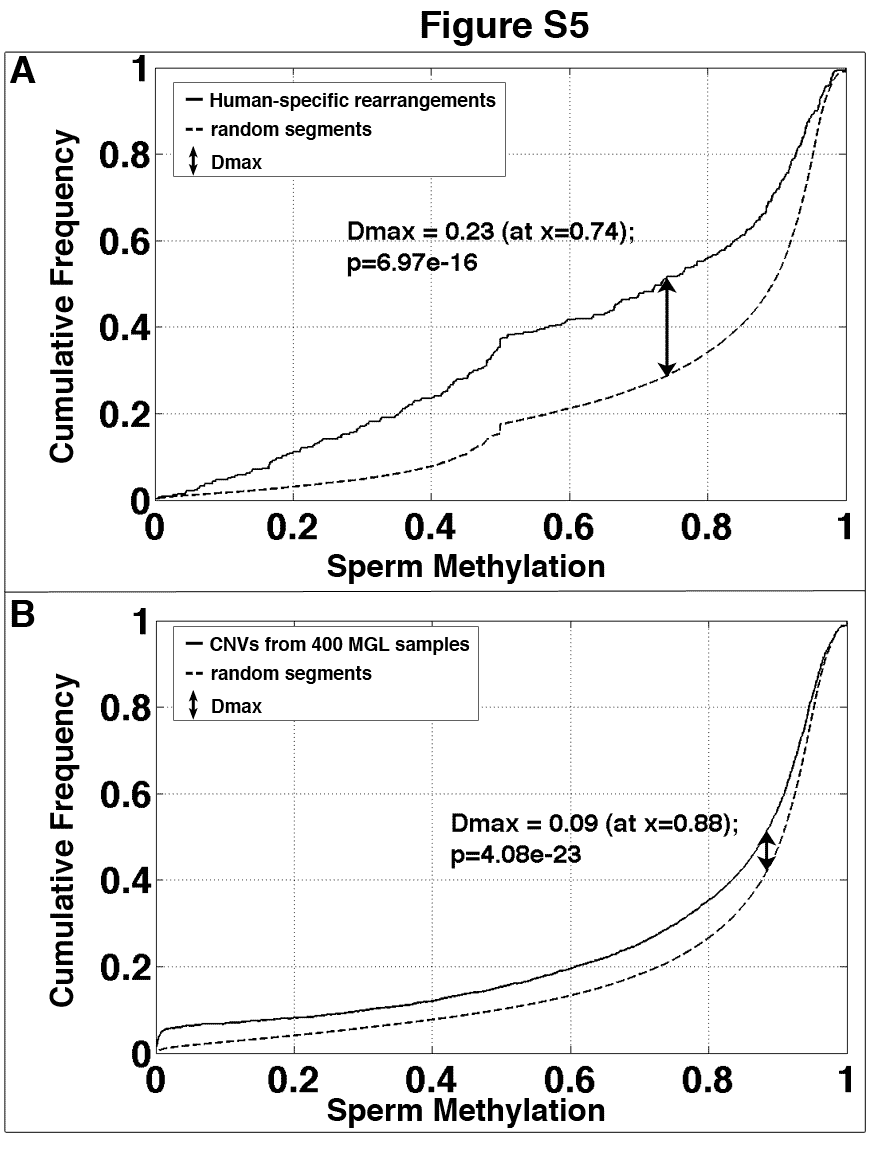

Supplement: Figure S5 — Permutation tests examining association between germline hypomethylation (at 2.5× coverage) and (A) human-specific structural rearrangements (B) CNVs detected in the 400 MGL samples. Kolmogorov-Smirnov (KS) tests comparing the distribution of the sperm methylation levels for the 522 human specific structural rearrangements in (A) and CNVs in (B) (solid lines) and the distribution obtained by randomly picking segments with matching sizes within the same chromosome (based on 100 random samplings for each evolutionary rearrangement or CNV) (dashed lines). The KS test statistic Dmax shows the greatest discrepancy between the two distributions. (PNG) [file pgen.1002692.s005.png]

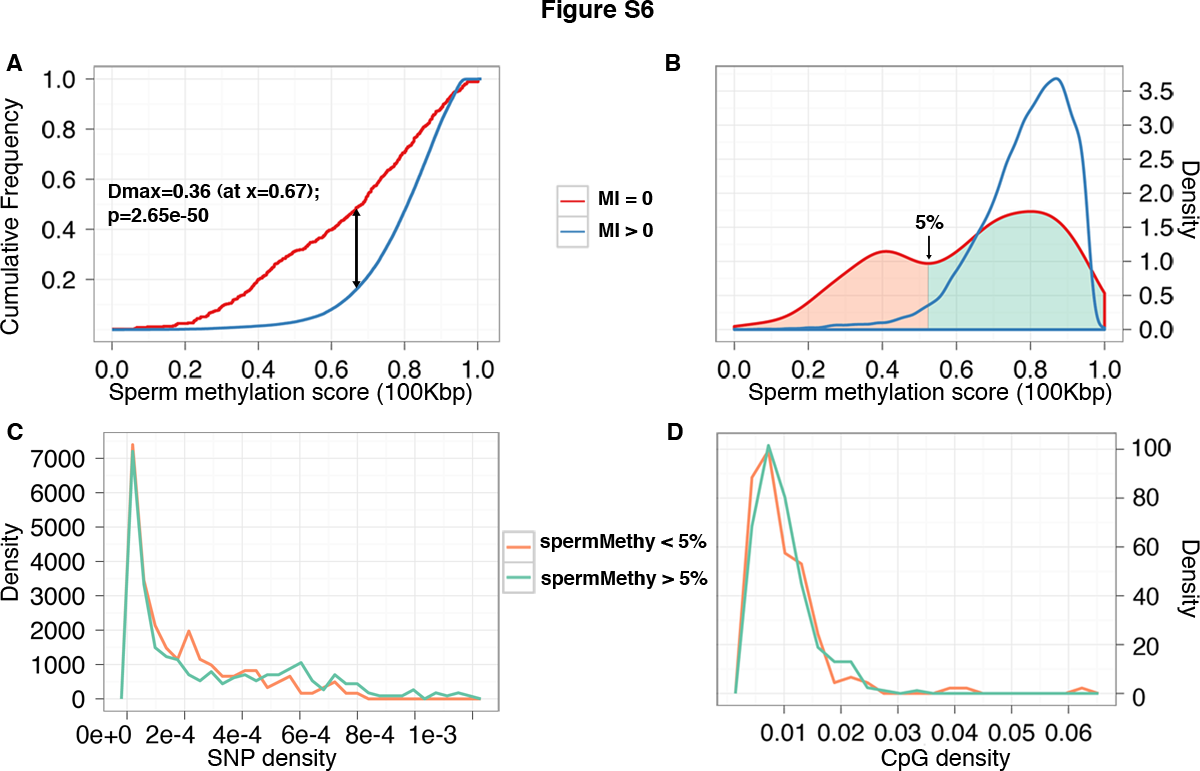

Supplement: Figure S6 — Sperm methylation levels (obtained by whole-genome methylation sequencing at 2.5× coverage) of 100 Kbp windows with methylation index MI = 0. (A) Cumulative distributions of sperm methylation levels for windows with MI = 0 (red) and the other windows (blue). The Kolmogorov-Smirnov (KS) statistic indicates significant difference between the two distributions. (B) Density plots of sperm methylation level for windows with MI = 0 (red) and the other windows (blue). The black arrow marks methylation level threshold separating the lower mode including ∼35% of the windows with MI = 0 (orange) and the higher mode including ∼65% of the windows with MI = 0 (green). (C–D) The two modes (indicated marked by orange and green lines matching respective orange and green areas under the two modes in (B)) have similar distribution of SNPs (C) and CpGs (D). (PNG) [file pgen.1002692.s006.png]

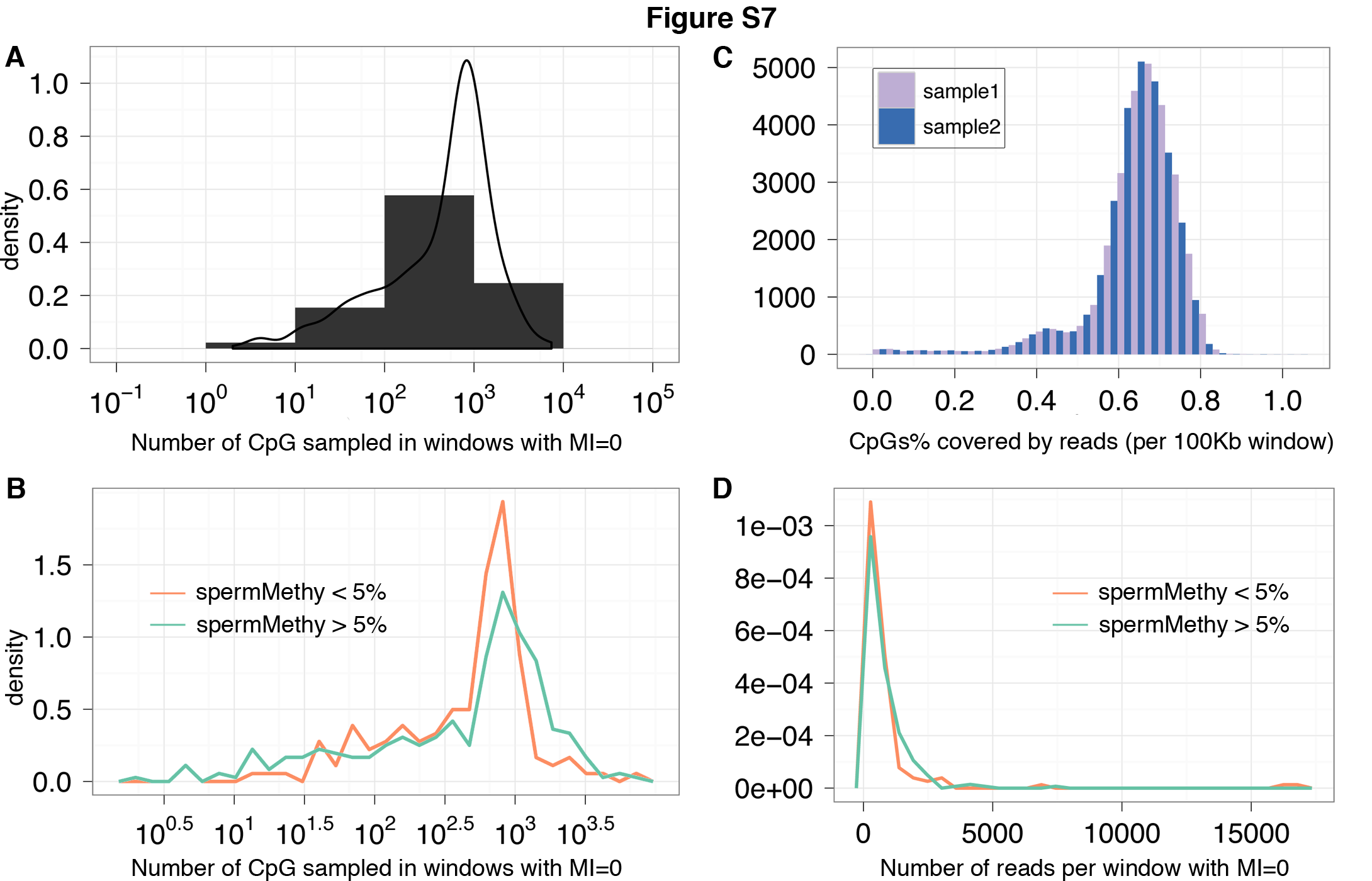

Supplement: Figure S7 — Density plots of the number of CpG dinucleotides sampled by bisulfite sequencing of sperm (at 2.5× coverage) in windows with MI = 0. (A) Histogram and density plots of CpG sampled in all windows with MI = 0. On average there are 787 CpG sampling events per window, with 95% of the MI = 0 windows having at least 20 CpG sampling events. (B) Density plots of number of CpG sampled per MI = 0 window. The two curves correspond to the two modes identified in Figure S6B are colored orange and green correspondingly. (C) Histogram plots for percentage of CpGs in each 100 Kbp window with at least 20 reads mapped from the two sperm samples being sequenced. (D) Density plots of the number of reads mapped in each 100 Kbp window with MI = 0. The two curves, colored orange and green, correspond to the two modes in Figure S6B and the two curves in (B). (PNG) [file pgen.1002692.s007.png]

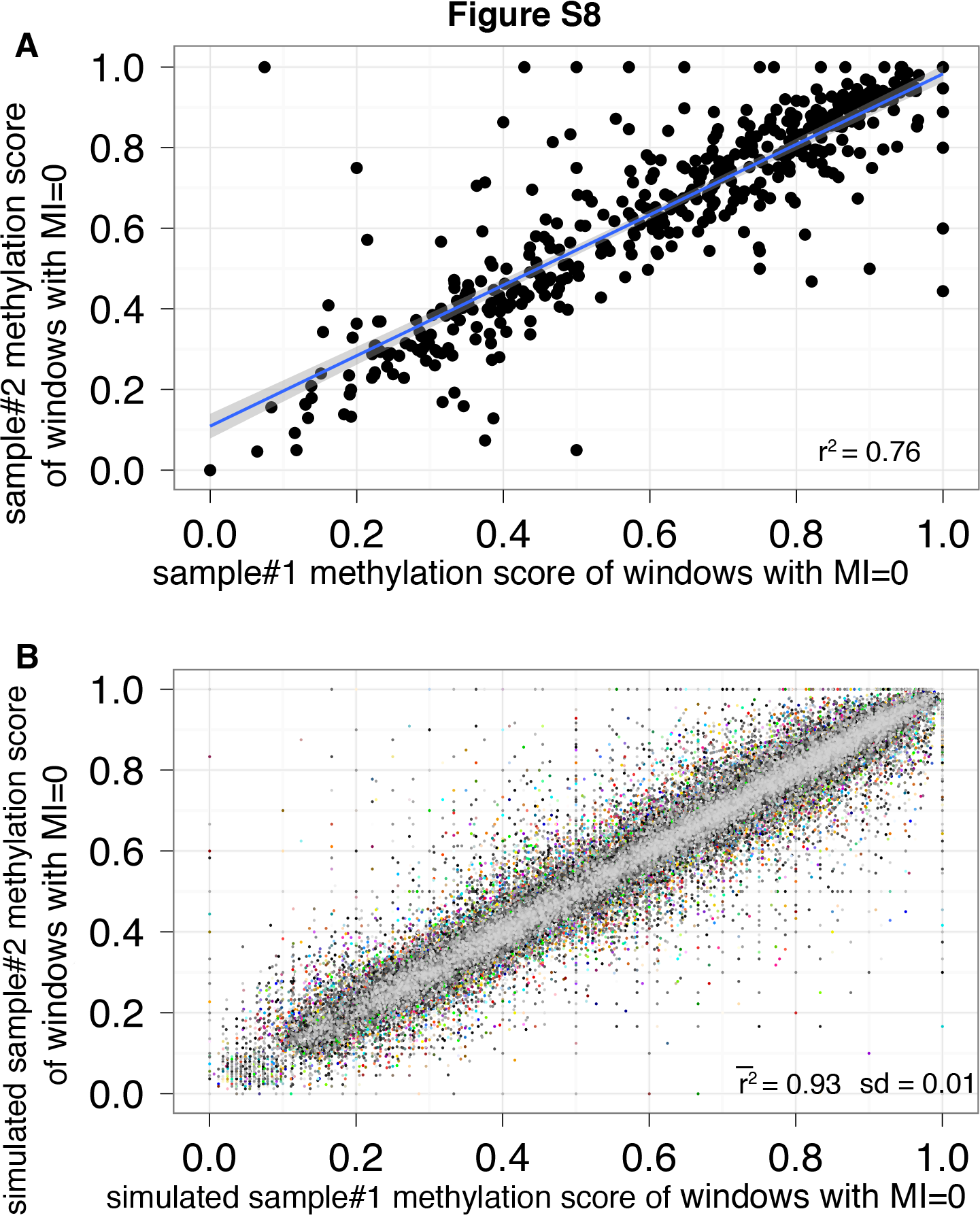

Supplement: Figure S8 — Scatter plots comparing true and simulated methylation scores of the two sperm samples (jointly covered at 2.5× read coverage) in 100 Kbp windows with MI = 0. (A) Linear regression of the actual scores from the two samples, with goodness of fit r2 = 0.76. (B) Results of a simulation experiment examining differences in methylation scores due to statistical variability assuming binomial sampling of CpGs, the statistical variation being a function of the number of CpG sampling events per window n and methylation levels p. The scatter plot indicates the results of 1000 iterations simulating the sampling process in windows with MI = 0 using binomial model B(n,p), where n is the number of CpG sampling events in each window and p is the probability of CpG being methylated in the same window. The averaged r2 for all simulations is 0.93, with a standard deviation 0.01. The combined evidence from (A) and (B) indicates that of the total variability between the two sperm samples (1−r2 = 1−0.76 = 0.24), less than one third (1−r2 = 1−0.93 = 0.07) is due to statistical variation. Inter-individual variation may accounts for a fraction of the residual variation (0.17). (PNG) [file pgen.1002692.s008.png]

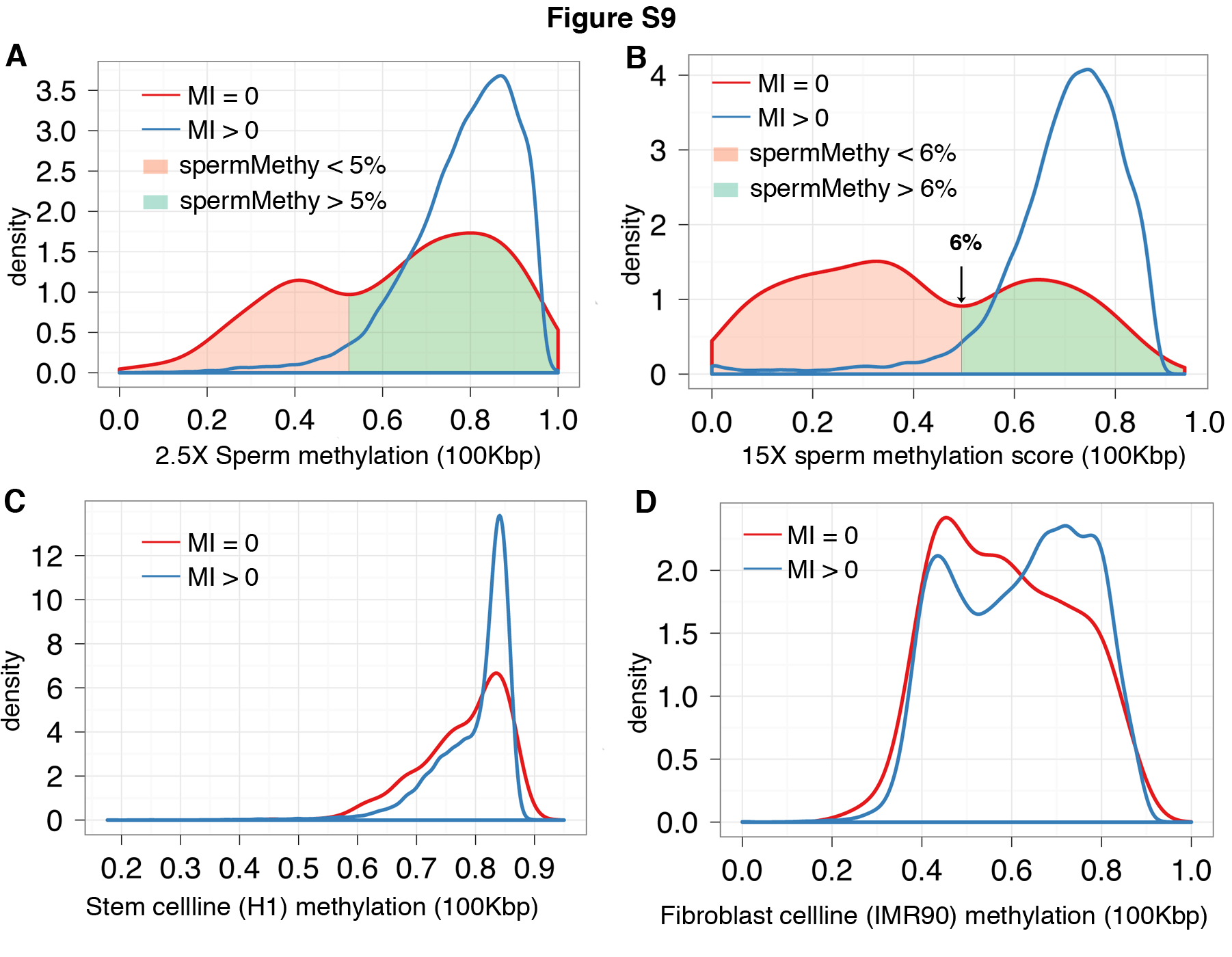

Supplement: Figure S9 — Comparison of methylation status in windows with MI = 0 (red) and other regions with MI>0 (blue) in sperm (A at 2.5× coverage and B at 15× coverage), embryonic stem cells (C), and fibroblasts (D). The left lower mode of the MI = 0 set is uniquely present in sperm, which is most closely related to human germline. (PNG) [file pgen.1002692.s009.png]

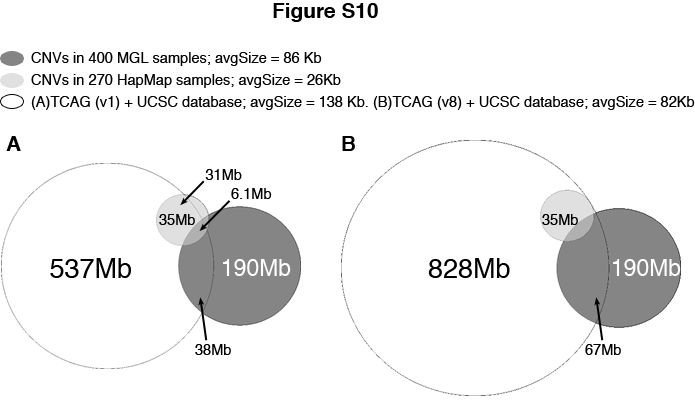

Supplement: Figure S10 — Venn diagram intersecting CNV loci identified from the 400 MGL samples using our custom Agilent array (dark gray), CNVs identified from the 270 HapMap samples using the Affymetrix SNP 6.0 array [36] (light gray), and CNVs from the TCAG database [70] (A) hg18.v1, the version that was available when the array was designed. (B) The same as (A) but with TCAG database version hg18.v8 and UCSC Structural Variation track (white). The numbers indicate total lengths of loci in basepairs. (PNG) [file pgen.1002692.s010.png]

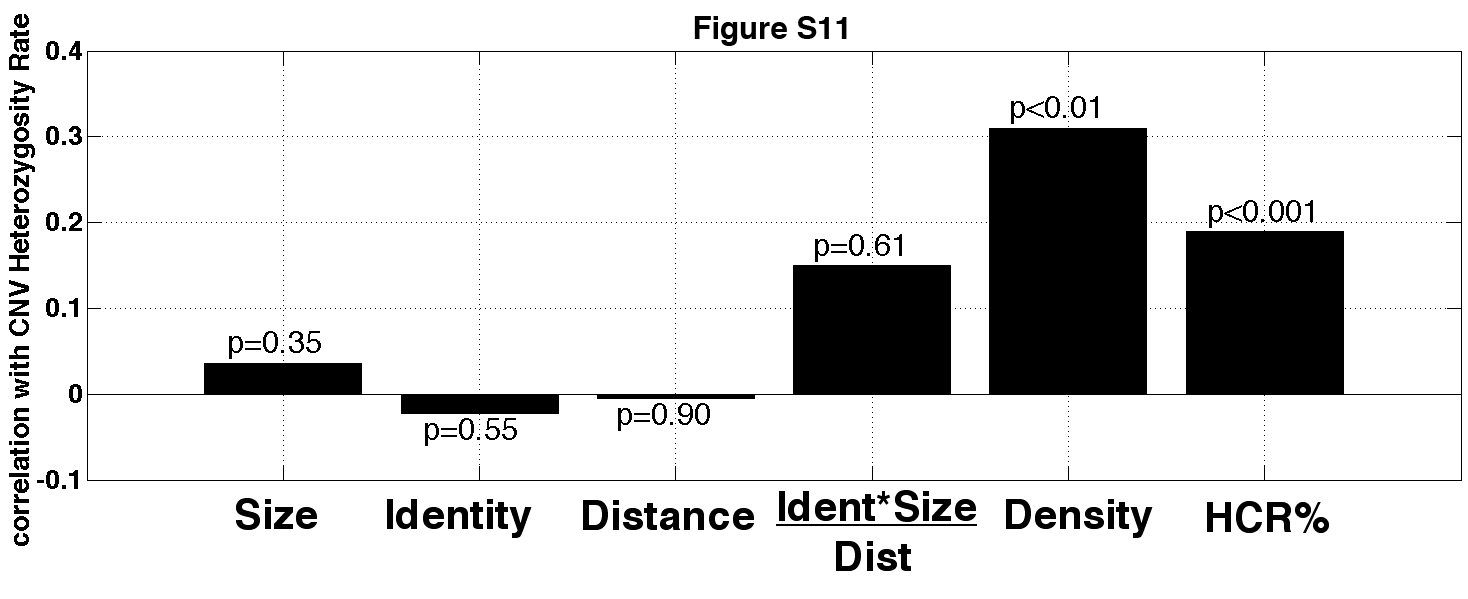

Supplement: Figure S11 — Correlation coefficients between structural heterozygosity rates and various properties of regions between paralogous LCRs: the size of the flanking paralogous LCRs, the sequence identity of the flanking paralogous LCRs, the distance between paralogous LCRs, a factor combining the previous three properties (Identity×Size/Distance), the density of surrounding LCRs, and the HCRs content of surrounding LCRs. (PNG) [file pgen.1002692.s011.png]

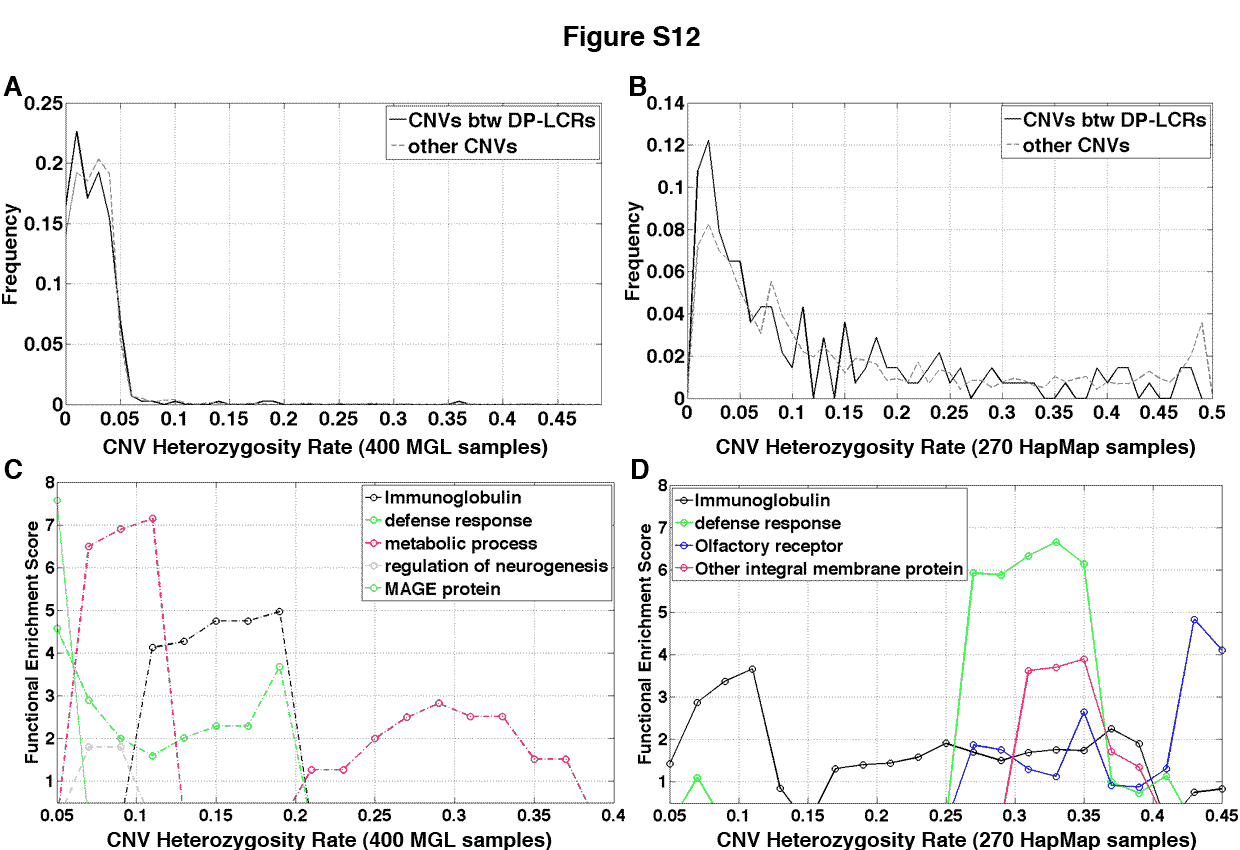

Supplement: Figure S12 — Distribution of structural heterozygosity rates and enrichment of functional gene annotations for CNVs detected in two datasets. (A–B) Distribution of structural heterozygosity rates for CNVs between DP-LCRs (solid line) and elsewhere (dashed line) in (A) 400 MGL samples and (B) 270 HapMap samples. (C–D) Functional gene annotation categories with highest enrichment scores at different CNV heterozygosity rates in (C) 400 MGL sample set and (D) 270 HapMap samples. (PNG) [file pgen.1002692.s012.png]

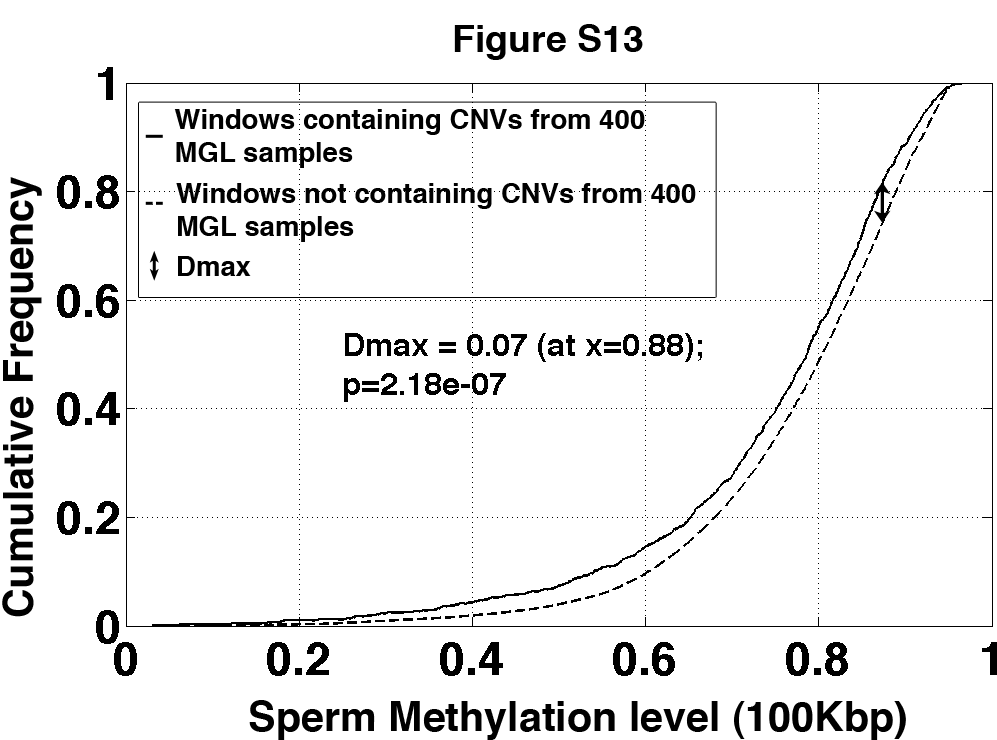

Supplement: Figure S13 — Association between germline hypomethylation (2.5× coverage) and structural polymorphism in the human population. Kolmogorov-Smirnov tests comparing sperm methylation levels distribution of 100 Kbp windows containing CNVs detected in the 400 MGL samples (solid line) and the rest of the windows (dashed line). (PNG) [file pgen.1002692.s013.png]

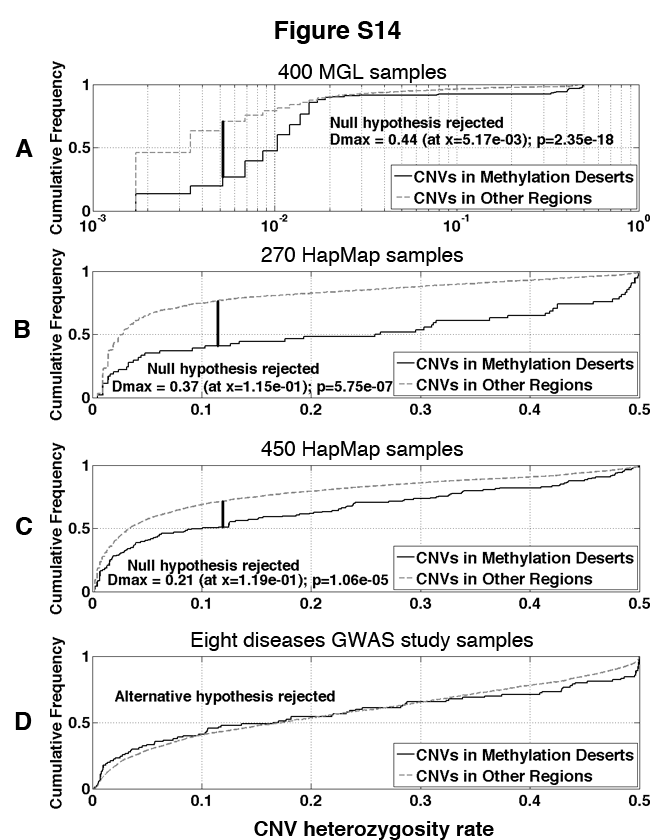

Supplement: Figure S14 — Kolmogorov-Smirnov tests comparing CNV heterozygosity rates in methylation deserts (2.5× coverage, 100 Kbp windows) and elsewhere in the genome for (A) 400 MGL samples; (B) 270 HapMap samples [36]; (C) 450 HapMap samples [37]; (D) 19,000 samples from eight common diseases GWAS study [38]. (PNG) [file pgen.1002692.s014.png]

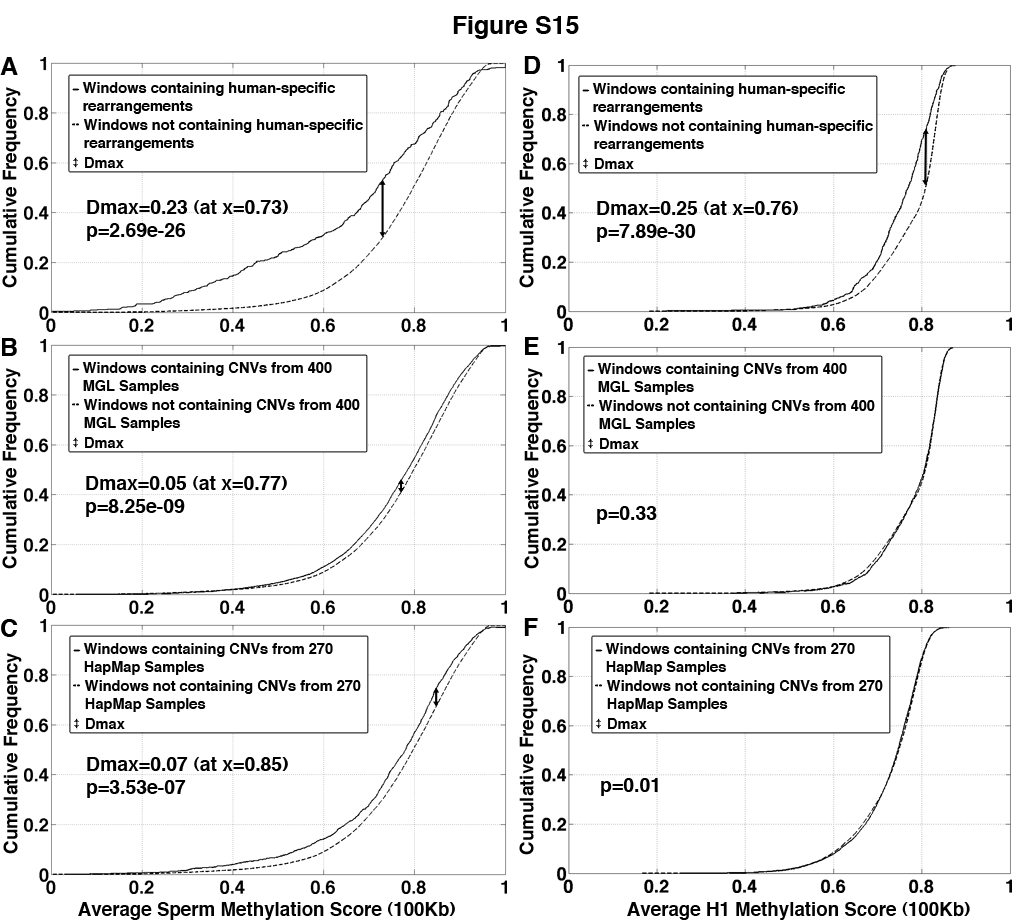

Supplement: Figure S15 — Association between structural variation and methylation in sperm (2.5× coverage) and H1 embryonic stem cells [50]. (A-sperm, D-H1): Kolmogorov-Smirnov tests comparing methylation score distribution of 100 Kbp windows containing human-specific structural rearrangements (solid line) and the rest of the windows (dashed line). (B-sperm, E-H1): Kolmogorov-Smirnov tests comparing methylation score distribution of 100 Kbp windows containing CNVs detected in the 400 MGL samples (solid line) and the rest of the windows (dashed line). (C-sperm, F-H1): Kolmogorov-Smirnov tests comparing methylation score distribution of 100 Kbp windows containing CNVs detected in the 270 HapMap samples (solid line) and the rest of the windows (dashed line). (PNG) [file pgen.1002692.s015.png]

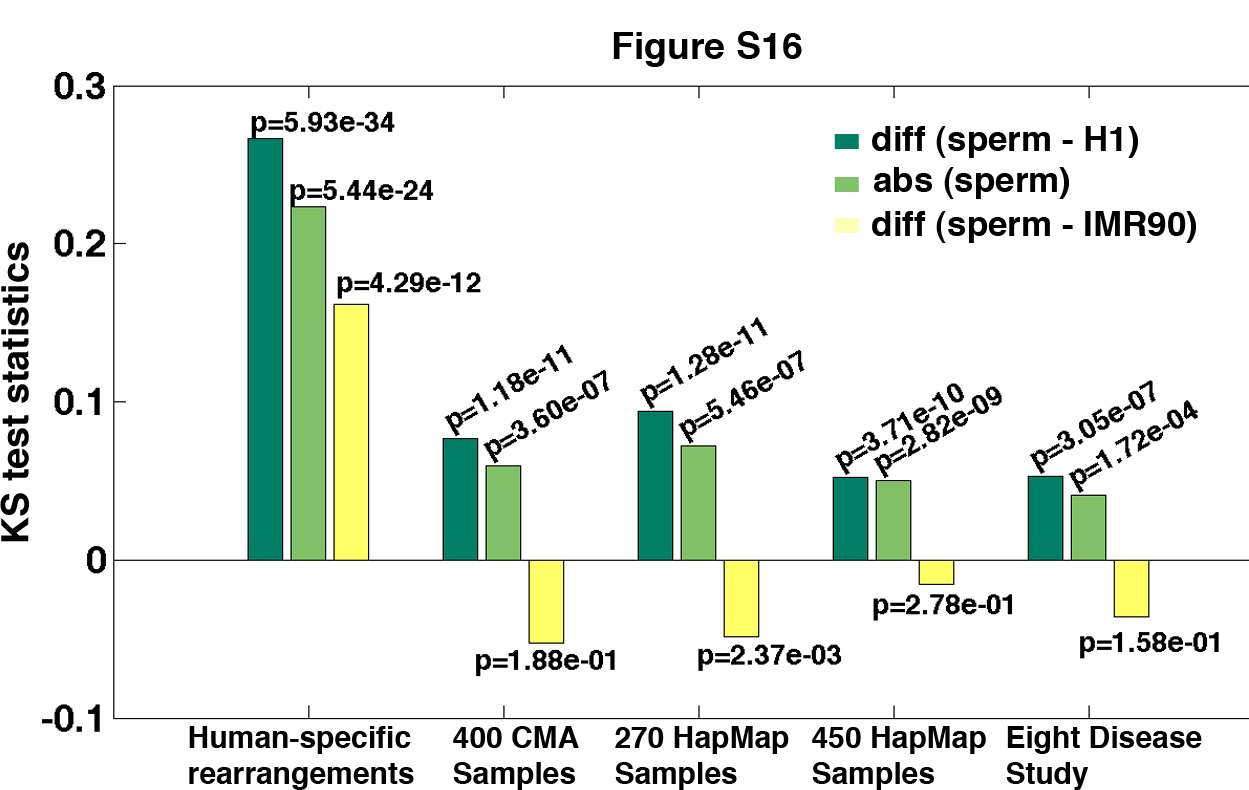

Supplement: Figure S16 — Kolmogorov-Smirnov (K-S) statistics obtained by comparing 100 Kbp windows containing structural variants and the rest of the windows. The 100 Kbp windows were assigned three different methylation scores: (1) methylation difference between sperm and H1 (dark green); (2) absolute methylation score in sperm at 2.5× coverage (light green); and (3) methylation difference between sperm (2.5×) and IMR90 (yellow). For all three type of scores, using K-S statistics we compared (i) the distribution of methylation level of 100 Kbp windows containing structural variants and (ii) the distribution of methylation scores of other windows. The bars with positive values indicate lower methylation scores in sperm. Specifically, windows containing structural variants show more negative methylation difference between sperm and H1 or between sperm and IMR90 (i.e. more hypomethylated in sperm), or smaller absolute sperm methylation scores (green bars). (PNG) [file pgen.1002692.s016.png]

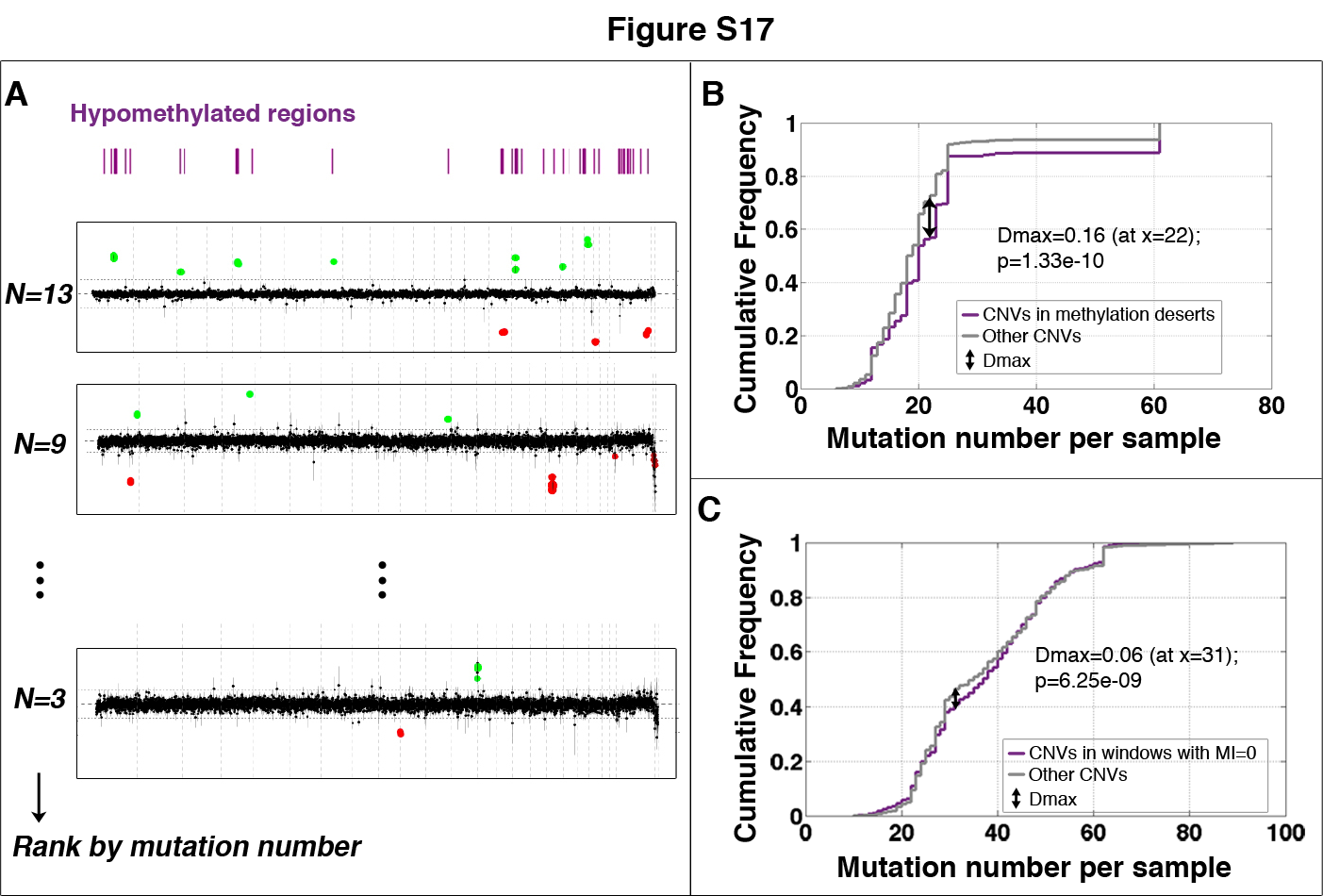

Supplement: Figure S17 — Increased concentration of CNVs from highly mutable samples in hypomethylated regions (2.5× coverage). (A) aCGH data are ranked by the total number of CNVs detected in each sample, as an indicator of mutability. (B) KS test comparing mutation number per sample in methylation deserts with lowest 1% sperm methylation level at 2.5× coverage (purple) vs. other regions (gray). (C) KS test comparing mutation number per sample in windows with MI = 0 (purple) vs. other regions (gray). (PNG) [file pgen.1002692.s017.png]

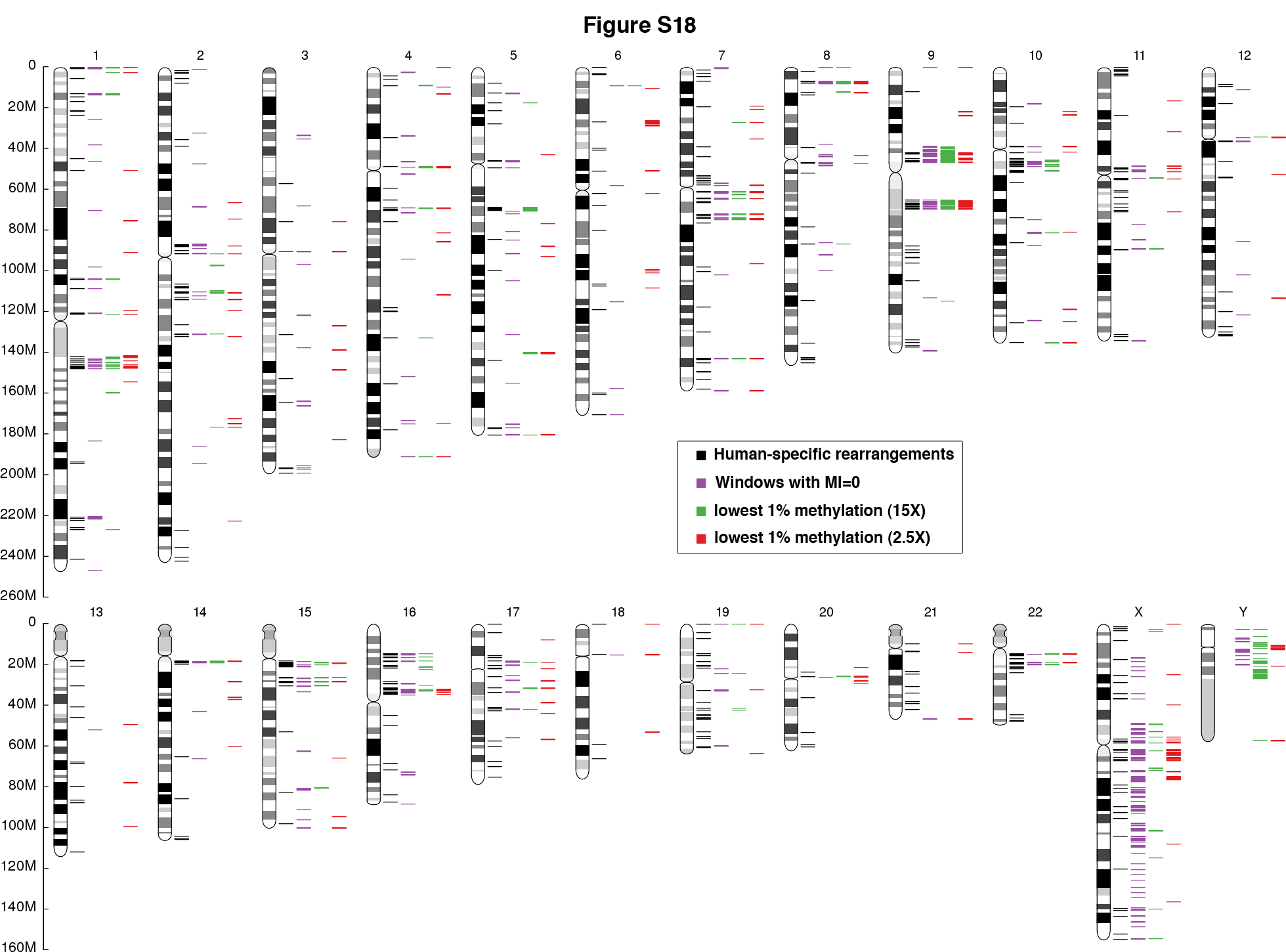

Supplement: Figure S18 — A whole genome visualization of the location of, human-specific structural rearrangements (black), windows with MI = 0 (violet), windows showing lowest 1% methylation in 15× data (green) and methylation deserts (windows showing lowest 1% methylation in our 2.5× data, (red). (PNG) [file pgen.1002692.s018.png]

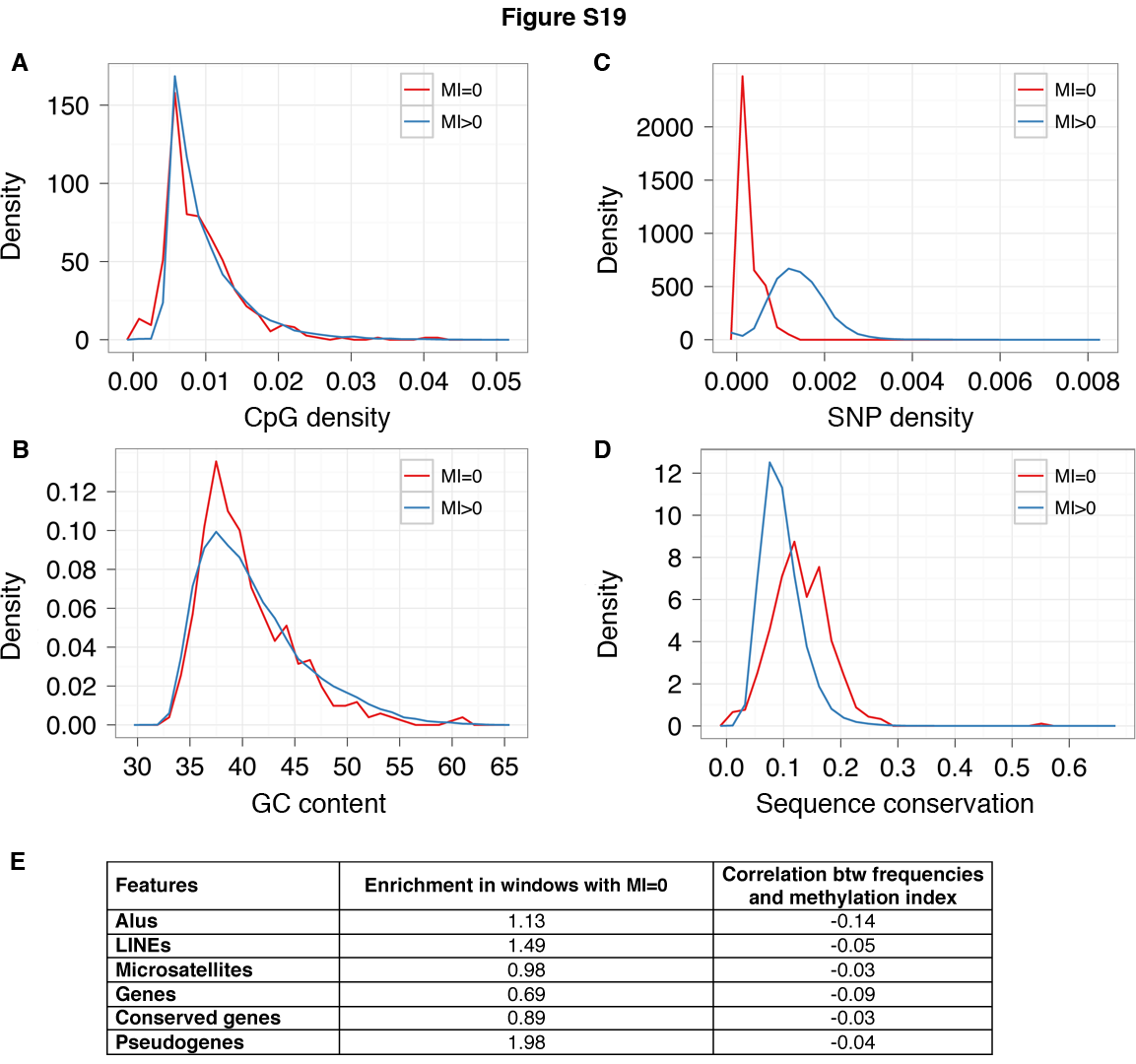

Supplement: Figure S19 — Comparison of genomic features in windows with MI = 0 (red) and other regions with MI>0 (blue) in the genome. Density plots of (A) CpG dinucleotide; (B) GC content; (C) SNP density; and (D) sequence conservation. (E) Enrichment of various features in windows with MI = 0, and correlations between the features frequencies and methylation index across the 100 Kbp windows. (PNG) [file pgen.1002692.s019.png]

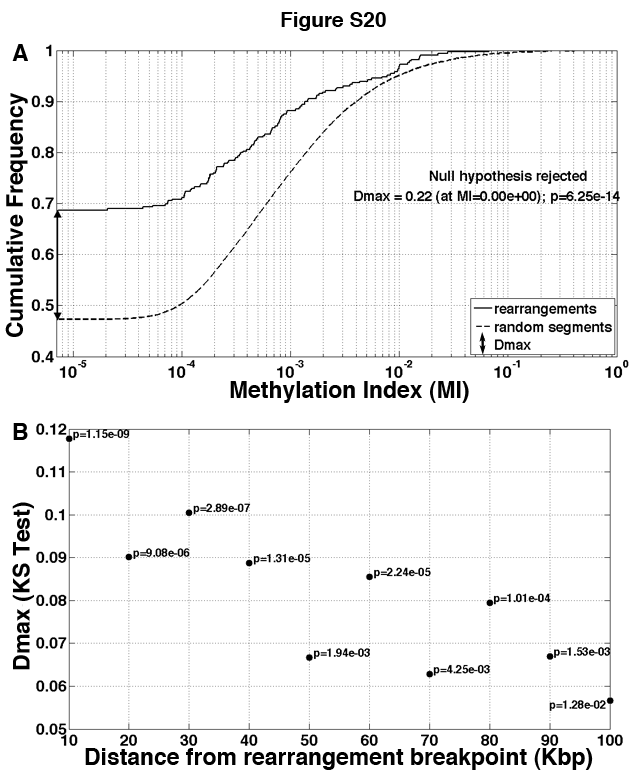

Supplement: Figure S20 — Permutation tests examining germline hypomethylation (measured by MI) within and around human-specific structural rearrangements. (A) Permutation testing of association between germline hypomethylation and human-specific structural rearrangements. Kolmogorov-Smirnov (KS) test comparing the distribution of the methylation index for (i) the 522 human specific structural rearrangements (solid line); and, (ii) randomly picked segments with matching sizes within the same chromosome (100 random samplings for each rearrangement) (dashed line). The KS test statistic Dmax shows the greatest discrepancy between the two distributions occurs at MI = 0. (B) Simulation test of extent of hypomethylation in the regions flanking human specific structural rearrangements. Dmax and significance values from KS tests show difference between the distribution of the methylation index for 10 Kbp regions sampled at increasing distances (from 10 Kbp to 100 Kbp) from (i) the 522 human specific structural rearrangements; and, (ii) randomly picked segments with matching sizes within the same chromosome (100 random samplings for each rearrangement). (PNG) [file pgen.1002692.s020.png]

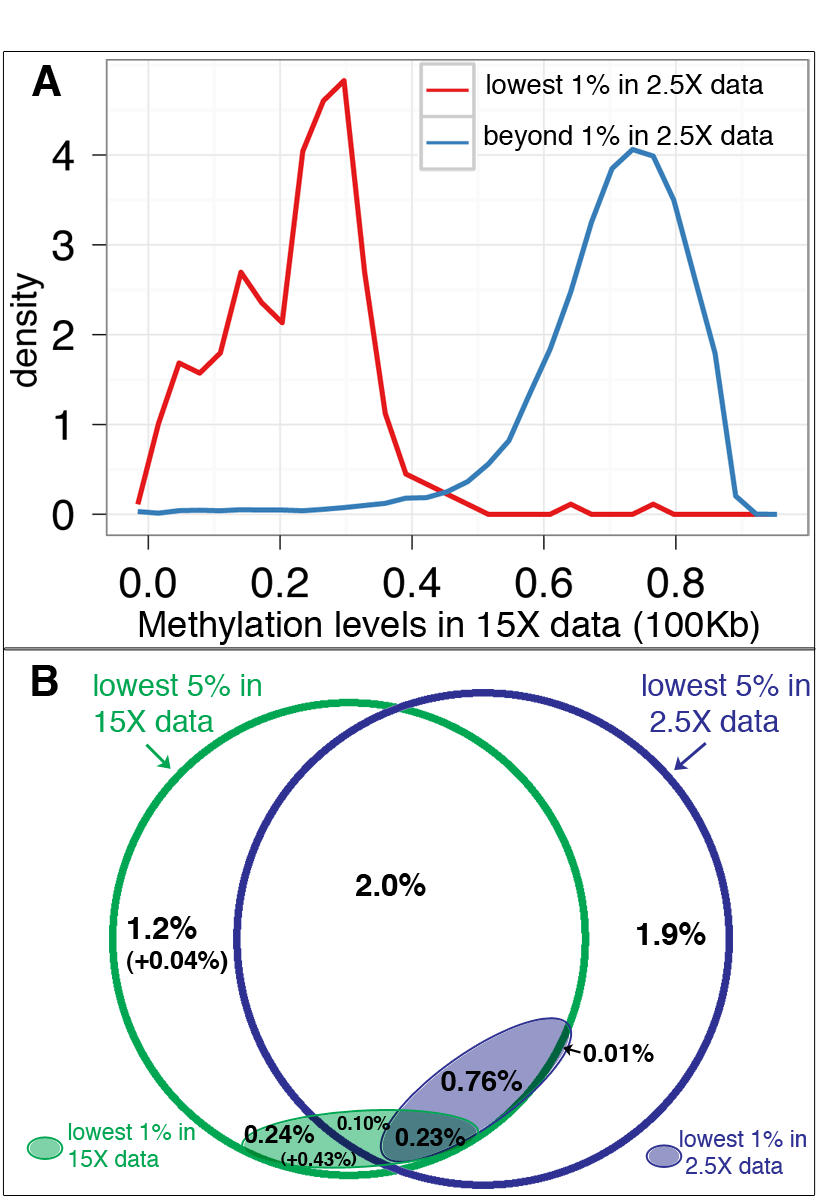

Supplement: Figure S21 — (A) Comparison of methylation levels of 100 Kbp windows obtained from sperm bisulfite sequencing data at 2.5× coverage and at 15× coverage (generated by Molaro et al. [35]). (B) Venn diagram of 100 Kbp windows with lowest 5% and 1% methylation levels at 15× (green circle) and 2.5× data (blue circle). The percentages represent proportions in the whole genome. The areas in elliptical-shadowed areas correspond to windows with lowest 1% methylation levels at 15× (green) and 2.5× (blue). The numbers in parenthesis (0.04% for lowest 5% and 0.43% for lowest 1%) are windows with valid methylation scores at 15× (>100CpG sampling events per 100 Kbp window) but invalid methylation scores at 2.5× coverage (<20CpG sampling events per 100 Kbp window). (PNG) [file pgen.1002692.s021.png]

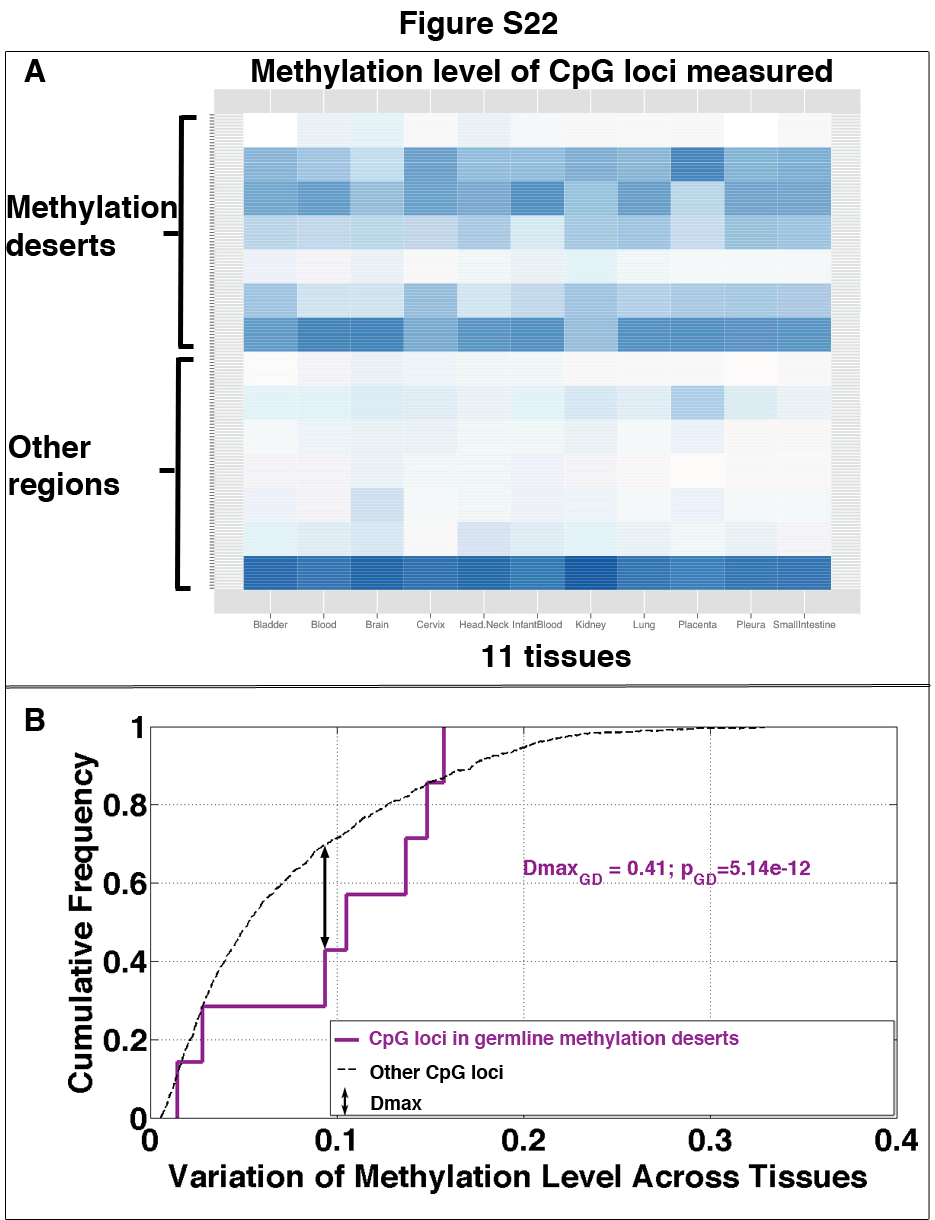

Supplement: Figure S22 — CpG loci in methylation deserts have higher methylation variability across human tissues. (A) Heat map comparing actual methylation level of CpG loci in methylation deserts and randomly selected CpG loci from elsewhere across 11 tissues. (B) Kolmogorov-Smirnov tests comparing distribution of methylation level variation at assayed CpG loci across 11 types of human tissues (data from [57]): violet - CpG loci in methylation deserts; gray (dashed line) – CpG loci from elsewhere. (PNG) [file pgen.1002692.s022.png]

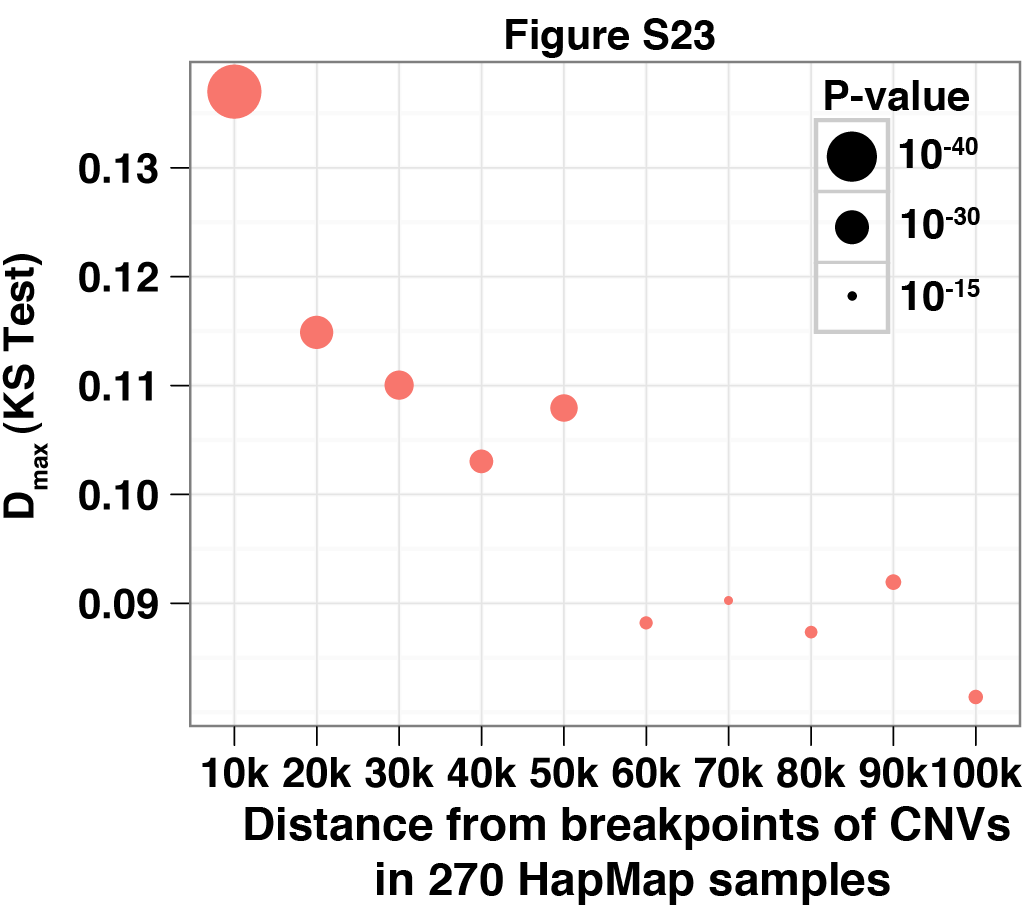

Supplement: Figure S23 — Extent of hypomethylation in the regions flanking CNVs from 270 HapMap samples determined using simulation test and sperm methylomes at 15× coverage. Dmax and significance values from KS tests show difference between the distribution of the methylation levels for 10 Kbp regions sampled at increasing distances (from 10 Kbp to 100 Kbp) from the CNVs and segments with matching sizes randomly picked from the same chromosomes (100 random samplings for each CNV). (PNG) [file pgen.1002692.s023.png]

Table S2


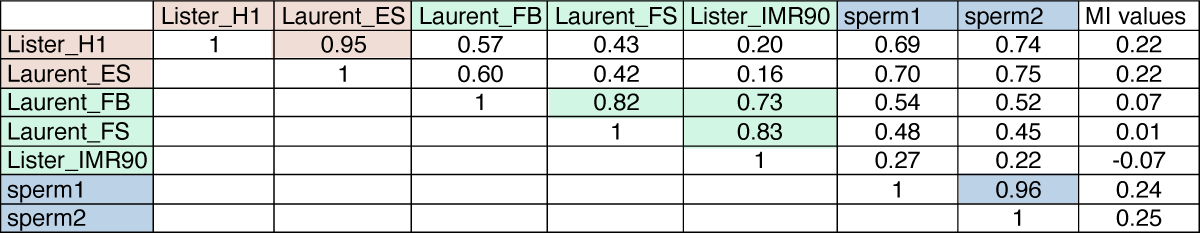

Supplement: Table S2 — Pairwise correlation coefficients among 7 methylomes determined using whole-genome bisulfite sequencing, and their correlation with the inferred MI values (the five somatic samples data are from previous publications [49], [50] and the two sperm methylomes determined at 2.5× joint coverage. The highest coefficients clustered the methylomes into different cell lineages, as highlighted with colors (light red - stem cell; light green - fibroblast; light blue - sperm). (DOC) [file pgen.1002692.s025.doc]
