# Supplementary material for: Genomic Hypomethylation in the Human Germline Associates with Selective Structural Mutability in the Human Genome
Source: PLoS Genet. 2012 May 17;8(5):e1002692. doi: 10.1371/journal.pgen.1002692 (PMC3355074; doi:10.1371/journal.pgen.1002692)
Supplement: Table S1 — Genes located in the methylation deserts clustered by functional annotation using DAVID system [73]. The four clusters with highest enrichment scores and the three clusters with lowest enrichment scores are listed. (PDF) [file pgen.1002692.s024.pdf]

Table S1

Annotation Cluster 1	Enrichment Score: 33.29							
Category	Term	Count	%	PValue	Fold Enrichment	Bonferroni	Benjamini	FDR
UP_SEQ_FEATURE	DNA-binding region:Homeobox	64	17.93	2.23E-67	21.75	1.52E-64	1.52E-64	3.35E-64
INTERPRO	IPR017970:Homeobox, conserved site	66	18.49	5.25E-64	18.02	1.33E-61	6.66E-62	6.83E-61
INTERPRO	IPR001356:Homeobox	66	18.49	1.34E-63	17.79	3.41E-61	1.14E-61	1.75E-60
SP_PIR_KEYWORDS	Homeobox	66	18.49	9.89E-63	17.60	1.94E-60	1.94E-60	1.24E-59
INTERPRO	IPR012287:Homeodomain-related	64	17.93	3.09E-60	17.03	7.85E-58	1.96E-58	4.03E-57
SMART	SM00389:HOX	66	18.49	3.82E-53	11.75	2.91E-51	2.91E-51	4.02E-50
SP_PIR_KEYWORDS	dna-binding	125	35.01	7.45E-49	4.32	1.46E-46	7.30E-47	9.31E-46
GOTERM_MF_FAT	GO:0043565~sequence-specific DNA binding	82	22.97	1.82E-48	7.31	3.42E-46	3.42E-46	2.26E-45
GOTERM_MF_FAT	GO:0003700~transcription factor activity	94	26.33	1.53E-43	5.22	2.87E-41	1.43E-41	1.89E-40
GOTERM_BP_FAT	GO:0007389~pattern specification process	52	14.57	1.47E-39	11.46	1.58E-36	7.91E-37	2.34E-36
SP_PIR_KEYWORDS	DNA binding	55	15.41	6.28E-39	10.44	1.23E-36	4.11E-37	7.85E-36
SP_PIR_KEYWORDS	developmental protein	75	21.01	8.83E-38	6.21	1.73E-35	4.33E-36	1.10E-34
GOTERM_MF_FAT	GO:0003677~DNA binding	131	36.69	1.14E-37	3.04	2.14E-35	7.15E-36	1.42E-34
GOTERM_BP_FAT	GO:0003002~regionalization	45	12.61	3.30E-37	13.44	3.56E-34	1.19E-34	5.27E-34
GOTERM_BP_FAT	GO:0048598~embryonic morphogenesis	50	14.01	4.35E-34	9.58	4.68E-31	1.17E-31	6.93E-31
GOTERM_MF_FAT	GO:0030528~transcription regulator activity	102	28.57	6.20E-34	3.65	1.16E-31	2.91E-32	7.69E-31
INTERPRO	IPR001827:Homeobox protein, antennapedia type, conserved site	21	5.88	4.13E-33	55.42	1.05E-30	1.75E-31	5.38E-30
GOTERM_BP_FAT	GO:0009952~anterior/posterior pattern formation	37	10.36	7.60E-33	15.54	8.18E-30	1.64E-30	1.21E-29
GOTERM_BP_FAT	GO:0006355~regulation of transcription, DNA-dependent	103	28.85	3.63E-32	3.42	3.91E-29	6.52E-30	5.79E-29
UP_SEQ_FEATURE	short sequence motif:Antp-type hexapeptide	20	5.60	1.78E-31	56.15	1.21E-28	2.02E-29	2.67E-28
GOTERM_BP_FAT	GO:0051252~regulation of RNA metabolic process	103	28.85	2.44E-31	3.34	2.63E-28	3.76E-29	3.90E-28
GOTERM_BP_FAT	GO:0048706~embryonic skeletal system development	29	8.12	1.74E-30	22.15	1.87E-27	2.08E-28	2.77E-27
GOTERM_BP_FAT	GO:0001501~skeletal system development	47	13.17	5.71E-30	8.67	6.15E-27	6.15E-28	9.11E-27
GOTERM_BP_FAT	GO:0048562~embryonic organ morphogenesis	32	8.96	6.24E-27	14.15	6.72E-24	6.11E-25	9.95E-24
SP_PIR_KEYWORDS	nucleus	147	41.18	7.48E-25	2.22	1.47E-22	2.45E-23	9.35E-22
SP_PIR_KEYWORDS	transcription regulation	96	26.89	2.68E-24	3.06	5.25E-22	7.50E-23	3.35E-21
GOTERM_BP_FAT	GO:0048705~skeletal system morphogenesis	28	7.84	5.51E-24	14.70	5.93E-21	4.94E-22	8.79E-21
SP_PIR_KEYWORDS	Transcription	96	26.89	1.33E-23	2.99	2.61E-21	3.27E-22	1.67E-20
GOTERM_BP_FAT	GO:0045449~regulation of transcription	111	31.09	2.10E-23	2.51	2.27E-20	1.74E-21	3.35E-20
GOTERM_BP_FAT	GO:0048568~embryonic organ development	32	8.96	2.53E-23	10.94	2.73E-20	1.95E-21	4.04E-20
GOTERM_BP_FAT	GO:0048704~embryonic skeletal system	22	6.16	3.04E-23	22.70	3.27E-20	2.18E-21	4.85E-20

	morphogenesis							
GOTERM_BP_FAT	GO:0006350~transcription	95	26.61	5.23E-21	2.66	5.64E-18	3.52E-19	8.35E-18
GOTERM_BP_FAT	GO:0009792~embryonic development ending in birth or egg hatching	38	10.64	4.12E-20	6.69	4.43E-17	2.61E-18	6.56E-17
GOTERM_BP_FAT	GO:0043009~chordate embryonic development	37	10.36	2.55E-19	6.57	2.75E-16	1.53E-17	4.07E-16
INTERPRO	IPR017995:Homeobox protein, antennapedia type	11	3.08	4.99E-17	58.06	1.27E-14	1.41E-15	6.50E-14
PIR_SUPERFAMILY	PIRSF002612:homeotic protein Hox A5/D4	10	2.80	6.40E-17	77.04	7.66E-15	7.66E-15	1.11E-13
PIR_SUPERFAMILY	PIRSF500606:homeotic protein Hox D4	5	1.40	1.27E-07	77.04	8.73E-06	2.91E-06	1.31E-04
SP_PIR_KEYWORDS	embryo	4	1.12	2.86E-04	28.69	0.054470354	0.00372704	0.356355857

**Annotation Cluster 2**	**Enrichment Score: 20.44**							
Category	Term	Count	%	PValue	Fold Enrichment	Bonferroni	Benjamini	FDR
INTERPRO	IPR013164:Cadherin, N-terminal	45	12.61	8.72E-69	49.14	2.21E-66	2.21E-66	1.14E-65
UP_SEQ_FEATURE	domain:Cadherin 6	44	12.32	9.99E-60	38.92	6.79E-57	3.40E-57	1.50E-56
UP_SEQ_FEATURE	domain:Cadherin 5	44	12.32	5.66E-53	29.91	3.85E-50	1.28E-50	8.51E-50
INTERPRO	IPR002126:Cadherin	45	12.61	1.74E-51	26.39	4.42E-49	8.84E-50	2.27E-48
UP_SEQ_FEATURE	domain:Cadherin 3	44	12.32	2.79E-51	27.85	1.90E-48	4.75E-49	4.19E-48
UP_SEQ_FEATURE	domain:Cadherin 4	44	12.32	2.79E-51	27.85	1.90E-48	4.75E-49	4.19E-48
UP_SEQ_FEATURE	domain:Cadherin 1	44	12.32	1.33E-50	27.06	9.04E-48	1.81E-48	2.00E-47
UP_SEQ_FEATURE	domain:Cadherin 2	44	12.32	1.33E-50	27.06	9.04E-48	1.81E-48	2.00E-47
GOTERM_BP_FAT	GO:0007156~homophilic cell adhesion	45	12.61	6.68E-46	20.20	7.20E-43	7.20E-43	1.07E-42
SMART	SM00112:CA	45	12.61	4.18E-44	17.43	3.18E-42	1.59E-42	4.40E-41
INTERPRO	IPR015492:Protocadherin gamma	19	5.32	3.60E-32	63.34	9.15E-30	1.31E-30	4.69E-29
GOTERM_BP_FAT	GO:0016337~cell-cell adhesion	45	12.61	1.47E-30	9.59	1.58E-27	1.98E-28	2.34E-27
SP_PIR_KEYWORDS	cell adhesion	46	12.89	6.38E-25	7.04	1.25E-22	2.50E-23	7.97E-22
GOTERM_BP_FAT	GO:0007155~cell adhesion	48	13.45	1.75E-16	4.03	2.39E-13	1.25E-14	3.55E-13
GOTERM_BP_FAT	GO:0022610~biological adhesion	48	13.45	1.85E-16	4.03	2.39E-13	1.20E-14	3.55E-13
SP_PIR_KEYWORDS	calcium	47	13.17	1.26E-14	3.78	2.48E-12	2.76E-13	1.58E-11
GOTERM_MF_FAT	GO:0005509~calcium ion binding	47	13.17	3.47E-10	2.77	6.52E-08	1.30E-08	4.30E-07
GOTERM_CC_FAT	GO:0005886~plasma membrane	65	18.21	9.94E-05	1.52	0.02	0.00	0.12
SP_PIR_KEYWORDS	cell membrane	53	14.85	0.00	1.56	0.19	0.01	1.35
GOTERM_CC_FAT	GO:0016021~integral to membrane	73	20.45	0.02	1.21	0.98	0.29	24.26
UP_SEQ_FEATURE	topological domain:Extracellular	54	15.13	0.04	1.28	1	0.67	47.14
GOTERM_CC_FAT	GO:0031224~intrinsic to membrane	73	20.45	0.05	1.17	1.00	0.45	46.68
UP_SEQ_FEATURE	topological domain:Cytoplasmic	58	16.25	0.25	1.11	1	0.99	98.54
GOTERM_MF_FAT	GO:0046872~metal ion binding	82	22.97	0.27	1.07	1	0.96	98.06

UP_SEQ_FEATURE	signal peptide	55	15.41	0.30	1.09	1	1.00	99.50
SP_PIR_KEYWORDS	signal	55	15.41	0.30	1.09	1	0.90	98.80
GOTERM_MF_FAT	GO:0043169~cation binding	82	22.97	0.31	1.06	1	0.97	98.94
GOTERM_MF_FAT	GO:0043167~ion binding	82	22.97	0.37	1.05	1	0.98	99.64
UP_SEQ_FEATURE	glycosylation site:N-linked (GlcNAc...)	58	16.25	0.85	0.91	1	1	100
SP_PIR_KEYWORDS	transmembrane	68	19.05	0.92	0.88	1	1.00	100
UP_SEQ_FEATURE	transmembrane region	67	18.77	0.92	0.88	1	1	100
SP_PIR_KEYWORDS	glycoprotein	58	16.25	0.93	0.87	1	1.00	100
SP_PIR_KEYWORDS	membrane	72	20.17	1.00	0.74	1	1	100

Annotation Cluster 3 **Enrichment Score: 12.57**

Category	Term	Count	%	PValue	Fold Enrichment	Bonferroni	Benjamini	FDR
GOTERM_BP_FAT	GO:0048736~appendage development	20	5.60	7.24E-15	11.42	7.77E-12	3.38E-13	1.15E-11
GOTERM_BP_FAT	GO:0060173~limb development	20	5.60	7.24E-15	11.42	7.77E-12	3.38E-13	1.15E-11
GOTERM_BP_FAT	GO:0009954~proximal/distal pattern formation	12	3.36	2.91E-14	30.69	3.13E-11	1.25E-12	4.64E-11
GOTERM_BP_FAT	GO:0035107~appendage morphogenesis	19	5.32	4.98E-14	11.29	5.36E-11	2.06E-12	7.93E-11
GOTERM_BP_FAT	GO:0035108~limb morphogenesis	19	5.32	4.98E-14	11.29	5.36E-11	2.06E-12	7.93E-11
GOTERM_BP_FAT	GO:0030326~embryonic limb morphogenesis	18	5.04	7.35E-14	12.17	7.92E-11	2.93E-12	1.17E-10
GOTERM_BP_FAT	GO:0035113~embryonic appendage morphogenesis	18	5.04	7.35E-14	12.17	7.92E-11	2.93E-12	1.17E-10
GOTERM_BP_FAT	GO:0035136~forelimb morphogenesis	7	1.96	1.31E-06	18.71	0.00141056	2.02E-05	0.00209032 1

Annotation Cluster 4 **Enrichment Score: 7.63**

Category	Term	Count	%	PValue	Fold Enrichment	Bonferroni	Benjamini	FDR
GOTERM_CC_FAT	GO:0000786~nucleosome	17	4.76	6.52E-18	23.79	1.06E-15	1.06E-15	7.89E-15
GOTERM_CC_FAT	GO:0032993~protein-DNA complex	17	4.76	1.47E-15	17.43	2.34E-13	1.17E-13	1.74E-12
INTERPRO	IPR007125:Histone core	14	3.92	1.25E-13	19.71	3.17E-11	3.17E-12	1.63E-10
SP_PIR_KEYWORDS	nucleosome core	14	3.92	2.59E-13	18.83	5.09E-11	5.09E-12	3.24E-10
GOTERM_BP_FAT	GO:0006334~nucleosome assembly	17	4.76	6.20E-13	11.90	6.68E-10	2.38E-11	9.89E-10
GOTERM_BP_FAT	GO:0031497~chromatin assembly	17	4.76	1.10E-12	11.49	1.18E-09	4.08E-11	1.75E-09
KEGG_PATHWAY	hsa05322:Systemic lupus erythematosus	14	3.92	2.11E-12	14.98	1.01E-10	1.01E-10	2.01E-09
GOTERM_BP_FAT	GO:0065004~protein-DNA complex assembly	17	4.76	2.28E-12	10.99	2.45E-09	8.17E-11	3.63E-09
GOTERM_BP_FAT	GO:0034728~nucleosome organization	17	4.76	3.23E-12	10.75	3.48E-09	1.09E-10	5.15E-09
GOTERM_CC_FAT	GO:0000785~chromatin	19	5.32	9.54E-12	8.37	1.55E-09	5.15E-10	1.15E-08
INTERPRO	IPR009072:Histone-fold	13	3.64	3.40E-11	15.25	8.63E-09	7.85E-10	4.43E-08
GOTERM_BP_FAT	GO:0006323~DNA packaging	17	4.76	1.19E-10	8.55	1.28E-07	3.78E-09	1.90E-07

PIR_SUPERFAMILY	PIRSF002050:histone H2B	8	2.24	2.73E-10	41.09	1.88E-08	9.42E-09	2.82E-07
GOTERM_BP_FAT	GO:0006333~chromatin assembly or disassembly	17	4.76	4.16E-10	7.87	4.48E-07	1.25E-08	6.64E-07
SP_PIR_KEYWORDS	chromosomal protein	17	4.76	9.01E-10	7.57	1.77E-07	1.61E-08	1.13E-06
INTERPRO	IPR000558:Histone H2B	8	2.24	6.03E-09	28.15	1.53E-06	1.18E-07	7.85E-06
GOTERM_CC_FAT	GO:0044427~chromosomal part	21	5.88	1.20E-08	4.80	1.94E-06	4.84E-07	1.45E-05
GOTERM_CC_FAT	GO:0005694~chromosome	22	6.16	4.50E-08	4.22	7.30E-06	1.22E-06	5.45E-05
SMART	SM00427:H2B	8	2.24	9.97E-08	18.59	7.58E-06	2.53E-06	1.05E-04
SP_PIR_KEYWORDS	isopeptide bond	19	5.32	2.56E-06	3.84	5.02E-04	4.18E-05	0.00
UP_SEQ_FEATURE	cross-link:Glycyl lysine isopeptide (Lys-Gly) (interchain with G-Cter in ubiquitin)	15	4.20	2.74E-06	4.87	0.00	1.55E-04	0.00
GOTERM_BP_FAT	GO:0034622~cellular macromolecular complex assembly	19	5.32	7.89E-06	3.51	0.01	1.05E-04	0.01
GOTERM_BP_FAT	GO:0034621~cellular macromolecular complex subunit organization	19	5.32	3.74E-05	3.13	0.04	4.68E-04	0.06
GOTERM_BP_FAT	GO:0006325~chromatin organization	19	5.32	7.84E-05	2.96	0.08	9.28E-04	0.12
SP_PIR_KEYWORDS	methylation	14	3.92	1.05E-04	3.73	0.02	0.00	0.13
SP_PIR_KEYWORDS	ubl conjugation	23	6.44	1.29E-04	2.52	0.02	0.00	0.16
SP_PIR_KEYWORDS	citrullination	5	1.40	3.27E-04	14.67	0.06	0.00	0.41
GOTERM_BP_FAT	GO:0051276~chromosome organization	20	5.60	5.98E-04	2.43	0.47	0.01	0.95
GOTERM_BP_FAT	GO:0065003~macromolecular complex assembly	21	5.88	0.01	1.86	1.00	0.07	13.73
GOTERM_BP_FAT	GO:0043933~macromolecular complex subunit organization	21	5.88	0.02	1.74	1.00	0.11	24.85
GOTERM_CC_FAT	GO:0043232~intracellular non-membrane-bounded organelle	38	10.64	0.07	1.29	1.00	0.51	57.50
GOTERM_CC_FAT	GO:0043228~non-membrane-bounded organelle	38	10.64	0.07	1.29	1.00	0.51	57.50
SP_PIR_KEYWORDS	acetylation	36	10.08	0.85	0.88	1.00	1.00	100

**Annotation Cluster 68**	**Enrichment Score: 0.0066**							
Category	Term	Count	%	PValue	Fold Enrichment	Bonferroni	Benjamini	FDR
GOTERM_BP_FAT	GO:0043066~negative regulation of apoptosis	3	0.84	0.984110227	0.50	1	0.999999669	100
GOTERM_BP_FAT	GO:0043069~negative regulation of programmed cell death	3	0.84	0.985262079	0.49	1	0.99999972	100
GOTERM_BP_FAT	GO:0060548~negative regulation of cell death	3	0.84	0.985482539	0.49	1	0.999999721	100

**Annotation Cluster 69**	**Enrichment Score: 0.0060**							
Category	Term	Count	%	PValue	Fold	Bonferroni	Benjamini	FDR

					Enrichment			
GOTERM_CC_FAT	GO:0005615~extracellular space	5	1.40	0.95393026	0.64	1	0.999999829	100
GOTERM_CC_FAT	GO:0044421~extracellular region part	5	1.40	0.995682095	0.46	1	1	100
GOTERM_CC_FAT	GO:0005576~extracellular region	13	3.64	0.997089406	0.57	1	1	100
GOTERM_BP_FAT	GO:0009611~response to wounding	3	0.84	0.998971915	0.33	1	1	100

Annotation Cluster 70 **Enrichment Score: 1.80E-7**

Category	Term	Count	%	PValue	Fold Enrichment	Bonferroni	Benjamini	FDR
UP_SEQ_FEATURE	nucleotide phosphate-binding region:ATP	3	0.84	0.999996381	0.20	1	1	100
SP_PIR_KEYWORDS	atp-binding	5	1.40	0.99999891	0.24	1	1	100
SP_PIR_KEYWORDS	nucleotide-binding	7	1.96	0.999999767	0.27	1	1	100
GOTERM_MF_FAT	GO:0005524~ATP binding	6	1.68	0.99999999	0.22	1	1	100
GOTERM_MF_FAT	GO:0032559~adenyl ribonucleotide binding	6	1.68	0.999999993	0.22	1	1	100
GOTERM_MF_FAT	GO:0032555~purine ribonucleotide binding	9	2.52	0.999999996	0.27	1	1	100
GOTERM_MF_FAT	GO:0032553~ribonucleotide binding	9	2.52	0.999999996	0.27	1	1	100
GOTERM_MF_FAT	GO:0030554~adenyl nucleotide binding	6	1.68	0.999999998	0.21	1	1	100
GOTERM_MF_FAT	GO:0001883~purine nucleoside binding	6	1.68	0.999999999	0.20	1	1	100
GOTERM_MF_FAT	GO:0017076~purine nucleotide binding	9	2.52	0.999999999	0.25	1	1	100
GOTERM_MF_FAT	GO:0001882~nucleoside binding	6	1.68	0.999999999	0.20	1	1	100
GOTERM_MF_FAT	GO:0000166~nucleotide binding	12	3.36	1	0.29	1	1	100
