# Supplementary material for: Genomic Hypomethylation in the Human Germline Associates with Selective Structural Mutability in the Human Genome
Source: PLoS Genet. 2012 May 17;8(5):e1002692. doi: 10.1371/journal.pgen.1002692 (PMC3355074; doi:10.1371/journal.pgen.1002692)
Supplement: Table S3 — Methylation scores of windows containing CNVs detected in the two disease studies are significantly lower compared to the methylation scores in windows not containing the CNVs. The p-values are generated using Kolmogorov-Smirnov tests using the following two methylation scores: inferred germline methylation index values and sperm methylation scores determined using bisulphite sequencing at 2.5× coverage. (DOC) [file pgen.1002692.s026.doc]

| **KS-test significance** | **Germline methylation index** | **Sperm**  **methylation level** |
| --- | --- | --- |
| **Schizophrenia case rare CNVs** | 5.0E-06 | 1.3E-03 |
| **Schizophrenia control rare CNVs** | 9.8E-01 | 4.1E-02 |
| **Autism case rare CNVs** | 1.0E-14 | 1.4E-06 |
| **Autism control rare CNVs** | 1.6E-07 | 5.6E-04 |

**Table S3**
