# Supplementary material for: Genomic Hypomethylation in the Human Germline Associates with Selective Structural Mutability in the Human Genome
Source: PLoS Genet. 2012 May 17;8(5):e1002692. doi: 10.1371/journal.pgen.1002692 (PMC3355074; doi:10.1371/journal.pgen.1002692)
Supplement: Table S4 — Functional category clusters of genes within windows with MI = 0 determined by the DAVID system [73]. The three clusters with highest enrichment scores and the two clusters with lowest enrichment scores are listed. (PDF) [file pgen.1002692.s027.pdf]

Table S4

Cluster 1 **Enrichment Score: 6.733353767876372**

Category	Term	Count	%	PValue	Fold Enrichment	Bonferroni	Benjamini	FDR
PIR_SUPERFAMILY	PIRSF038286:preferentially expressed antigen of melanoma (PRAME)	12	3.04	3.24E-15	35.52	3.19E-13	3.19E-13	3.56E-12
UP_SEQ_FEATURE	repeat:LRR 3	21	5.32	2.94E-07	4.04	2.64E-04	2.64E-04	4.58E-04
UP_SEQ_FEATURE	repeat:LRR 1	21	5.32	7.79E-07	3.80	6.99E-04	3.49E-04	0.00
UP_SEQ_FEATURE	repeat:LRR 2	21	5.32	8.18E-07	3.79	7.34E-04	2.45E-04	0.00
SP_PIR_KEYWORDS	leucine-rich repeat	21	5.32	9.01E-07	3.77	2.50E-04	2.50E-04	0.00
UP_SEQ_FEATURE	repeat:LRR 4	9	2.28	7.28E-02	2.06	1.00	0.99	69.18

Cluster 2 **Enrichment Score: 3.1089587443501245**

Category	Term	Count	%	PValue	Fold Enrichment	Bonferroni	Benjamini	FDR
INTERPRO	IPR011629:Cobalamin (vitamin B12) biosynthesis CobW-like, C-term	3	0.76	7.43E-04	62.86	0.28	0.28	1.05
INTERPRO	IPR003495:Cobalamin (vitamin B12) biosynthesis CobW-like	3	0.76	7.43E-04	62.86	0.28	0.28	1.05
PIR_SUPERFAMILY	PIRSF005738:conserved hypothetical protein YciC	3	0.76	7.49E-04	62.15	0.07	0.02	0.83
UP_SEQ_FEATURE	domain:CobW C-terminal	3	0.76	8.87E-04	57.57	0.55	0.15	1.37

Cluster 3 **Enrichment Score: 2.2738191636824197**

Category	Term	Count	%	PValue	Fold Enrichment	Bonferroni	Benjamini	FDR
SP_PIR_KEYWORDS	defensin	7	1.77	5.91E-05	10.11	0.02	0.01	0.08
GOTERM_BP_ALL	GO:0042742~defense response to bacterium	9	2.28	1.60E-04	5.79	0.19	0.19	0.26
GOTERM_BP_FAT	GO:0042742~defense response to bacterium	9	2.28	1.60E-04	5.78	0.17	0.17	0.26
INTERPRO	IPR001855:Beta defensin	4	1.01	1.00E-03	19.34	0.36	0.20	1.41
SP_PIR_KEYWORDS	antibiotic	7	1.77	1.13E-03	5.95	0.27	0.08	1.48
SP_PIR_KEYWORDS	Antimicrobial	7	1.77	1.41E-03	5.69	0.32	0.08	1.85
GOTERM_BP_FAT	GO:0009617~response to bacterium	10	2.53	1.46E-03	3.73	0.82	0.57	2.32
GOTERM_BP_ALL	GO:0009617~response to bacterium	10	2.53	1.47E-03	3.73	0.85	0.61	2.37
GOTERM_BP_ALL	GO:0051707~response to other organism	10	2.53	1.95E-02	2.48	1.00	0.99	27.51
GOTERM_BP_ALL	GO:0009607~response to biotic stimulus	10	2.53	8.59E-02	1.88	1.00	1.00	76.90
GOTERM_BP_ALL	GO:0006952~defense response	13	3.29	1.45E-01	1.52	1.00	1.00	92.19
GOTERM_BP_FAT	GO:0006952~defense response	13	3.29	1.45E-01	1.52	1.00	1.00	91.97
GOTERM_BP_ALL	GO:0006950~response to stress	29	7.34	1.73E-01	1.24	1.00	1.00	95.47
GOTERM_BP_ALL	GO:0051704~multi-organism process	11	2.78	4.68E-01	1.16	1.00	1.00	100.00

Cluster 102 **Enrichment Score: 7.900681949554787E-4**

Category	Term	Count	%	PValue	Fold Enrichment	Bonferroni	Benjamini	FDR
GOTERM_CC_FAT	GO:0031982~vesicle	4	1.01	9.97E-01	0.41	1.00	1.00	100.00
GOTERM_CC_FAT	GO:0016023~cytoplasmic membrane-bounded vesicle	3	0.76	9.98E-01	0.37	1.00	1.00	100.00
GOTERM_CC_ALL	GO:0031982~vesicle	4	1.01	9.98E-01	0.40	1.00	1.00	100.00
GOTERM_CC_ALL	GO:0016023~cytoplasmic membrane-bounded vesicle	3	0.76	9.98E-01	0.37	1.00	1.00	100.00
GOTERM_CC_FAT	GO:0031988~membrane-bounded vesicle	3	0.76	9.98E-01	0.36	1.00	1.00	100.00
GOTERM_CC_ALL	GO:0031988~membrane-bounded vesicle	3	0.76	9.98E-01	0.35	1.00	1.00	100.00
GOTERM_CC_FAT	GO:0031410~cytoplasmic vesicle	3	0.76	9.99E-01	0.32	1.00	1.00	100.00
GOTERM_CC_ALL	GO:0031410~cytoplasmic vesicle	3	0.76	9.99E-01	0.31	1.00	1.00	100.00

Cluster 103 **Enrichment Score: 2.2273332294124509E-4**

Category	Term	Count	%	PValue	Fold Enrichment	Bonferroni	Benjamini	FDR
GOTERM_BP_ALL	GO:0007155~cell adhesion	3	0.76	9.99E-01	0.31	1.00	1.00	100.00
GOTERM_BP_ALL	GO:0022610~biological adhesion	3	0.76	9.99E-01	0.31	1.00	1.00	100.00
GOTERM_BP_FAT	GO:0007155~cell adhesion	3	0.76	9.99E-01	0.31	1.00	1.00	100.00
GOTERM_BP_FAT	GO:0022610~biological adhesion	3	0.76	9.99E-01	0.31	1.00	1.00	100.00
