# Supplementary material for: Genomic Hypomethylation in the Human Germline Associates with Selective Structural Mutability in the Human Genome
Source: PLoS Genet. 2012 May 17;8(5):e1002692. doi: 10.1371/journal.pgen.1002692 (PMC3355074; doi:10.1371/journal.pgen.1002692)
Supplement: Table S5 — Clustering of genes located in the windows with MI = 0 by genetic disorder output by GFINDer [74] web server, sorted by p-values (without correction for multiple testing). (PDF) [file pgen.1002692.s028.pdf]

Table S5

			O: observed,E:expected, R: O/E ratio	
**Level**	**OMIM #**	**Category name**	**Statistics**	**P-value**
1	300157	Mental retardation	[O:4; E:0.53; R:7.55]	1.88E-03
1	300220	Chorioathetosis with mental retardation and abnormal behavior	[O:1; E:0.02; R:50]	2.44E-02
1	605194	Double-outlet right ventricle	[O:1; E:0.02; R:50]	2.44E-02
2	605194	Heterotaxy, visceral	[O:1; E:0.02; R:50]	2.44E-02
2	162200	Melanoma, desmoplastic neurotropic	[O:1; E:0.02; R:50]	2.44E-02
1	300032	Mental retardation-hypotonic facies syndrome	[O:1; E:0.02; R:50]	2.44E-02
2	300032	Mental retardation-hypotonic facies syndrome, X-linked	[O:1; E:0.02; R:50]	2.44E-02
1	609309	Muir-Torre syndrome	[O:1; E:0.02; R:50]	2.44E-02
1	607100	Nephronophthisis	[O:1; E:0.02; R:50]	2.44E-02
2	162200	Neurofibromatosis, familial spinal	[O:1; E:0.02; R:50]	2.44E-02
2	162200	Neurofibromatosis, type 1	[O:1; E:0.02; R:50]	2.44E-02
1	162200	Neurofibromatosis-Noonan syndrome	[O:1; E:0.02; R:50]	2.44E-02
1	162200	Pseudarthrosis	[O:1; E:0.02; R:50]	2.44E-02
2	162200	Pseudarthrosis, tibial	[O:1; E:0.02; R:50]	2.44E-02
3	162200	Pseudarthrosis, tibial, in NF1	[O:1; E:0.02; R:50]	2.44E-02
3	314310	Renal cell carcinoma, papillary, 1	[O:1; E:0.02; R:50]	2.44E-02
1	605194	Transposition of the great arteries	[O:1; E:0.02; R:50]	2.44E-02
1	162200	Watson syndrome	[O:1; E:0.02; R:50]	2.44E-02
2	300203	Epileptic encephalopathy, early infantile	[O:1; E:0.04; R:25]	3.63E-02
1	605194	Heterotaxy	[O:1; E:0.04; R:25]	3.63E-02
1	603930	Hyperekplexia	[O:1; E:0.04; R:25]	3.63E-02
1	601969	Medulloblastoma	[O:1; E:0.04; R:25]	3.63E-02
1	300560	Mental retardation syndrome	[O:1; E:0.04; R:25]	3.63E-02
2	300560	Mental retardation syndrome, X-linked	[O:1; E:0.04; R:25]	3.63E-02
2	300697	Mental retardation, X-linked syndromic	[O:1; E:0.04; R:25]	3.63E-02
1	603930	Molybdenum cofactor deficiency	[O:1; E:0.04; R:25]	3.63E-02
2	314310	Renal cell carcinoma, papillary	[O:1; E:0.04; R:25]	3.63E-02
1	300195	Alport syndrome	[O:1; E:0.05; R:20]	4.81E-02
1	300203	Epileptic encephalopathy	[O:1; E:0.05; R:20]	4.81E-02
2	162200	Leukemia, juvenile myelomonocytic	[O:1; E:0.05; R:20]	4.81E-02
