# Supplementary material for: Genomic Hypomethylation in the Human Germline Associates with Selective Structural Mutability in the Human Genome
Source: PLoS Genet. 2012 May 17;8(5):e1002692. doi: 10.1371/journal.pgen.1002692 (PMC3355074; doi:10.1371/journal.pgen.1002692)
Supplement: Table S6 — Enrichment of various regulatory features in methylation deserts detected using permutation test or chi-square test. The highlighted rows also appear in Table 2 . (DOC) [file pgen.1002692.s029.doc]

Table S6

| **Regulatory features** | **Fold-enrichment in**  **methylation deserts** | **p-value** |
| --- | --- | --- |
| Transcription factor coding sequence | 3.75 | < 1e-3 |
| **Two fast-evolving transcription factor clusters** | **15** | **< 1e-3** |
| Genomic regulatory blocks (GRB) | 2.8 | < 1e-10 |
| **GRB target genes vs. random segments** | **12** | **< 1e-10** |
| **GRB target genes vs. 'bystander' genes** | **9.2** | **1.42E-43** |
| **GRB target genes vs. other CpG island-overlapping genes outside GRBs** | **33** | **1.41E-146** |
| **Hyperconserved CpG domains with low COCAD scores** | **37.6** | **<1e-4** |
| Promoters with high CpG content | 1.1 | <1e-3 |
| Promoters with low CpG content | 0.65 | <1e-4 |
| Promoters with intermediate CpG content | 3.1 | <1e-4 |
| Bivalent promoters (H3K27me3 and H3K4me2) | 2.6 | <1e-3 |
| **Bivalent promoters with intermediate CpG content** | **4** | **<1e-3** |

Reference:

1. Vaquerizas JM, Kummerfeld SK, Teichmann SA, Luscombe NM (2009) A census of human transcription factors: function, expression and evolution. Nat Rev Genet 10: 252-263.

2. Akalin A, Fredman D, Arner E, Dong X, Bryne JC, et al. (2009) Transcriptional features of genomic regulatory blocks. Genome Biol 10: R38.

3. Tanay A, O'Donnell AH, Damelin M, Bestor TH (2007) Hyperconserved CpG domains underlie Polycomb-binding sites. Proc Natl Acad Sci U S A 104: 5521-5526.

4. Ku M, Koche RP, Rheinbay E, Mendenhall EM, Endoh M, et al. (2008) Genomewide analysis of PRC1 and PRC2 occupancy identifies two classes of bivalent domains. PLoS Genet 4: e1000242.
