# Supplementary material for: Genomic Hypomethylation in the Human Germline Associates with Selective Structural Mutability in the Human Genome
Source: PLoS Genet. 2012 May 17;8(5):e1002692. doi: 10.1371/journal.pgen.1002692 (PMC3355074; doi:10.1371/journal.pgen.1002692)
Supplement: Table S7 — Comparing methylation levels at 15× coverage in evolutionary rearrangements or CNV segments vs. other genomic regions with two resolutions: 100 Kbp windows (2nd–3rd columns); rearrangement/CNV segments vs. random segments of same size within the same chromosome (4th–5th column). (DOC) [file pgen.1002692.s030.doc]

Table S7

| **Structural Instabilities** | **100Kb windows containing structural instability vs. other windows** | | **Rearrangement/CNV segments vs. random segments** | |
| --- | --- | --- | --- | --- |
|  | **KS-test Dmax** | **p-value** | **KS-test Dmax** | **p-value** |
| **Human-specific rearrangements** | 0.39 | 8.72E-72 | 0.34 | 6.65E-52 |
| **270HapMap CNVs** | 0.13 | 6.08E-21 | 0.12 | 7.19E-19 |
| **400MGL CNVs** | 0.11 | 4.11E-20 | 0.1 | 5.80E-18 |
| **450HapMap CNVs** | 0.071 | 1.73E-18 | 0.1 | 2.60E-46 |
| **WTCCC CNVs** | 0.074 | 1.23E-13 | 0.13 | 3.67E-51 |
