# Supplementary material for: Genomic Hypomethylation in the Human Germline Associates with Selective Structural Mutability in the Human Genome
Source: PLoS Genet. 2012 May 17;8(5):e1002692. doi: 10.1371/journal.pgen.1002692 (PMC3355074; doi:10.1371/journal.pgen.1002692)
Supplement: Table S8 — Chi-square test statistics for enrichment of various structural instabilities in the methylation deserts vs. the random windows with distances to the centromere/telomere selected from the normal distribution with the same parameters. (DOC) [file pgen.1002692.s031.doc]

Table S8

| **methylation deserts vs. random windows** | **enrichment fold**  **(methylation deserts vs. control windows at similar distances to centromere/telomere)** | **chi-test**  **p-value** |
| --- | --- | --- |
| **human-specific rearrangement** | 9.56 | 6.38E-78 |
| **270HapMap** | 2.61 | 1.54E-08 |
| **450HapMap** | 1.51 | 0.01 |
| **WTCCC** | 1.89 | 2.39E-05 |
| **Schizophrenia case CNVs** | 1.55 | 3.50E-04 |
| **Autism case CNVs** | 2.16 | 6.54E-11 |
