# Supplementary material for: Genomic Hypomethylation in the Human Germline Associates with Selective Structural Mutability in the Human Genome
Source: PLoS Genet. 2012 May 17;8(5):e1002692. doi: 10.1371/journal.pgen.1002692 (PMC3355074; doi:10.1371/journal.pgen.1002692)
Supplement: Table S9 — Enrichment of structural mutability in methylation deserts of autosomal chromosomes and chromosome X (2.5× coverage). P-values are determined using Chi-square test. (DOC) [file pgen.1002692.s032.doc]

Table S9

| **Structural Instabilities** | **Autosomal chromosomes**  **fold-enrichment (p-value)** | **chrX**  **fold-enrichment (p-value)** |
| --- | --- | --- |
| **Human-specific rearrangements** | 9.4 (6.7e-74) | 3.9 (1.5e-2) |
| **270HapMap CNVs** | 2.3 (1.35e-05) | 2.1 (9.6e-1) |
| **450HapMap CNVs** | 1.3 (5.5e-2) | 1.24(8.2e-1) |
| **WTCCC CNVs** | 1.5 (5.8e-3) | 1.7(3.3e-1) |
| **400MGL CNVs** | 1.9 (2.1e-3) | 1.1(8.8e-1) |
| **Schizophrenia case-specific rare CNVs** | 2.0(7.6e-1) | no data |
| **Autism case *de novo* CNVs** | 3.8(4.2e-3) | 0.0(1.0) |
