# Supplementary material for: Genomic Hypomethylation in the Human Germline Associates with Selective Structural Mutability in the Human Genome
Source: PLoS Genet. 2012 May 17;8(5):e1002692. doi: 10.1371/journal.pgen.1002692 (PMC3355074; doi:10.1371/journal.pgen.1002692)
Supplement: Table S10 — Accuracy of methylation level estimation. Based on the CpG coverage in each window, we calculated the binomial confidence interval for each window given the number of methylated CpG sampling events and the total number of CpG sampling events per window. Then we evaluated the relative error of the estimation of the methylation level for each window using the 95% confidence interval. This table shows the percentage of windows that do not exceed specific percentage error bounds. Joint read coverage of the two samples was 2.5×. (DOC) [file pgen.1002692.s033.doc]

Table S10

| **Error bound** | **window%(sample#1)** | **window%(sample#2)** |
| --- | --- | --- |
| **1%** | 0.91% | 0.79% |
| **5%** | 91.62% | 92.16% |
| **10%** | 98.16% | 98.38% |
| **15%** | 99.08% | 99.18% |
| **20%** | 99.44% | 99.53% |
