# Supplementary material for: Genomic Hypomethylation in the Human Germline Associates with Selective Structural Mutability in the Human Genome
Source: PLoS Genet. 2012 May 17;8(5):e1002692. doi: 10.1371/journal.pgen.1002692 (PMC3355074; doi:10.1371/journal.pgen.1002692)
Supplement: Table S11 — Enrichment in hypomethylated regions (lowest 5% sperm methylation as determined by 2.5× coverage) of rare CNVs found in developmental delay patients classified by sub-phenotype (data from [41]). P-values are calculated using chi-square test, comparing case CNVs in each sub-class with all CNVs found in controls. (DOC) [file pgen.1002692.s034.doc]

Table S11

| **disease** | **fold enrichment** | **p-value** |
| --- | --- | --- |
| epilepsy | 2.7 | 1.1e-17 |
| autism | 2.1 | 2.4e-3 |
| cardiovascular | 2.5 | 7.76e-08 |
| Craniofacial defects | 3.2 | 1.34e-64 |
