# Supplementary material for: Genomic Hypomethylation in the Human Germline Associates with Selective Structural Mutability in the Human Genome
Source: PLoS Genet. 2012 May 17;8(5):e1002692. doi: 10.1371/journal.pgen.1002692 (PMC3355074; doi:10.1371/journal.pgen.1002692)
Supplement: Text S1 — Supplementary material for the main text. (PDF) [file pgen.1002692.s035.pdf]

Genomic hypomethylation in the human germline associates with structural mutability in the human genome

Jian Li, R. Alan Harris, Sau Wai Cheung, Cristian Coarfa, Mira Jeong, Margaret A. Goodell, Lisa D. White, Ankita Patel, Sung-Hae Kang, Chad Shaw, A. Craig Chinault, Tomasz Gambin, Anna Gambin, James R. Lupski, Aleksandar Milosavljevic

Supplementary Information

1. Identification of LCRs and comparison with predictions from previous studies

1.1 LCRs

The outcome of any evaluation of the association between structural variation and LCRs depends on an unbiased assessment of LCRs in the human genome. The segmental duplication track in the UCSC browser has been widely used as a reference LCR database. However, the Whole Genome Assembly Comparison (WGAC) method [1] used to predict this track started with a repeat-masking step, thus excluding high-copy-number repetitive elements (HCRs) such as SINEs and LINEs and causing under-detection of HCR-rich LCRs. The masking was computationally convenient but not strictly necessary: after retroposition, HCRs acquire uniqueness over evolutionary time by accumulating independent mutations. If the duplication of HCR-rich segments occurs after a certain amount of uniqueness is accumulated, the duplication can still be detected.

To avoid the potential under-ascertainment of LCRs due to HCR masking, we performed an independent assessment of LCRs. We particularly focused on a subset of them, directly-oriented paralogous LCRs, which we refer to in abbreviated form as DP-LCRs. DP-LCRs were hypothesized to be most likely to mediate deletions or duplications via NAHR, which can be detected by aCGH. The method we proposed for detecting LCRs and DP-LCRs consists of the following four steps: (1) represent sequences as lists of *k*-mers; (2) detect homology between LCRs; (3) cluster families of LCRs; and (4) detect and validate DP-LCRs (see Materials and Methods section 1 for details). The method uses *k*-mer frequency information to ignore *k*-mers that are overrepresented because of their presence in the highly repetitive elements while retaining the sensitivity required to detect HCR-rich LCRs.

Previous studies operationally defined LCRs as intra- and inter- chromosomal paralogous segments longer than 1Kbp sharing ≥90% sequence identity [1]. Other proposed operational definitions involve sequences of length 100-200bp, the size of an average exon [2]. By applying the same criteria as used in the WGAC method (>1Kbp length, 90% identity) to the human genome assembly build hg18, our method identified LCRs covering 6.15% (177.44Mbp) of the genome length (excluding the assembly gaps). Compared with the 5.5% as presented by the UCSC segDup track derived using the WGAC method with repeat masking, an 87.16% concordance is achieved. Investigation of the differences revealed that 22.79Mbp were new predictions that were not detected by WGAC and 6.32Mbp sequences were not covered by our method but by WGAC. 13.61Mbp of the 22.79Mbp newly detected LCRs are with high composition of HCRs (> 90%). Another 7.34Mbp of the 22.79Mbp newly detected LCRs may be explained by the sensitivity of our *k*-mer similarity search to detect homology. The remaining 1.84Mbp of newly detected LCRs may be due to the ambiguity of delineating the ends of LCR segments. A total of 6.32Mbp sequences not detected by our method but detected by the previous method may be either due to differences in similarity calculation or due to differences in delineation of LCR segment boundaries.

1.2 DP-LCRs (directly oriented paralogous LCRs)

The study of Sharp *et al.* [3] surveyed genomic loci flanked by paralogous LCRs with a goal of identifying loci susceptible to LCR-mediated deletions or duplications by the NAHR mechanism. It has been suggested that the paralogous LCRs with high similarity, large size, and in close proximity would be more likely to promote NAHR [3,4,5,6]. The study therefore considered only regions between paralogous LCRs with length ≥ 10Kbp, identity ≥ 95%, and inter-LCR distance <10Mbp. We applied the same criteria to independently derive a set of such LCRs (see Materials and Methods section 1 for details) and compared with the regions between such LCRs reported in the previous study.

Sharp *et al.* identified a total of 130 regions between LCRs with above criteria in human genome build hg16 [3]. To compare this prediction with the result of our method, we lifted over the 130 regions to the human build hg18, which resulted in a total of 93 valid regions. In contrast, our method identified 328 regions flanked by LCRs meeting the same criteria. The 328 regions included all the lifted over regions detected in the previous study and also the genomic disorder loci that are known to be associated with LCR-mediated NAHR (Prader Willi/Angelman syndromes, DiGeorge syndrome, etc., Figure S3A). 268 of these 328 regions are between DP-LCRs. Compared with the regions reported in the previous study, our predictions are more comprehensive not only in terms of the total number but also in terms of the total base-pair length (Figure S3B). By breaking down the predicted regions by their size we can show that our method detects higher proportion of small regions (Figure S3C).

There are multiple possible factors that contributed to the increased identification of the inter-paralogous-LCRs regions. To directly compare the difference between our predictions and Sharp *et al.*'s, we applied our method to human genome build hg16. The results showed that the increased sensitivity of prediction could largely be traced to sensitive detection of HCR-rich LCRs, and also to causes including the use of *k*-mer information to find paralogous clusters, sequence identity calculation and other factors such as the difference between genome builds (Figure S4).

2. Association of gene expression levels with methylation levels in germline

In the recent paper by Pacheco *et al.* [7], sperm mRNA from 18 men (with unknown fertility status and various semen characteristics) were hybridized onto the Affymetrix Human Gene 1.0 ST Array that offered whole-transcript coverage. Each of the 28,869 genes is represented on the array by approximately 26 probes spread across the full length of the gene, providing a more complete and more accurate picture of gene expression than 3' based expression array designs.

We downloaded the raw expression data of this study from GEO (accession# GSE26881), and the 18 array data were background-adjusted, normalized, and summarized using the RMA algorithm as implemented in the aroma.affymetrix software package [8].

We then found the transcripts located in each 100Kb windows, and averaged their expression values by windows (across all samples). We first performed two-sided Pearson correlation test on the methylation level and the average expression level of all 100Kb windows. However, no significant positive or negative correlation was found, suggesting no global correlation between the methylation and mRNA expression.

We next collected genes located in methylation deserts, and compared their expression levels (across all samples) with those located elsewhere. Genes in methylation deserts showed significantly elevated expression levels in sperm (KS-test, $D=0.05$, $p<2.2e-16$). Out of the 18 samples, 6 can be evaluated as normal using the following criteria: sperm count > 20 million, motility > 60%, morphology > 4% [9]. Using the data from these 6 samples we still observe a significantly elevated expression levels for transcripts in methylation deserts (KS-test, $D=0.05$, $p=4.4e-08$). Repeating the analysis for 15 out of the 18 samples individually, we could observe the similar patterns with significance value less than 0.05. The significance did not show specific correlation with the semen parameters.

3. Examination of possible ascertainment biases and confounding factors that may lead to the association between hypomethylation and the structural rearrangements/CNVs

Due to the presence of LCRs and other non-unique genomic regions, the association between hypomethylation and the structural rearrangements/CNVs may conceivably be explained away by ascertainment biases. First, since high association was detected between the structural rearrangements/CNVs and the LCRs, double counting of LCRs with similar methylation levels may conceivably create an ascertainment bias. To examine the possible extent of such bias, regions containing paralogous LCRs were combined together and counted only once when generating the methylation level distributions. Even under these conditions, the regions containing rearrangements/CNVs are still significantly less methylated than other regions. To correct for other possible factors resulting in non-uniqueness in the rearrangement/CNV regions, the *UMass uniqueness 15bp* track in UCSC database (http://genome.ucsc.edu/cgi-bin/hgTables?db=hg18&hgta_group=map&hgta_track=wgEncodeMapability&hgta_table=wgEncodeUmassMapabilityUniq15&hgta_doSchema=describe+table+schema) was used to quantify the level of sequence uniqueness. The average uniqueness value was calculated for each rearrangement/CNV region, and those with values less than one standard deviation from (smaller than) the genome-wide mean of the uniqueness values were eliminated from the KS-test. After this correction, the rearrangement/CNV regions still show significantly lower methylation level than other genomic regions.

We then examined possible confounding factors. Cytobands are known to correlate with the GC richness, repetitive element (*Alu*, L1) content, and chromatin state, which in turn may correlate with methylation state. We therefore assigned positive scores to the R bands (higher score for darker bands), negative scores to the G bands, and compared the distribution of the scores for windows containing the rearrangements/CNVs to the distribution of scores for random windows. The two distributions showed no significant difference (KS-test). If the cytobands were classified into only two types: positive and negative, then intersected with the rearrangements/CNVs, there were still no distributional difference detected (Chi-square test).

Since CpG island-associated regions tend to be unmethylated in human germline, we also examined whether the enrichment for CpG islands around the rearranged regions may explain the observed low methylation scores. Compared with the randomly selected regions, the rearranged regions did show slight enrichment (1.4 fold, permutation test, $p<10^{-3}$) for CpG islands (as defined in the UCSC database). However, when the permutation test (comparing methylation level of rearrangements/CNVs vs. random segments) was repeated by correcting for the CpG islands (removing anything intersecting CpG islands), significantly lower methylation scores can still be observed to be associated with the rearrangements/CNVs (KS-test). This indicates that the presence of CpG islands

cannot explain the low methylation score for the rearrangements/CNVs; on the other hand, the loci containing rearrangements/CNVs may indeed have escaped CpG depletion because of their unmethylated state in the germ line.

Next, we examined whether proximity to telomeres or centromeres may explain observed hypomethylation patterns. By permutation test, we do observe methylation deserts are highly enriched around the centromeric regions (22 fold enrichment of regions within 100Kbp of centromeres), and also around the telomeric regions (6 fold). To test whether such enrichment explains away the correlation between hypomethylation and structural instability, we further asked if correlation between hypomethylation and structural instability would be detected if the control segments had the same distribution of distances from centromeric/telomeric regions as the methylation deserts. To answer this question, we first collected the methylation deserts of each chromosome and found their distances to the closest centromere/telomere; we then calculated the mean and variance of these distances; finally, we generated windows with random distances sampled from a normal distribution with the mean and std relative to the same centromere/telomere. (If the original window was closest to the centromere, the random one would be forced to be on the opposite side of the centromere). We repeated the simulation 100 times. Using chi-square test we calculated the enrichments of all the types of structural mutabilities in the methylation deserts vs. the randomly selected control windows. As shown in Table S8, we can still observe the methylation deserts are significantly enriched with all types of structural instabilities, with the exception of the 450 HapMap CNVs, which showed less enrichment.

We also asked whether the association between hypomethylation and structural instabilities showed sex chromosomes bias. Out of all the categories of structural mutability data we examined (human specific rearrangement, structural polymorphisms, and disease associated CNVs), only two datasets (HapMap 270 samples and 400 MGL samples) have data for chromosome Y, which are also very sparse. Therefore, we focused on comparing association statistics between chromosome X and autosomes. We recalculated the enrichment of various structural mutabilities in the methylation deserts, comparing autosomes vs. chrX. As summarized in Table S9, methylation deserts in the autosomal chromosomes showed significant enrichment of structural mutabilities. Although those in chromosome X also show relative enrichment, the p-values are not as significant, which indicates the overall association could not be explained by sex chromosome bias.

4. Examination of features in windows with MI=0

We constructed a methylation index (MI) map for the human germline by dividing the whole human genome into non-overlapping 100Kbp windows, and by calculating MI scores for each window. We found approximately 1.5% of these windows have zero MI scores, indicating hypomethylation in human germline.

Investigation of CpG distribution, GC content, SINEs, LINEs and microsatellites showed that there are no particular enrichment of these features in the windows with MI=0 compared with randomly selected regions (Figure S19ABE). These windows do show average lower number of SNPs than other regions (Figure S19C). However the whole-genome bisulfite sequencing data of the human sperm DNA exclude SNP density bias as the cause of the difference in methylation levels (Materials and Methods section 3.2, Figure S6). Comparison of sequence conservation in these windows with other regions presents an interesting pattern. We first examined the average sequence conservation score using base-pair resolution mapping of 44 vertebrates from UCSC database (http://hgdownload.cse.ucsc.edu/goldenPath/hg18/phastCons44way/). The windows with MI=0 show much higher average conservation score than other windows (Figure S19D, KS test D = 0.36, p-value < 2.2e-16), indicating sequence in these windows to be relatively highly conserved. In addition, those regions also hypomethylated in sperm (<5%) are showing higher sequence conservation than those regions not hypomethylated in sperm (>5%) (KS test D = 0.25, p-value < 1.0e-02). However, when we focus on coding sequences, the windows with MI=0 are slightly deprived of conserved genes (0.9 fold), and genes are overall under-enriched (0.7 fold). Enrichment was observed for pseudogenes (2 fold), which indicates that the genes in these regions may be evolving faster than regions with higher methylation levels. Functional clustering analysis of genes in these regions showed that they are highly enriched for leucine-rich repeats (PRAME family) and defensin functional genes, and are deprived of genes functional for cytoplasmic vesicle and cell adhesion (Table S4). Genetic disorders or phenotype categorical analyses of these regions showed that they are enriched for loci linked to mental retardation (Table S5), however, statistical significance was lost after correcting for multiple testing.

5. Comparison of CNVs detected in 400 MGL samples using custom Agilent array and CNVs detected in 270 HapMap samples using Affymetrix 6.0 chip

We examined evidence that our analysis may be affected by biases in array design and by selection acting on structural mutations. Toward this goal we compared CNVs detected in the 270 HapMap samples using high-resolution Affymetrix SNP 6.0 chip [10] with the CNVs detected in the 400 MGL samples using custom-designed Agilent array. We examined the distribution of structural heterozygosity rates and enrichment for specific functional gene annotations.

Despite the bias away from known polymorphisms in the design of the Agilent array, the distribution of the CNV heterozygosity rate obtained using Agilent array on 400 MGL samples is similar to that obtained on 270 HapMap samples using the Affymetrix array (Figure S12AB).

The intersection of the CNV locations with RefSeq gene coordinates revealed under-representation of genes in the CNV regions for both arrays (270 HapMap samples-0.57 fold, 400 MGL samples-0.68 fold, permutation test, $p<10^{-3}$). This

may indicate either purifying selection or a bias of hypermutable regions away from gene-rich loci.

We next examined possible positive or balancing selection by examining enrichment for specific functional annotations of genes at various structural heterozygosity levels. The results indicate detectable enrichment for specific categories suspected to be under positive or balancing selection (Figure S12CD). The two arrays obviously differ in that highly polymorphic gene loci were mostly avoided in the design of the Agilent array.

6. Individuals carrying more CNVs also have higher concentration of CNVs within methylation deserts

We explored inter-individual differences in structural mutability. Specifically, we tested whether the individuals who carry more CNVs also tend to have a higher concentration of CNVs within hypomethylated regions identified in the 2.5X methylome. Each of the 400 MGL samples was assigned a variability score equal to the total number of CNVs detected in the sample. Each CNV was then assigned the same variability score as the sample in which it was detected (Figure S17A). The distribution of variability scores for CNVs within methylation deserts was then compared to the distribution of the variability scores for CNVs outside of methylation deserts. The former had significantly higher variability scores (Figure S17B), indicating that excess variants within hypermutable samples tend to be concentrated within methylation deserts. A similar pattern was observed for the windows with MI=0 (Figure S17C). This result could not be confirmed using 15X methylomes.

Reference:
1. Bailey JA, Yavor AM, Massa HF, Trask BJ, Eichler EE (2001) Segmental duplications: organization and impact within the current human genome project assembly. Genome Res 11: 1005-1017.
2. Zhang F, Gu W, Hurles ME, Lupski JR (2009) Copy number variation in human health, disease, and evolution. Annu Rev Genomics Hum Genet 10: 451-481.
3. Sharp AJ, Hansen S, Selzer RR, Cheng Z, Regan R, et al. (2006) Discovery of previously unidentified genomic disorders from the duplication architecture of the human genome. Nat Genet 38: 1038-1042.
4. Redon R, Ishikawa S, Fitch KR, Feuk L, Perry GH, et al. (2006) Global variation in copy number in the human genome. Nature 444: 444-454.
5. Bailey JA, Gu Z, Clark RA, Reinert K, Samonte RV, et al. (2002) Recent segmental duplications in the human genome. Science 297: 1003-1007.
6. Stankiewicz P, Lupski JR (2002) Genome architecture, rearrangements and genomic disorders. Trends Genet 18: 74-82.
7. Pacheco SE, Houseman EA, Christensen BC, Marsit CJ, Kelsey KT, et al. (2011) Integrative DNA methylation and gene expression analyses identify DNA packaging and epigenetic regulatory genes associated with low motility sperm. PLoS One 6: e20280.
8. H. Bengtsson KS, J. Bullard & K. Hansen (2008) aroma.affymetrix: A generic framework in R for analyzing small to very large Affymetrix data sets in bounded memory.
9. Merviel P, Heraud MH, Grenier N, Lourdel E, Sanguinet P, et al. (2010) Predictive factors for pregnancy after intrauterine insemination (IUI): an analysis of 1038 cycles and a review of the literature. Fertil Steril 93: 79-88.
10. McCarroll SA, Kuruvilla FG, Korn JM, Cawley S, Nemesh J, et al. (2008) Integrated detection and population-genetic analysis of SNPs and copy number variation. Nat Genet 40: 1166-1174.
